# Sleep State Modulates Resting-State Functional Connectivity in Neonates

**DOI:** 10.3389/fnins.2020.00347

**Published:** 2020-04-17

**Authors:** Chuen Wai Lee, Borja Blanco, Laura Dempsey, Maria Chalia, Jeremy C. Hebden, César Caballero-Gaudes, Topun Austin, Robert J. Cooper

**Affiliations:** ^1^neoLAB, The Evelyn Perinatal Imaging Centre, The Rosie Hospital, Cambridge University Hospitals NHS Foundation Trust, Cambridge, United Kingdom; ^2^Neonatal Intensive Care Unit, The Rosie Hospital, Cambridge University Hospitals NHS Foundation Trust, Cambridge, United Kingdom; ^3^DOT-HUB, Department of Medical Physics and Biomedical Engineering, Faculty of Engineering Sciences, University College London, London, United Kingdom; ^4^Basque Center on Cognition, Brain and Language, Donostia/San Sebastián, Spain

**Keywords:** resting-state functional connectivity, functional near-infrared spectroscopy, sleep state, neonates, connectome, functional imaging

## Abstract

The spontaneous cerebral activity that gives rise to resting-state networks (RSNs) has been extensively studied in infants in recent years. However, the influence of sleep state on the presence of observable RSNs has yet to be formally investigated in the infant population, despite evidence that sleep modulates resting-state functional connectivity in adults. This effect could be extremely important, as most infant neuroimaging studies rely on the neonate to remain asleep throughout data acquisition. In this study, we combine functional near-infrared spectroscopy with electroencephalography to simultaneously monitor sleep state and investigate RSNs in a cohort of healthy term born neonates. During active sleep (AS) and quiet sleep (QS) our newborn neonates show functional connectivity patterns spatially consistent with previously reported RSN structures. Our three independent functional connectivity analyses revealed stronger interhemispheric connectivity during AS than during QS. In turn, within hemisphere short-range functional connectivity seems to be enhanced during QS. These findings underline the importance of sleep state monitoring in the investigation of RSNs.

## Introduction

In recent years, the number of studies investigating resting-state networks (RSNs) in neonates has grown immensely (Gao et al., 2016). While most of these studies utilize functional connectivity MRI (fcMRI) to identify RSNs (Fransson et al., 2007; Doria et al., 2010; Smyser et al., 2010; Damaraju et al., 2014), functional near-infrared spectroscopy (fNIRS) has emerged as a promising modality due to its practical advantages, which include portability, low-cost, and silent operation. fNIRS studies investigating resting-state functional connectivity in infants have demonstrated: (1) evidence of primary sensory RSNs such as the visual (White et al., 2012) and auditory RSNs in term neonates (Ferradal et al., 2016), (2) increasing interhemispheric connectivity between homotopic regions in the first 6 months of life (Homae et al., 2010), and (3) altered functional connectivity patterns in preterm born neonates at term age (Fuchino et al., 2013; Naoi et al., 2013).

A significant challenge in studying neonates, common to both fcMRI and optical modalities, is subject motion. To minimize motion, neonates are almost always scanned whilst asleep, or even under light sedation (Arthurs et al., 2012). However, the effect of sleep state on RSNs has not been directly investigated in the neonatal population, despite evidence of altered activity in adults. In adults, networks associated with primary functions (such as the sensorimotor network) and cortico-cortical connectivity strength have both been shown to diminish during deep non-rapid eye movement (NREM) and slow wave sleep (Spoormaker et al., 2010; Watanabe et al., 2014), but are preserved in early, light NREM sleep (Horovitz et al., 2008; Larson-Prior et al., 2009). The different stages of NREM sleep can also have variable effects on higher cognitive function RSNs such as the default mode network (DMN), where different contributions from each region decrease with progressive sleep depth (Horovitz et al., 2009; Sämann et al., 2011). Furthermore, sedation is known to produce a variable effect on high-order cognitive RSNs in adults (Greicius et al., 2008; Martuzzi et al., 2010).

Monitoring arousal or sleep state in neonates is routinely performed with electroencephalography (EEG; Dereymaeker et al., 2017; Pillay et al., 2018). However, introducing additional neuro-monitoring equipment into a study of an already challenging population can be time consuming and problematic. Combining EEG and fcMRI is a significant technical challenge in any population, but particularly in neonates (Arichi et al., 2017). Because of the duration of the infant sleep cycle, if one wishes to investigate functional networks during multiple sleep states in a single neonate, it is necessary to monitor that subject for an extended period. Scanning for prolonged study durations can pose significant challenges for vulnerable neonates using modalities such as fcMRI. A more promising approach therefore is to combine EEG with multichannel fNIRS. Both modalities are portable and share a common set-up procedure that allows for simultaneous application of both devices to an infant subject. EEG offers the ability to distinguish sleep states in neonatal RSN studies. In the full-term neonate, the sleep cycle lasts between 30 and 70 min and comprises largely of so-called “active” and “quiet” sleep segments (Scher, 2008). The temporal order of active sleep (AS) and quiet sleep (QS) is variable in term neonates, and during a full sleep cycle, AS and QS can occur more than once. Typically, the sleep cycle starts with a period of mixed frequency AS, however, this only occurs in over 50% of infants. Short stages of transitional or indeterminate sleep patterns can also occur where physiology and behavior are not characteristic of either AS or QS (Scher, 2008). AS in infants is typically characterized by a continuous EEG background. Associated physiological observations include rapid eye movements, increased cardiorespiratory variability, low muscle tone, and frequent body movements. QS is characterized by either a continuous, slow, high-voltage EEG or a discontinuous EEG pattern also known as tracé alternant. The latter is defined by bursts of high amplitude slow-wave activity interspersed with low voltage activity. The absence of eye movements, reduced respiratory variability, increased muscle tone, and reduced body movements are also characteristic features.

In this study, we combined standard, neonatal clinical montage EEG with a multichannel fNIRS system with a source-detector (SD) array covering the temporal, parietal, and inferior frontal regions of the head in a relatively high-density arrangement. We measured spontaneous cortical hemodynamic activity with fNIRS in a group of healthy term born neonates during AS and QS. We employed three independent analysis methods to assess the influence of sleep state in resting-state functional connectivity. Using a seed-based correlation analysis we investigated the influence of sleep state in network structures such as the frontal, auditory, and sensorimotor RSNs which in this age group are expected to demonstrate a developing bilateral homotopic connectivity (Fransson et al., 2007; Doria et al., 2010; Homae et al., 2010; Smyser et al., 2010; Ferradal et al., 2016). By using two connectome-based approaches we also investigated the interaction between neonatal sleep state and the spatial organization of large-scale resting-state functional connectivity patterns.

## Materials and Methods

### Subjects

Healthy term neonates (>37 weeks of gestation) were recruited from the postnatal ward of The Rosie Hospital (Cambridge University Hospitals NHS Foundation Trust). This study was approved by the National Research Ethics Service Committee East of England (REC reference 13/EE/0344), and written informed consent was obtained from parents for neonates to participate. A total of 30 neonates were recruited. Datasets from 10 neonates were excluded during data processing due to low data quality or insufficient duration of AS and QS data segments for subsequent RSN analysis. fNIRS data was analyzed from the remaining 20 subjects (11 males and 9 females; mean gestational age = 40 + 2 weeks; mean weight at birth = 3704 g; mean scan age = 2.65 days). Demographic details of the subjects are summarized in Table 1.

**TABLE 1:** Demographic details of the 20 subjects included in the fNIRS resting-state analysis.

**Subject**	**GA at birth (weeks)**	**Scan age (days)**	**Weight (g)**	**Gender**	**AS recording duration (s)**	**QS recording duration (s)**
1	38 + 0	3	2830	M	121	121
2	38 + 0	3	3165	M	126	182
3	41 + 5	2	3500	F	153	367
4	39 + 5	2	3890	F	124	124
5	40 + 6	1	3770	F	150	156
6	40 + 2	1	3440	M	155	153
7	40 + 6	2	3820	F	147	475
8	40 + 0	5	3585	F	161	180
9	41 + 6	3	3680	F	214	183
10	39 + 2	6	3450	M	590	534
11	39 + 0	2	3500	F	167	290
12	42 + 3	1	3960	M	148	171
13	39 + 0	4	3375	M	123	180
14	39 + 1	1	3980	M	136	654
15	40 + 4	1	3770	F	209	204
16	42 + 0	1	4270	F	141	333
17	41 + 2	2	4140	M	120	283
18	41 + 2	6	4180	M	121	150
19	41 + 0	2	4830	M	177	209
20	40 + 2	5	2935	M	128	225
					170.5 ± 102	259 ± 146

*Recording duration for the analyzed AS and QS periods is also included. AS, active sleep; QS, quiet sleep; GA, gestational age; M, male; F, female.*

### Data Collection

**FIGURE 1:**
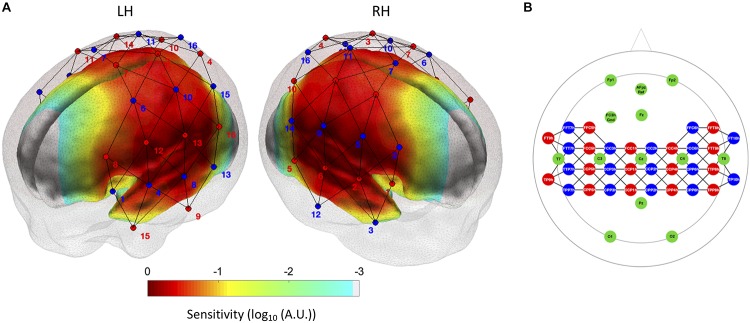
**(A)** Optode localization (sources in red and detectors in blue) in the current experimental setup. The normalized sensitivity profile of this configuration is displayed on a neonate head model template (Brigadoi et al., 2014), where regions of higher sensitivity below the source-detector configuration are displayed in red color. **(B)** fNIRS optode and EEG electrode positions in the 10–5 system for the EasyCap montage employed in the current study. The fNIRS sources are indicated in red, the detectors in blue, and the EEG electrodes in green.

**FIGURE 2:**
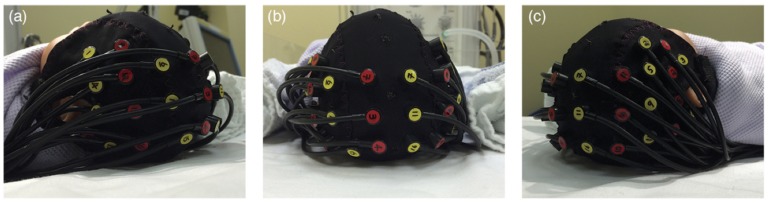
fNIRS-EEG cap arrangement showing the positioning of fNIRS optodes (sources in red, detectors in yellow) on an infant head from **(a)** left, **(b)** top and **(c)** right views.

Neonates were imaged in a quiet, dimly lit room in The Evelyn Perinatal Imaging Centre (Cambridge University Hospitals NHS Foundation Trust). To promote sleep, a feed and wrap approach was used. As a full-sleep cycle in the neonatal period typically lasts for up to an hour (Scher, 2008), imaging sessions were performed for at least 1 h to capture both AS and QS. We used the NTS fNIRS System (Gowerlabs Ltd., United Kingdom), which is a continuous-wave multichannel fNIRS device (Everdell et al., 2005) with a sampling rate of 10 Hz. The system consists of 16 dual-wavelength laser-diode sources operating at near-infrared wavelengths of 780 nm and 850 nm, and 16 avalanche photodiode detectors. A SD array with 69 measurement channels consisting of SD separation distances ranging from 19 to 36 mm was designed to cover the inferior frontal, temporal, and parietal regions of the cortex (Figure 1A). A customized EEG head cap (EasyCap, GmbH) was adapted to hold the SD array in accordance with a subset of standard 10–5 positions (Oostenveld and Praamstra, 2001). Nasion, inion, and preauricular points were used as external head landmarks for cap positioning. A standard neonatal montage (Walls-Esquivel et al., 2007) consisting of 11 EEG electrodes (Fp1, Fp2, Fz, T3, T4, C3, C4, Cz, Pz, O1, and O2) with two of the electrodes assigned for ground (FC3h) and reference (AFpz) were incorporated into the EasyCap (Figure 1B). The cap was secured on the infant subject’s head using a soft elastic chinstrap and a chest belt to maintain SD fiber coupling to the scalp (see Figure 2 for an example of our cap arrangement on a representative participant). EEG abrasion gel and conduction paste were applied to minimize EEG electrode impedances to below 5 kΩ. The EEG setup also included electrocardiography (ECG), behavioral observation, and EEG video. To synchronize the fNIRS and EEG signals, an external event generator device was used (Gowerlabs Ltd., United Kingdom).

### EEG Sleep State Assessment

The EEG data were visualized offline using BrainVision Analyzer V.2.0.2 software (Brain Products GmbH, Germany), and the EEGLAB toolbox (Delorme and Makeig, 2004) for MATLAB (MathWorks, United States). Standard filtering was applied to the EEG signal using a high-pass filter at 0.5 Hz and a low-pass filter at 70 Hz with an added notch filter at 50 Hz (United Kingdom mains frequency). Each electrode was referenced to the common reference electrode positioned at AFpz.

The degree of continuity and amplitude of the EEG signal were used to visually categorize EEG segments into AS and QS using standard criteria (Scher, 2008; André et al., 2010; Grigg-Damberger, 2016). Segments of mixed frequency or low amplitude continuous EEG were labeled as AS. High-voltage continuous or tracé alternant segments were labeled as QS. ECG variability and infant movement observed and recorded on EEG video were used to complement the EEG signal in determining sleep state. Any segments in which sleep state was undetermined and those that contained motion artifacts were excluded from further analysis. Segments in which head movements were evident, as determined by visual inspection of the EEG video, were marked as motion in both EEG and fNIRS data. In addition, a 5-s mask was added at the beginning and at the end of each identified motion segment. Any other movement of the participant that was visible on EEG video was cross-referenced with the fNIRS signal and marked as motion if temporally correlated motion artifacts were observed. Sleep segments were also visually identified in a subset of infants by a second observer to confirm inter-rater agreement (Cohen’s kappa = 0.833).

### fNIRS Data Preprocessing

The fNIRS and EEG datasets for each subject were synchronized using the trigger events in both signals produced from the event generator device. The time markers for the labeled EEG sleep segments were transformed to the fNIRS time-base to enable the division of the fNIRS data into AS and QS segments. fNIRS data were excluded as noise if the measured light intensity was below 5e-4 a.u. (arbitrary units), as recommended by the manufacturer. On top of the data exclusion criteria described in the previous section we also excluded data segments based on the fNIRS signal using the *hmrMotionArtifact* function in Homer2, Huppert et al. (2009). Any change in measured optical density (OD) occurring within any 0.5 s period that was greater than 0.5 OD, or greater than 15 times the standard deviation of the entire time-course was considered motion artifact. Five seconds of data before the start and end of each segment marked as motion artifact were excluded. The longest motion-free segments from within each fNIRS sleep segment were extracted for the functional connectivity analyses. Previously reported infant RSN fNIRS studies used a minimum duration of 2 min of data (White et al., 2012; Bulgarelli et al., 2019, 2020), therefore the longest motion-free segment of at least 2 min duration was selected for AS and QS. The mean duration of fNIRS data segments was 215 s (AS = 170 ± 102 s; QS = 258 ± 146 s). From these segments, changes in OD were calculated from raw optical intensity data using the *hmrIntensity2OD* algorithm implemented in Homer2. OD data was converted into oxyhemoglobin (HbO) and deoxyhemoglobin (HbR) concentration changes (*hmrOD2Conc* in Homer2), using differential pathlength factors 5.3 and 4.2 (Scholkmann and Wolf, 2013). Temporal filtering and global signal regression were performed simultaneously in a unique regression model. Sine and cosine functions for frequencies above 0.08 Hz were included in the model to remove the contribution of physiological noise sources (e.g., respiration and cardiac pulsation), and up to 4th order Legendre polynomials (depending on dataset duration) were included to account for fluctuations at very low frequencies. The average fNIRS signal across channels was included in the regression model, for HbO and HbR independently, to remove globally occurring hemodynamic signals, which were assumed to largely reflect systemic changes (White et al., 2009; Mesquita et al., 2010; Tachtsidis and Scholkmann, 2016). Quality assurance figures for each participant at different steps of the pre-processing, are available as Supplementary Material.

### Resting-State Functional Connectivity Analyses

**FIGURE 3:**
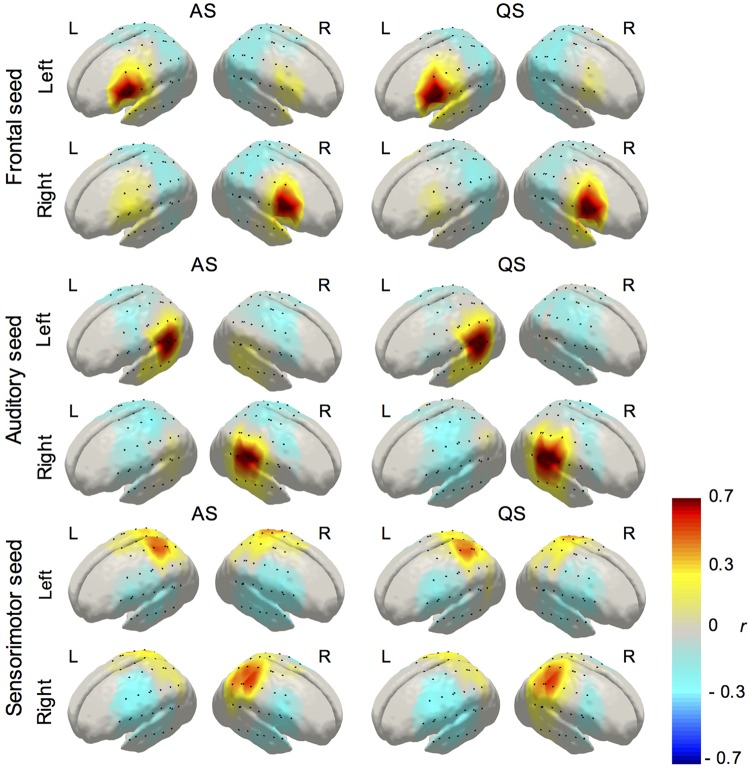
Average correlation maps (HbO) derived from the seed-based correlation method for the left and right seeds in the inferior frontal, auditory, and sensorimotor regions (AS, active sleep, first column; QS, quiet sleep, second column). Due to its high similarity, and to avoid presenting redundant information, HbO maps are displayed in the main text and HbR maps are presented as Supplementary Material.

All the analyses described below were performed for both HbO and HbR. First, a seed-based correlation analysis approach was adopted to create correlation maps for AS and QS conditions and for each infant. Seed channels for the left and right hemispheres were selected for the inferior frontal, auditory, and sensorimotor regions based on previously published reports (Doria et al., 2010; Smyser et al., 2010; Ferradal et al., 2016). A robust Pearson’s correlation coefficient (Santosa et al., 2017) was calculated between each seed channel and every other channel in the array. Individual correlation values were normalized by Fisher’s *Z* transformation before averaging across subjects. Average values were then transformed back to Pearson’s correlation coefficients for the presentation of average correlation maps corresponding to each sleep state and for each region (Figure 3). For each seed region, group differences between sleep states were assessed by conducting channel-wise paired *t*-tests between AS and QS conditions, which were corrected for multiple comparisons using the false discovery rate (FDR; *q* < 0.05) method (Benjamini and Hochberg, 1995). For results visualization purposes, we computed the sensitivity profile of our fNIRS sensor setup. We registered our sensors to the MRI space of a neonate head model template (Brigadoi et al., 2014) and calculated the sensitivity matrix of our SD configuration using *Toast++* (Schweiger and Arridge, 2014). We summed the cortical sensitivity profiles of each channel, resulting in the aggregated sensitivity profile of our sensors. Channel positions were defined as the gray matter node which coordinates were closest to the central point of the maximum sensitivity path along each SD pair.

Between-group statistical comparisons of functional connectivity matrices were also performed using two connectome-based approaches. First, we followed a Network Based Statistic (NBS) approach, a widely employed method in fcMRI studies to perform group-level comparisons in structural and functional brain network organization (Zalesky et al., 2010, 2012; García-Pentón et al., 2014). In this approach, a statistical test of group differences (i.e., a *t*-test) is computed on the subjects’ functional connectivity matrices for each channel pair, and clusters of interconnected edges above a preselected statistical threshold are identified. In this study, we evaluated two thresholds (*t*-values) representing different levels of statistical significance: *t*_1_(19) = 2.6, equivalent to *p* = 0.01 and *t*_2_(19) = 3.6, equivalent to *p* = 0.001. These values represent the thresholds for which the null hypothesis will be rejected at each channel pair – same approach as a massive univariate analysis – and therefore have an impact on network size (i.e., total number of interconnected edges exceeding the statistical threshold), but not on the significance of the identified networks. This is tested using non-parametric permutation testing, controlling the family-wise error rate at the network level based on the network size. Second, we used connICA (Amico et al., 2017), a data-driven methodology based on independent component analysis (ICA), which can be used to extract group-level independent functional connectivity patterns from a set of individual functional connectivity matrices. This methodology also provides a framework to study potentially meaningful associations between a particular experimental variable (e.g., levels of consciousness in Amico et al., 2017) and the prominence of specific functional connectivity patterns representing distinct functional processes. We applied the NBS and connICA methods to HbO and HbR derived functional connectivity matrices, and the results for both chromophores are reported.

## Results

During EEG and fNIRS data processing, it was observed that AS data typically contain a larger number of motion artifacts, and were therefore shorter in duration compared to QS on average (mean duration for AS and QS were 170 ± 102 s and 258 ± 146 s, respectively). A paired two-tailed *t*-test revealed a significant difference; *t*(19) = −2.8289, *p* = 0.0107. This was expected as AS is associated with frequent body movements, while in QS body movements are reduced (Scher, 2008). However, we also assessed data quality across sleep states by comparing the amount of motion on each group, based on the kurtosis of wavelet coefficients of the selected segments (Chiarelli et al., 2015). This provides a measure of the abundance of outliers apparent in each dataset; outliers that are likely to represent transient noise sources, including any remaining motion artifact. No difference in wavelet kurtosis was evident between the AS and QS groups [*k*_AS_ = 3.41 ± 1.23, *k*_QS_ = 3.69 ± 2.43, *t*(19) = −0.49, *p* = 0.6240].

**FIGURE 4:**
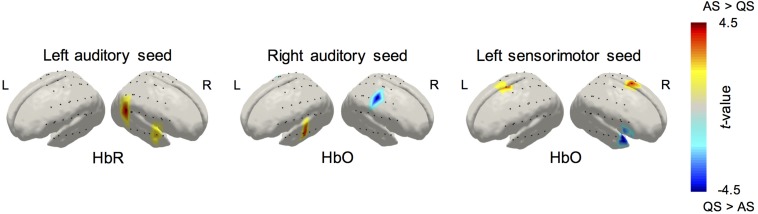
Group differences in RSNs revealed by the paired *t*-tests of the seed-based correlation method between active and quiet sleep. Results for HbO and HbR are displayed for those statistical tests that survived the seed level FDR correction criterion (*q* < 0.05).

The average correlation maps produced from the seed-based correlation analysis illustrating functional connectivity for the left and right seeds in the inferior frontal, temporal, and parietal regions during AS and QS, are displayed in Figure 3. Connectivity maps for all the seed regions showed the expected spatial configuration in the two hemodynamic contrasts (i.e., HbO and HbR), with the strongest correlations being observed on channels surrounding the seed regions and in bilateral homologous seed-regions respectively. Qualitative assessment of group maps for the AS condition implied bilateral connectivity for both left and right seeds in the inferior frontal, auditory, and sensorimotor regions. In contrast, during QS bilateral connectivity appears reduced in the inferior frontal areas and absent in the auditory regions. Similar results were observed in the seed-based correlation analysis for HbR, which are available as Supplementary Material. The channel-wise paired *t*-tests results for the comparison between AS and QS for HbO and HbR are displayed in Figure 4. Only statistically significant differences that survived multiple comparison correction using the FDR criterion (*q* < 0.05) are reported. Overall these results show that the correlation between homologous channels across hemispheres is stronger in AS on the left and right auditory seeds and on the left sensorimotor seed. In turn, during QS, correlation was stronger within nearby channels on the right auditory seed, and between the left motor seed and channels on the right inferior frontal region.

**FIGURE 5:**
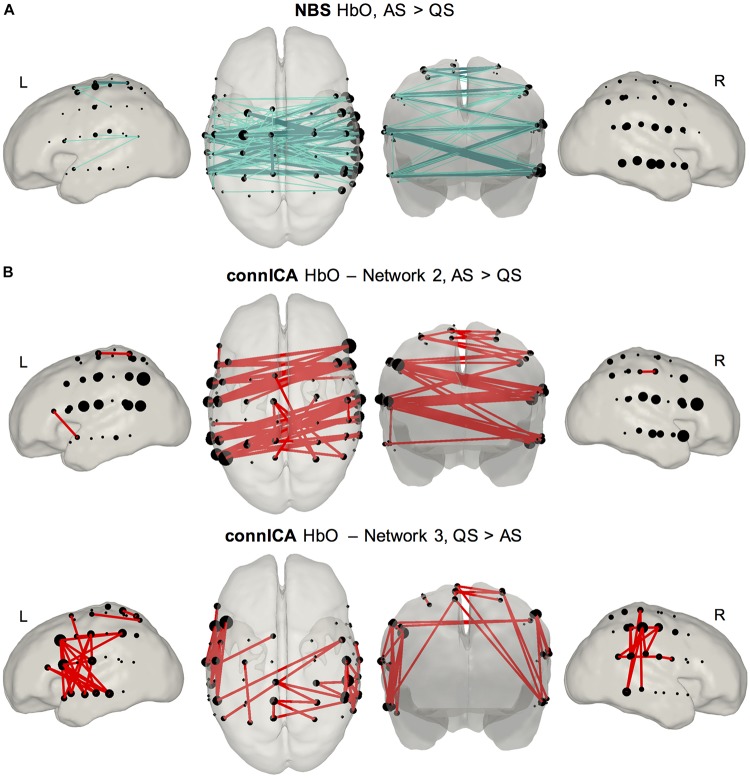
Results of connectome based analyses revealing group differences between sleep states. **(A)** NBS method (top row) showed a network mainly characterized by interhemispheric connectivity which was stronger in AS compared to QS. Results are presented for two statistical thresholds (*t*_1_, *p* < 0.001 empirical permutation test, light color, thin edges; *t*_2_, *p* < 0.005 empirical permutation test, dark color, thick edges). This difference was only observed for HbO. **(B)** ConnICA method (second and third rows) revealed two networks showing significant differences between sleep states. Network 2 represents a functional connectivity pattern formed by interhemispheric edges showing a higher prominence during AS [*t*(19) = 3.19; *p* = 0.0048]. Network 3 is a RSN characterized by short-range unilateral edges displaying stronger connectivity during QS [*t*(19) = –3.09; *p* = 0.006]. These network level differences were observed for both HbO and HbR. Due to the high spatial similarity between the observed HbO and HbR networks only figures for HbO are presented in the main text, and HbR figures are presented as Supplementary Material.

The NBS analysis also revealed significant group differences between sleep states in global functional connectivity organization (Figure 5A). For HbO, and at the two statistical thresholds considered, we found a functional network showing higher synchronization in AS periods, as opposed to QS periods. This network was significant, evaluated using non-parametric permutation testing, for the two statistical thresholds considered (*t*_1_, *p* < 0.001 and *t*_2_, *p* < 0.005, corrected). The topological organization of this network demonstrates increased interhemispheric connectivity during AS. The strongest connections (which are represented as dark colored, thick edges in Figure 5A) were observed along areas corresponding to bilateral auditory and sensorimotor regions. No significant network differences were observed for increased connectivity in QS as compared to AS for HbO, nor for the HbR analysis in either direction.

The modulation in functional connectivity resulting from variations in sleep state was also confirmed using connICA (Figure 5B). The connICA method revealed seven group-level functional connectivity patterns characterizing our set of individual functional connectivity matrices that included AS and QS. This method also provided a vector of values quantifying the presence of each group-level functional connectivity pattern within each individual. It is these values that were employed to assess statistical differences between our experimental groups. In both HbO and HbR, two of these independent functional connectivity patterns were associated with significant sleep state modulations. The first network, which demonstrated a high degree of overlap with that extracted in the NBS analysis, mainly involved interhemispheric connectivity and showed a significantly higher prominence in neonates during AS [*t*(19) = 3.19; *p* = 0.0048]. The second network, characterized by within hemisphere short-range connections between nearby channels, showed a higher presence during QS [*t*(19) = −3.09; *p* = 0.006]. These two functional networks are threshold to show only positive connections (top 5% of connections), as the interpretation of negative correlations in functional connectivity analyses still remains contentious. The complete Figures – with positive and negative connections – of all the functional components calculated with connICA for HbO and HbR are available as Supplementary Material.

## Discussion

This study demonstrates the utility of combining multichannel fNIRS with EEG to simultaneously monitor sleep state and RSNs in neonates. The current work also represents the first demonstration of spatial pattern variations in RSNs between different sleep levels in neonates. We used three independent analysis methods to quantitatively compare resting-state functional connectivity between AS and QS in a cohort of healthy neonates, as each of them provides complementary information to investigate associations between sleep state and group-level network characteristics. NBS compares global network characteristics between experimental groups, but only networks showing differences between the groups are extracted. In contrast, connICA extracts multiple independent subcomponents from the functional connectivity patterns computed at the group-level, and then allows testing for statistical differences on the relevance of each component between groups. Our results for the three methods showed that interhemispheric long-range connectivity is stronger during periods of AS. In addition, the SBA and connICA methods revealed functional connectivity patterns in which local short-range connectivity is enhanced during QS.

These findings provide evidence that sleep state can modulate RSNs in the newborn brain and highlight the importance of monitoring sleep in studies of infant RSNs. Although prior studies have reported the presence of RSNs in neonates as early as 27 weeks of gestation (Smyser et al., 2010), several discrepancies exist in the observation of RSNs in the literature, both within and across imaging modalities (i.e., fcMRI or optical methods). For example, using fcMRI, Doria et al. (2010) observed complete RSNs (several of which were integrated with thalamic activity) in preterm infants scanned at term-equivalent age. These were indistinguishable from those observed in term-born infants. Smyser et al. (2010) also observed recognizable RSNs using fcMRI in preterm infants that developed with age. However, by term equivalent age, these RSNs demonstrated lower correlation, limited spatial distribution, and reduced thalamocortical connectivity as compared to term-born controls. Using diffuse optical tomography (DOT), White et al. (2012) reported interhemispheric connectivity in the occipital cortex reflecting visual RSNs in healthy term infants. On the other hand, in a RS fNIRS study, Homae et al. (2010) showed a reduced interhemispheric connectivity in their healthy term cohort, which was only observed across all brain regions at a later stage from 3 months of age, although these effects might be caused by contamination of the optical signal from physiological artifacts in the superficial layers of the head. However, none of these studies have formally monitored the sleep states of their subjects. This detail is particularly important since neuronal interactions as characterized by phase and amplitude dynamics in EEG have been reported to change significantly between sleep states in term born infants (Tokariev et al., 2016). While most RSN studies acquire data immediately within 30 min of sleep (Gao et al., 2016), the variability in sleep state and lack of sleep monitoring could also explain some of the inconsistencies observed across neonatal studies.

The stronger interhemispheric connectivity observed during AS may highlight the importance of sleep for neural development during the neonatal period, complementing the current understanding of the role of AS in early neural development. Sleep states emerge in the third trimester and become distinguishable electrographically from 30 weeks gestational age (Visser et al., 1987). While there is a significant increase in QS duration (Mulder et al., 1987; Curzi-Dascalova et al., 1988, 1993), AS remains the predominant sleep state until term (Mulder et al., 1987; Mirmiran, 1995). In full-term neonates, more than half of sleep time is spent in AS (Mirmiran et al., 2003). The long periods of AS in early infancy occur concurrently with periods of rapid cerebral maturation, suggesting neural activity during AS may be functionally important in early development. Animal studies demonstrate that cerebral blood flow and oxygen delivery is relatively higher in AS compared to QS (Morrison et al., 2005), and that cerebral metabolic rate of oxygen consumption is as high in AS as during wakefulness (Silvani et al., 2006). In rat pups, brain mass including the cerebral cortex and brainstem are significantly reduced after AS deprivation, with alterations in neurotransmitter sensitivity compared to typically developing rats (Mirmiran et al., 1983; Morrissey et al., 2004). In AS, movements that are anticipatory in nature such as eye movements, stretches, and sucking occur frequently, therefore AS may facilitate neural development by providing endogenous stimulation at a time when waking life is limited with little exogenous stimulation (Roffwarg et al., 1966; Denenberg and Thoman, 1981). Spontaneous cerebral activity represented by RSNs may therefore reflect the endogenous activity of the brain during AS.

The ability to monitor RSNs in neonates could become a valuable clinical tool to assess cerebral function and development in vulnerable neonatal patients. This is of great clinical importance as the incidence of neurodisability remains unchanged despite advances in neonatal care and improvements in survival rates (Costeloe et al., 2012). It has been shown that preterm neonates exhibit reduced network complexity, a quantitative measure of network amplitude and dimensionality (Smyser et al., 2016). Complications related to prematurity, such as white matter injury (Smyser et al., 2013), hemorrhagic parenchymal infarction (Arichi et al., 2014), and exposure to stress and painful procedures (Smith et al., 2011) may affect RSN development. Furthermore, the impact of prematurity on sleep maturation may also lead to complications in RSN development. Sleep is the predominant behavioral state in the neonate, however, the busy environment of the NICU can disrupt sleep organization in both preterm and sick term neonates (Scher et al., 1992, 2002). As the sleep cycle is largely made up of AS compared to QS, interruptions to sleep due to intensive care are more likely to have an effect on the AS period therefore disrupting resting-state cerebral activity and potentially altering the development of RSNs.

While AS and QS can be broadly related to adult REM and NREM sleep respectively, comparisons between the sleep states of infants and adults need to be made with caution. In this study, the reduction in interhemispheric connectivity observed during QS is contrary to their preservation during NREM sleep in adults. This difference may be due to the significant maturational changes in sleep, especially of QS, in infants with advancing age (Mirmiran et al., 2003). Although standardized guidance is available for visual sleep scoring (Grigg-Damberger, 2016), the visual interpretation of AS and QS can vary between observers. A more objective approach could be to use quantitative EEG methods such as sleep state classifiers to identify sleep states (Paul et al., 2003; Piryatinska et al., 2009; Koolen et al., 2017). Extensive polysomnography is normally applied to formal clinical sleep studies, however, our set up (including ECG, behavioral observation, and EEG) was a practical one as it provided sufficient data to distinguish AS and QS with minimal handling of subjects.

It is difficult to capture a long continuous recording that is motion free in neonates, and this was one of the main limitations of this work. It is even more challenging if we are limited to a specific sleep state and even more so if there are segments of sleep states that are not easily identifiable as AS or QS. As a result of all this, the durations will inevitably be short. However, previous fNIRS resting-state functional connectivity studies reported using durations as short as 2 min on neonates (White et al., 2012), or 100 s in toddlers between 11 and 36 months of age (Bulgarelli et al., 2019, 2020). Wang et al. (2017) reported that 1-min recordings are enough to obtain accurate functional connectivity measures with fNIRS due its much faster temporal resolution. However, this study also confirmed that 7 min are necessary to obtain both accurate and stable measures. We also need to acknowledge the fact that, even with a higher sampling rate, the acquisition length will limit the spectral content of the fNIRS measurements. In particular, short acquisitions will fail to provide enough information in lower frequency ranges. In this study, and considering the need to allow at least one full cycle to resolve the lowest frequencies in the signal (Leonardi and Van De Ville, 2015), our 2 min measurements will lack enough information for robust estimation of functional connectivity at frequencies below 0.008 Hz (i.e., 1 full cycle/120 s). Although frequencies below 0.008–0.01 Hz are typically filtered out in resting-state functional connectivity analyses (Homae et al., 2010; White et al., 2012; Ferradal et al., 2016; Bulgarelli et al., 2020), longer recordings are still recommended, as they increase signal-to-noise ratio and reduce variability of the estimated functional connectivity (Hutchison et al., 2013). Another limitation of the current study is that the duration of AS and QS segments extracted for RSN analysis were significantly different. This was likely due to frequent subject motion associated with AS (Scher, 2008), which complicated the extraction of motion-free segments of sufficient duration. The presence of motion artifacts could lead to erroneous correlation results in RSN analysis (Santosa et al., 2017). However, this was not the case in the current study, as data quality was comparable across sleeps states.

Finally, the current fNIRS system uses a relatively dense SD array covering the temporal, parietal, and inferior frontal regions of the head. However, this limited us to investigating the inferior frontal, temporal, and sensorimotor RSNs. An ideal array configuration would achieve whole head coverage with a high sampling density and include short-distance channels to regress out systemic confounds. The shortest available SD distance in the current fNIRS setup was 19 mm, as we were limited to the physical size of the optodes (10 mm diameter) and we also allowed for placement of EEG electrodes. We acknowledge that 19 mm is already too large for short-separation regression, especially in infants (Brigadoi and Cooper, 2015). For this reason, we decided to follow a global signal regression approach to reduce physiological contamination (Pfeifer et al., 2018). With advances in technology, particularly the emergence of high-density, fiber-less DOT systems (Chitnis et al., 2016), it will soon be possible to produce high-quality, whole-cortex 3D images of cerebral hemodynamics at the cot-side, which will enable further investigation of neonatal RSNs.

## Conclusion

We used simultaneous fNIRS and EEG to investigate whether RSNs in neonates are affected by sleep state. Our results show that resting-state functional connectivity is differentially modulated across AS and QS states. Increased interhemispheric connectivity during AS, that is consistently observed across three independent analyses, may reflect the importance of AS in the functional organization of the developing brain in the absence of sensory stimulation. The prominence of long-range connectivity during AS compared to short-range connectivity during QS may indicate that each sleep state fulfils a different role during the early development of functional networks (Ouyang et al., 2017). This work highlights the importance of monitoring sleep in studies of neonatal resting-state functional connectivity. Furthermore, the presence of sleep state-dependent functional connectivity may demonstrate the developmental importance of sleep in the neonatal period more generally. Further research in this field, and the combination of optical methods and EEG techniques, may become clinically valuable, particularly in the cot-side neurological assessment of neonatal patients vulnerable to sleep disruption, such as those born preterm or with brain injury.

## Data Availability Statement

The datasets presented in this article are not readily available because the NHS data ethics covering this protocol date back to 2014, and only permit anonymised data sharing only within the immediate research group.

## Ethics Statement

The studies involving human participants were reviewed and approved by the National Research Ethics Service Committee East of England (REC reference 13/EE/0344). Written informed consent to participate in this study was provided by the participants’ legal guardian/next of kin.

## Author Contributions

CL conceived the study, designed the experiment, collected the data, performed the preliminary analyses, and wrote and edited the manuscript. BB designed and implemented the data analysis, determined the results, and wrote the manuscript. LD contributed to the design of the experiment and data collection, and edited the manuscript. MC contributed to the data collection, oversaw the EEG analysis, and edited the manuscript. JH oversaw the research collaboration, supervised LD, and edited the manuscript. CC-G supervised BB, oversaw the data analysis, and edited the manuscript. TA oversaw the research collaboration, supervised CL and MC, and edited the manuscript. RC oversaw the research collaboration, supervised CL, BB, and MC, and edited the manuscript.

## Conflict of Interest

RC has a financial interest in the UCL spin-out company Gowerlabs Ltd., which is a manufacturer of fNIRS technologies. The remaining authors declare that the research was conducted in the absence of any commercial or financial relationships that could be construed as a potential conflict of interest.
