# Supplementary material for: Sleep State Modulates Resting-State Functional Connectivity in Neonates
Source: Front Neurosci. 2020 Apr 17;14:347. doi: 10.3389/fnins.2020.00347 (PMC7180180; doi:10.3389/fnins.2020.00347)
Supplement: Supplementary file 1 [file Data_Sheet_1.PDF]

Four figures, showing the different steps of our data quality assessment routine are presented for each participant.
A brief explanation of these figures is provided below.

Figure 1 – Top row: This figure presents raw intensity data of the complete recording for the two wavelengths (WL) used in our fNIRS system. Periods of active and quiet sleep employed in the analyses are marked in light blue and red color respectively. **Middle and bottom rows** display the raw intensity data for each wavelength in the selected periods.

Figure 2: This figure shows the optical density (OD) data, concentration data (HbO & HbR) and the power spectral density (PSD) of concentration data for each experimental condition (active sleep & quiet sleep).

Figure 3: This figure shows, for each experimental condition, concentration data after filtering, the standard functional connectivity matrix and the functional connectivity matrix computed using robust regression.

Figure 4: For each experimental condition this figure shows preprocessed data after global signal regression, and the functional connectivity matrix of this data computed using robust regression. These matrices are the input for connectome-based data analyses (NBS and connICA).

HT_003

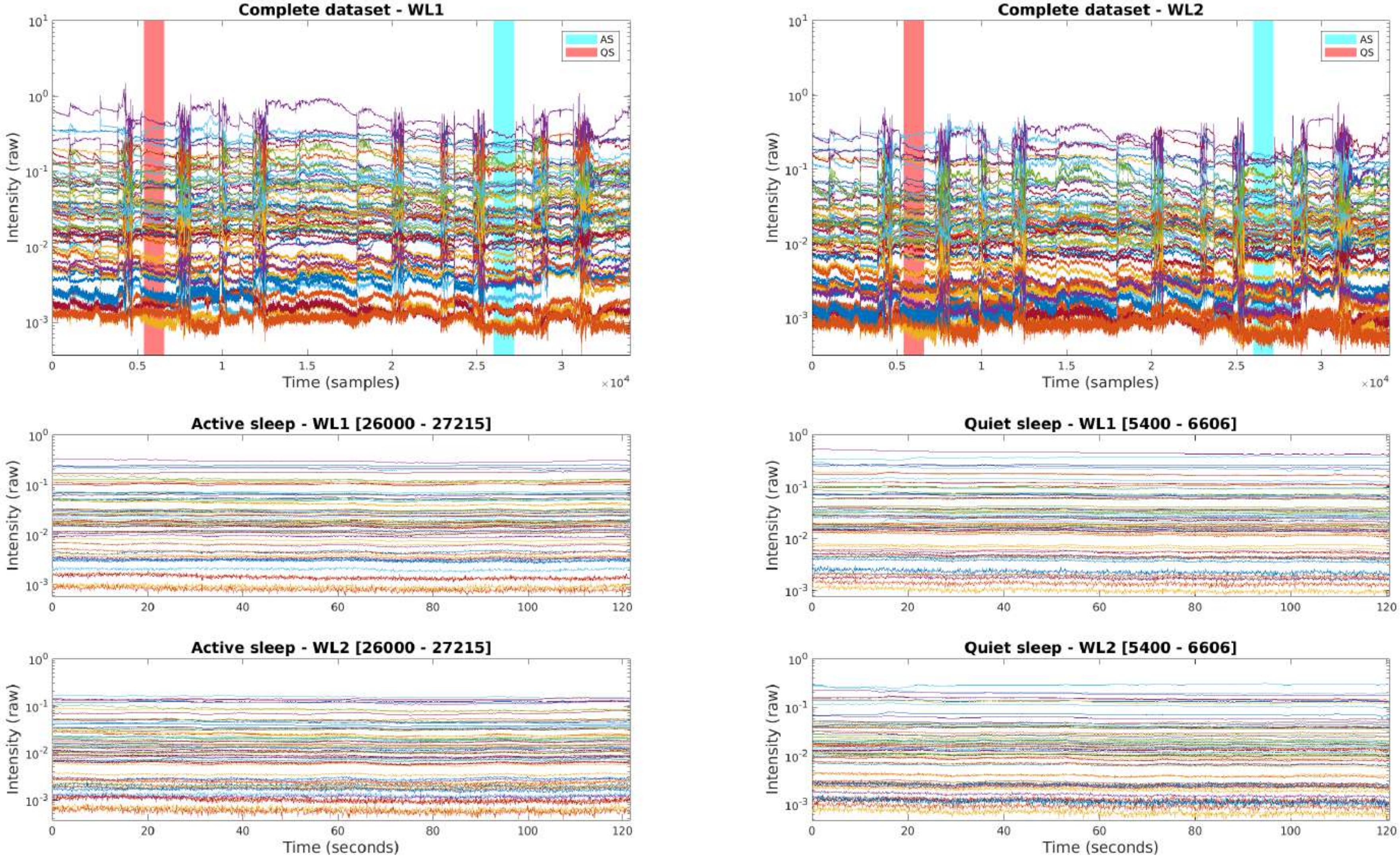
Complete dataset - WL1
Complete dataset - WL2
AS
QS
Intensity (raw)
Time (samples)
×10^4
Active sleep - WL1 [26000 - 27215]
Quiet sleep - WL1 [5400 - 6606]
Active sleep - WL2 [26000 - 27215]
Quiet sleep - WL2 [5400 - 6606]
Time (seconds)

HT_003

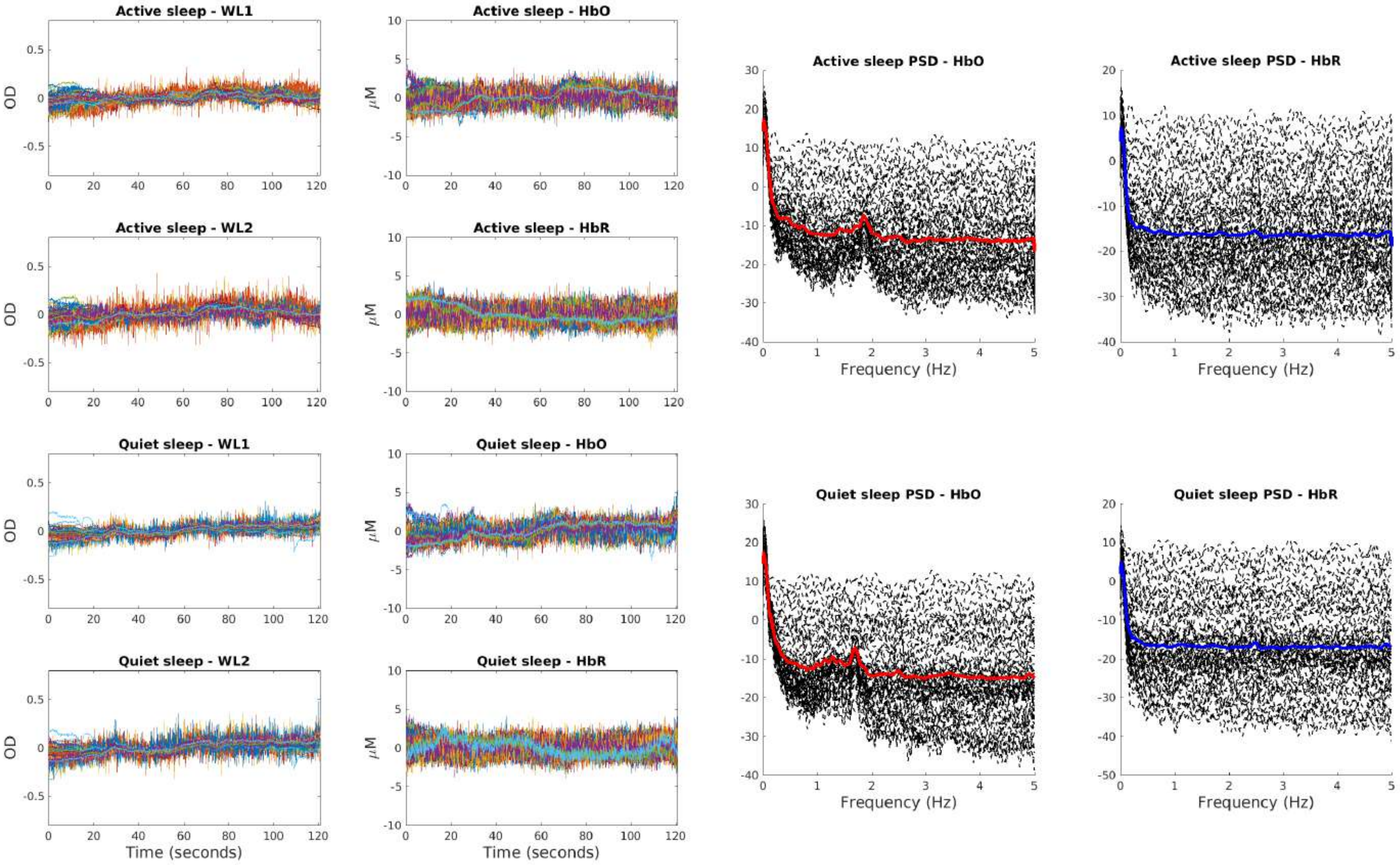

Active sleep - WL1
Active sleep - HbO
Active sleep PSD - HbO
Active sleep PSD - HbR
Active sleep - WL2
Active sleep - HbR
Quiet sleep - WL1
Quiet sleep - HbO
Quiet sleep PSD - HbO
Quiet sleep PSD - HbR
Quiet sleep - WL2
Quiet sleep - HbR
OD
μM
Time (seconds)
Frequency (Hz)

HT_003

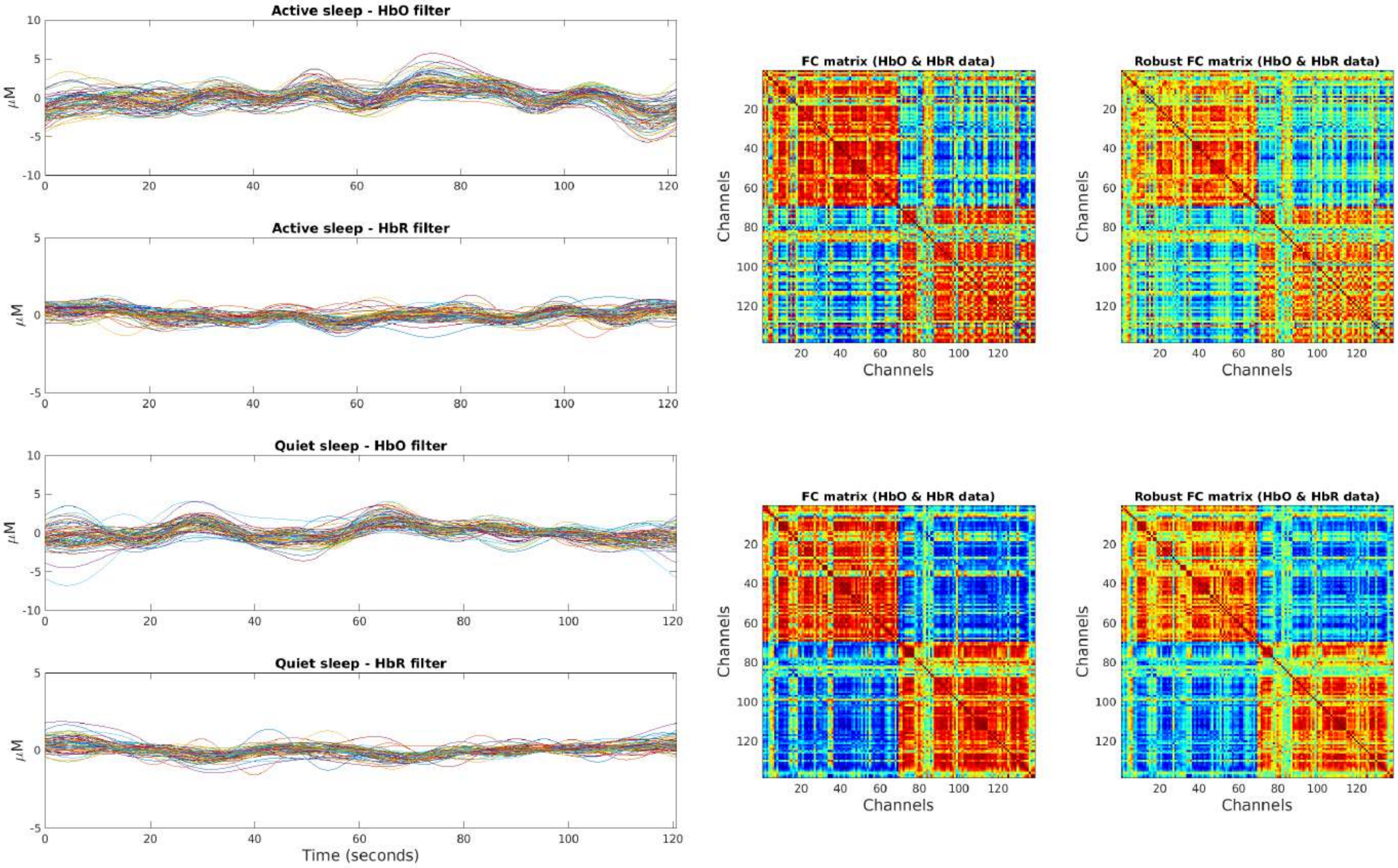
Active sleep - HbO filter
Active sleep - HbR filter
Quiet sleep - HbO filter
Quiet sleep - HbR filter
μM
Time (seconds)
FC matrix (HbO & HbR data)
Robust FC matrix (HbO & HbR data)
FC matrix (HbO & HbR data)
Robust FC matrix (HbO & HbR data)
Channels
Channels

HT_003

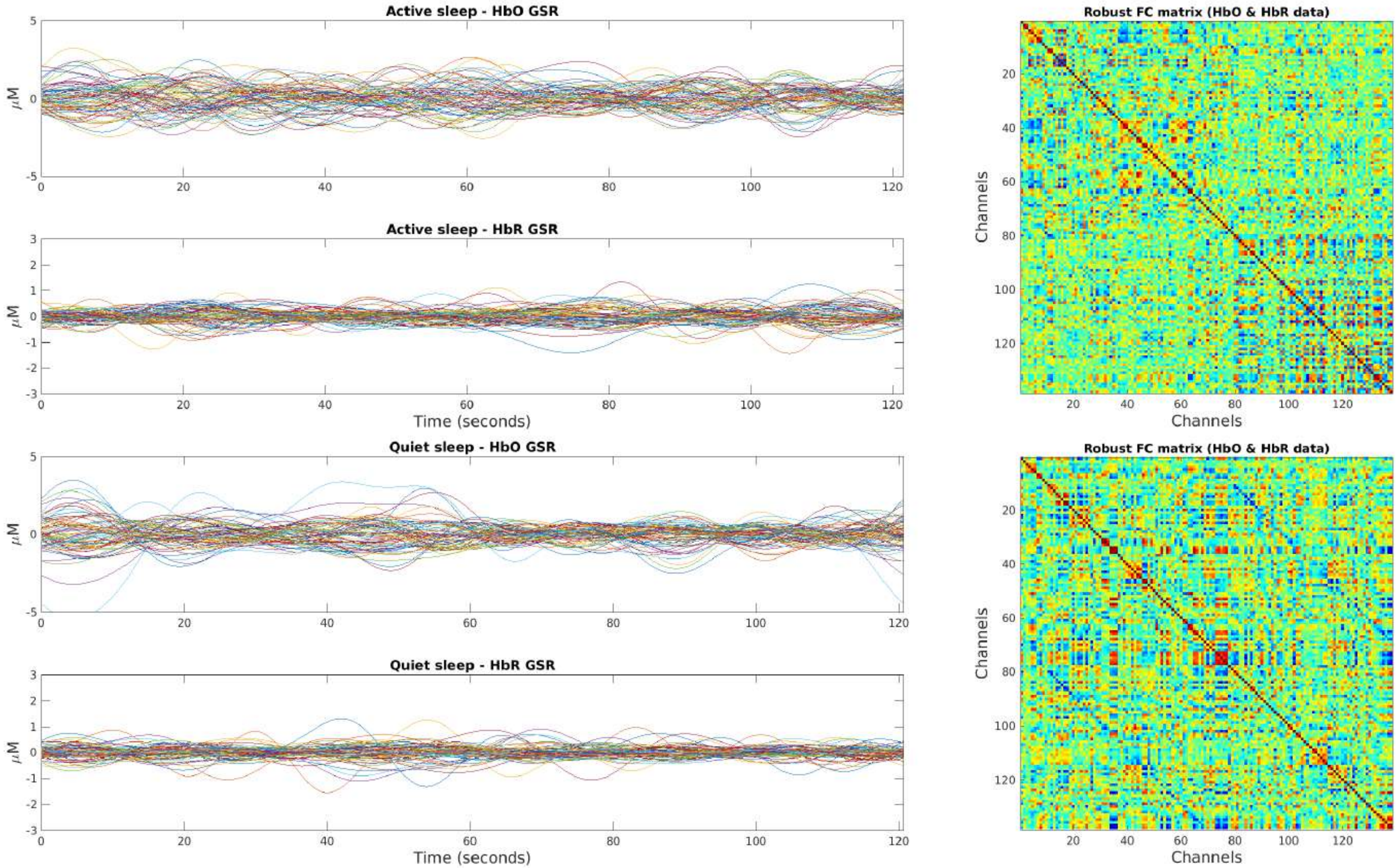
Active sleep - HbO GSR
Active sleep - HbR GSR
Quiet sleep - HbO GSR
Quiet sleep - HbR GSR
μM
Time (seconds)
Robust FC matrix (HbO & HbR data)
Channels

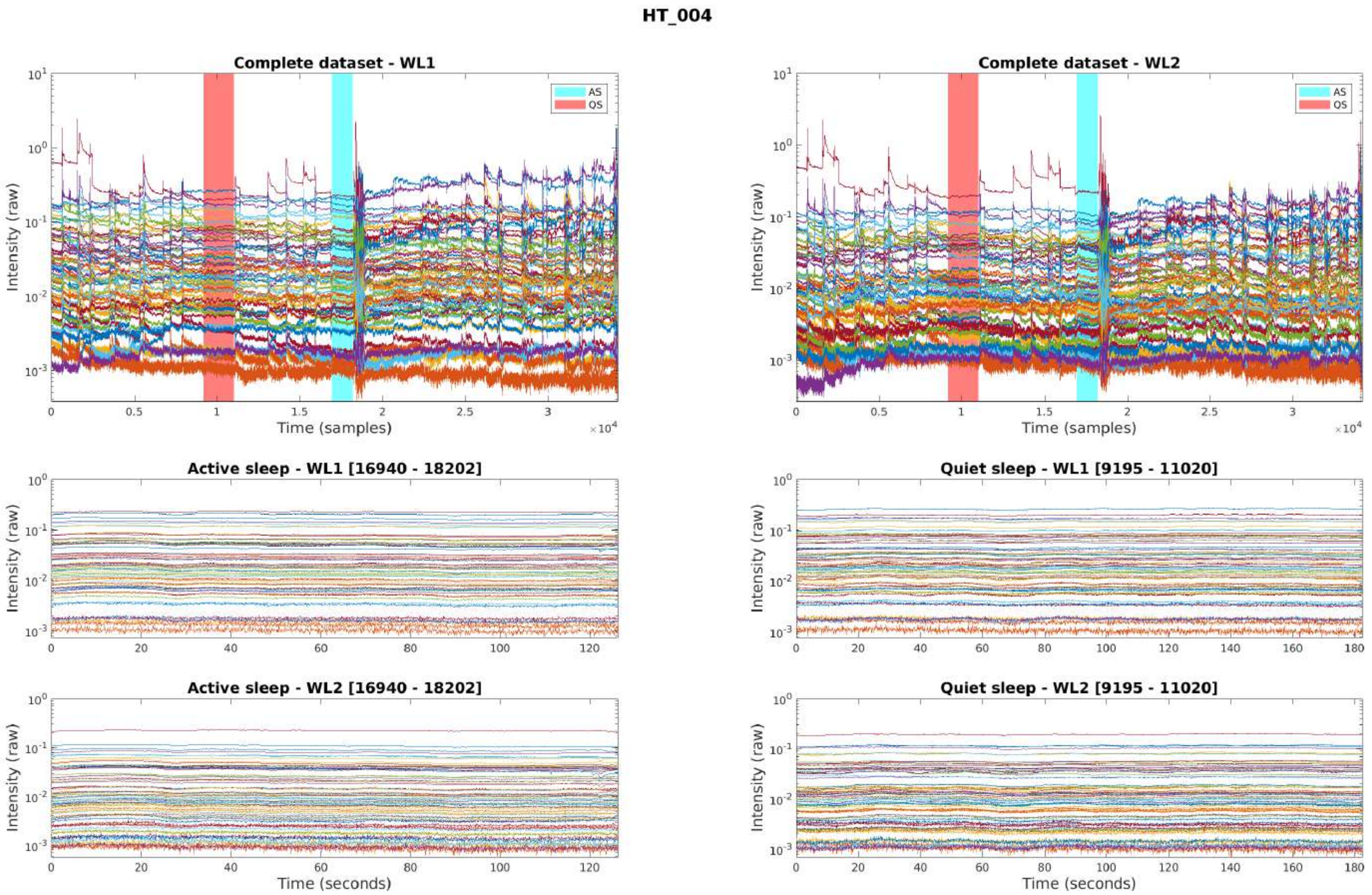
HT_004
Complete dataset - WL1
Complete dataset - WL2
AS
QS
Intensity (raw)
Time (samples)
Active sleep - WL1 [16940 - 18202]
Quiet sleep - WL1 [9195 - 11020]
Active sleep - WL2 [16940 - 18202]
Quiet sleep - WL2 [9195 - 11020]
Time (seconds)

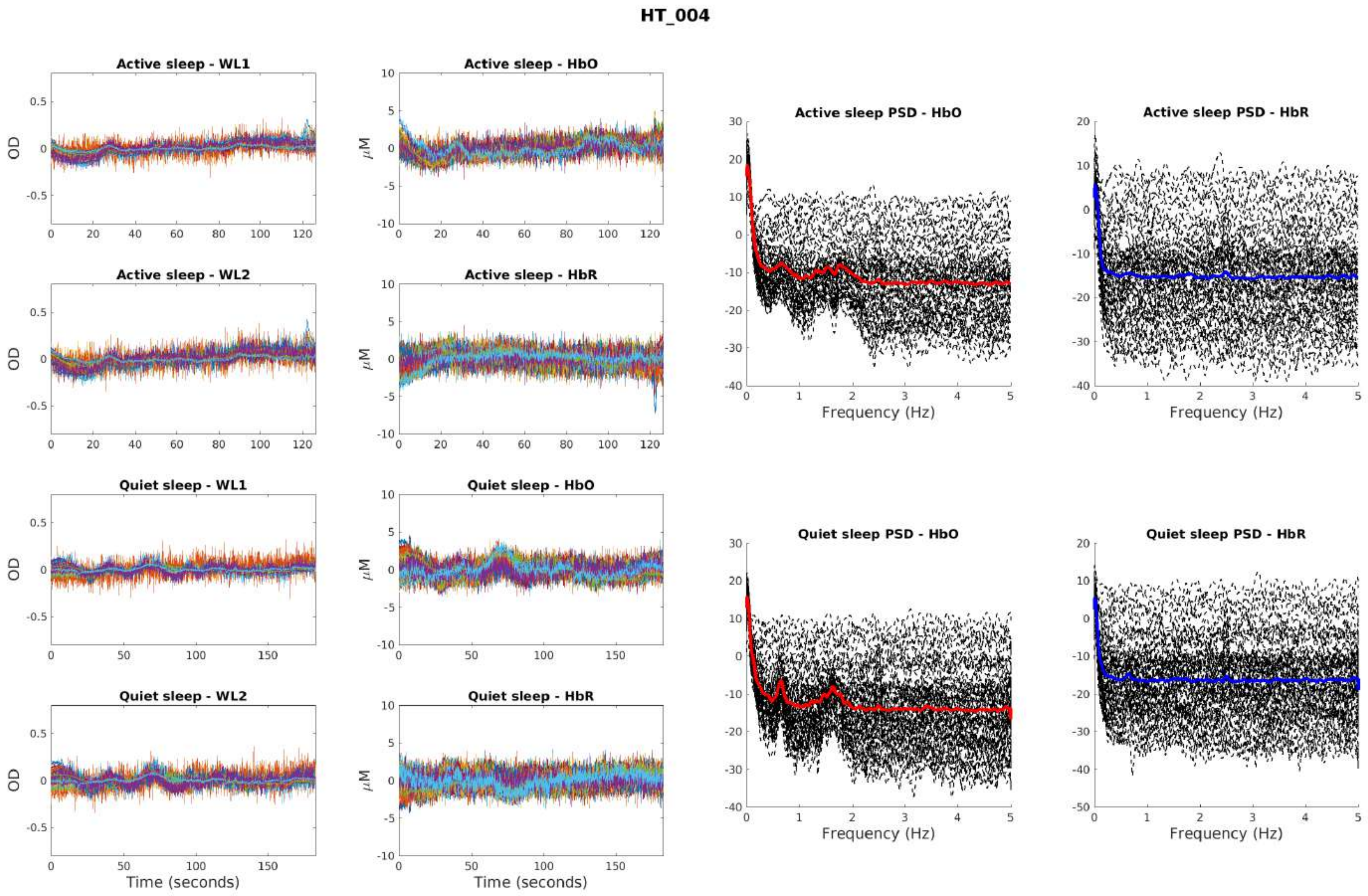

HT_004
Active sleep - WL1
Active sleep - HbO
Active sleep PSD - HbO
Active sleep PSD - HbR
Active sleep - WL2
Active sleep - HbR
Quiet sleep - WL1
Quiet sleep - HbO
Quiet sleep PSD - HbO
Quiet sleep PSD - HbR
Quiet sleep - WL2
Quiet sleep - HbR
OD
μM
Time (seconds)
Frequency (Hz)

HT_004

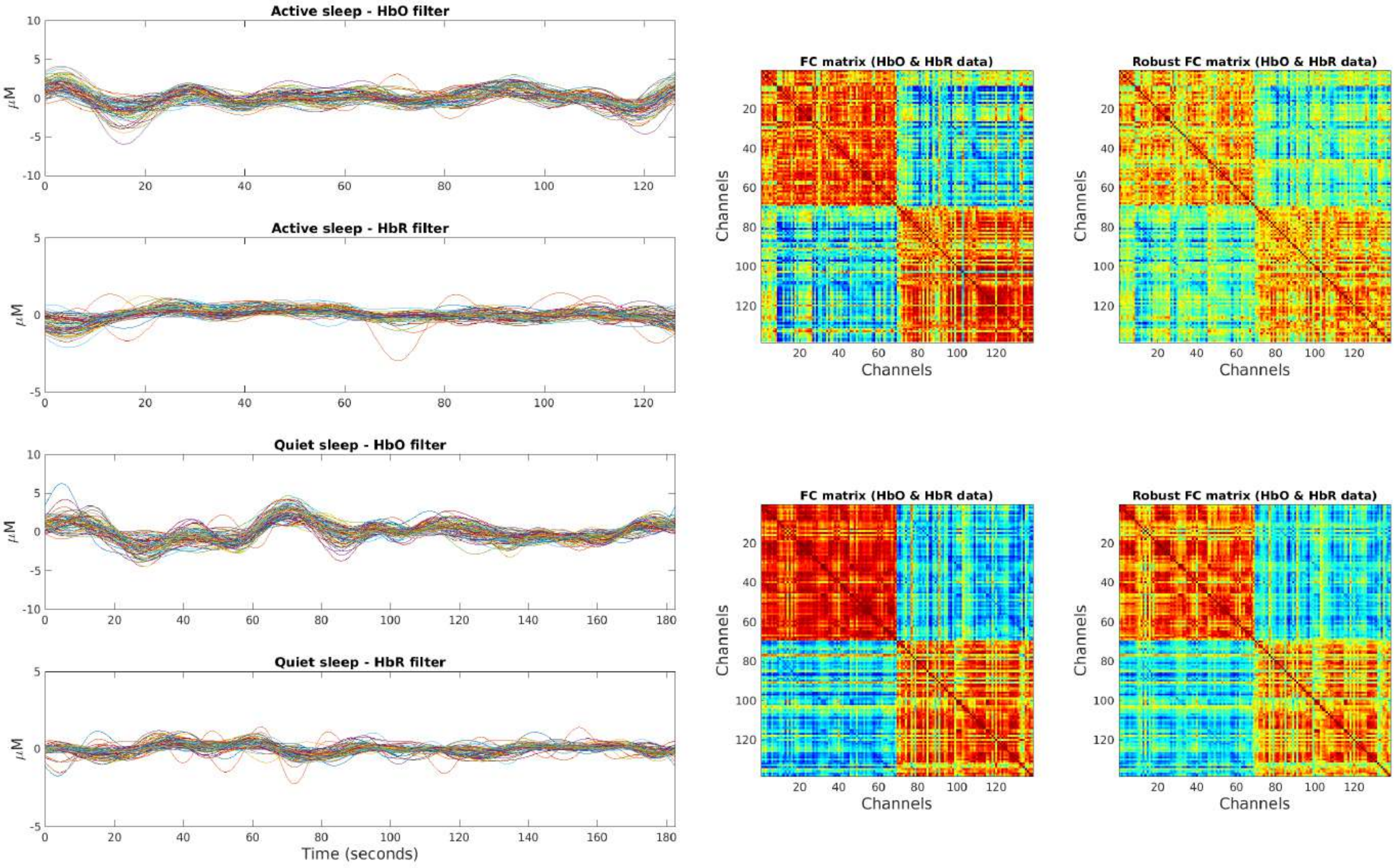
Active sleep - HbO filter
Active sleep - HbR filter
Quiet sleep - HbO filter
Quiet sleep - HbR filter
μM
Time (seconds)
FC matrix (HbO & HbR data)
Robust FC matrix (HbO & HbR data)
Channels

HT_004

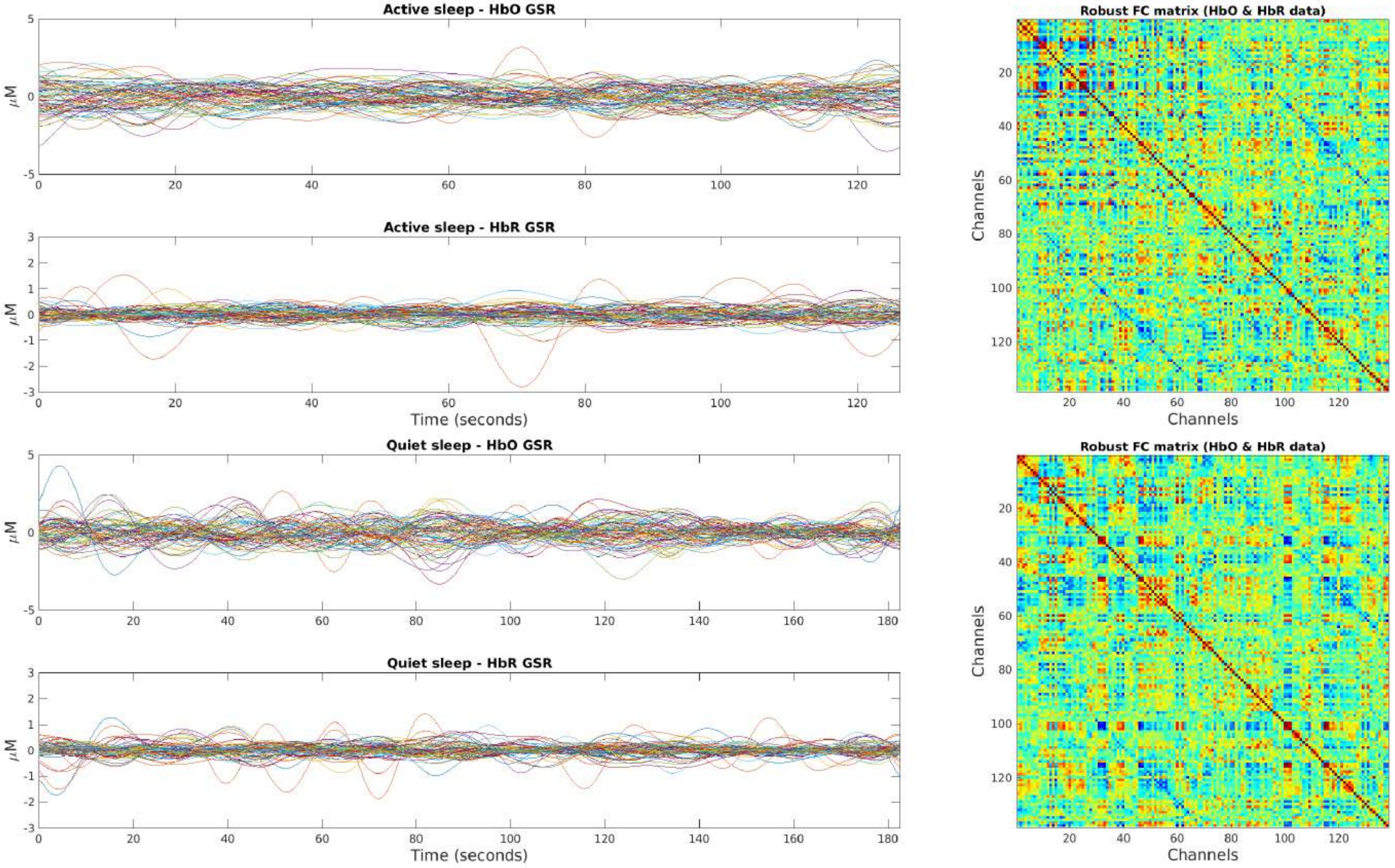
Active sleep - HbO GSR
Active sleep - HbR GSR
Quiet sleep - HbO GSR
Quiet sleep - HbR GSR
μM
Time (seconds)
Robust FC matrix (HbO & HbR data)
Robust FC matrix (HbO & HbR data)
Channels
Channels

HT_006

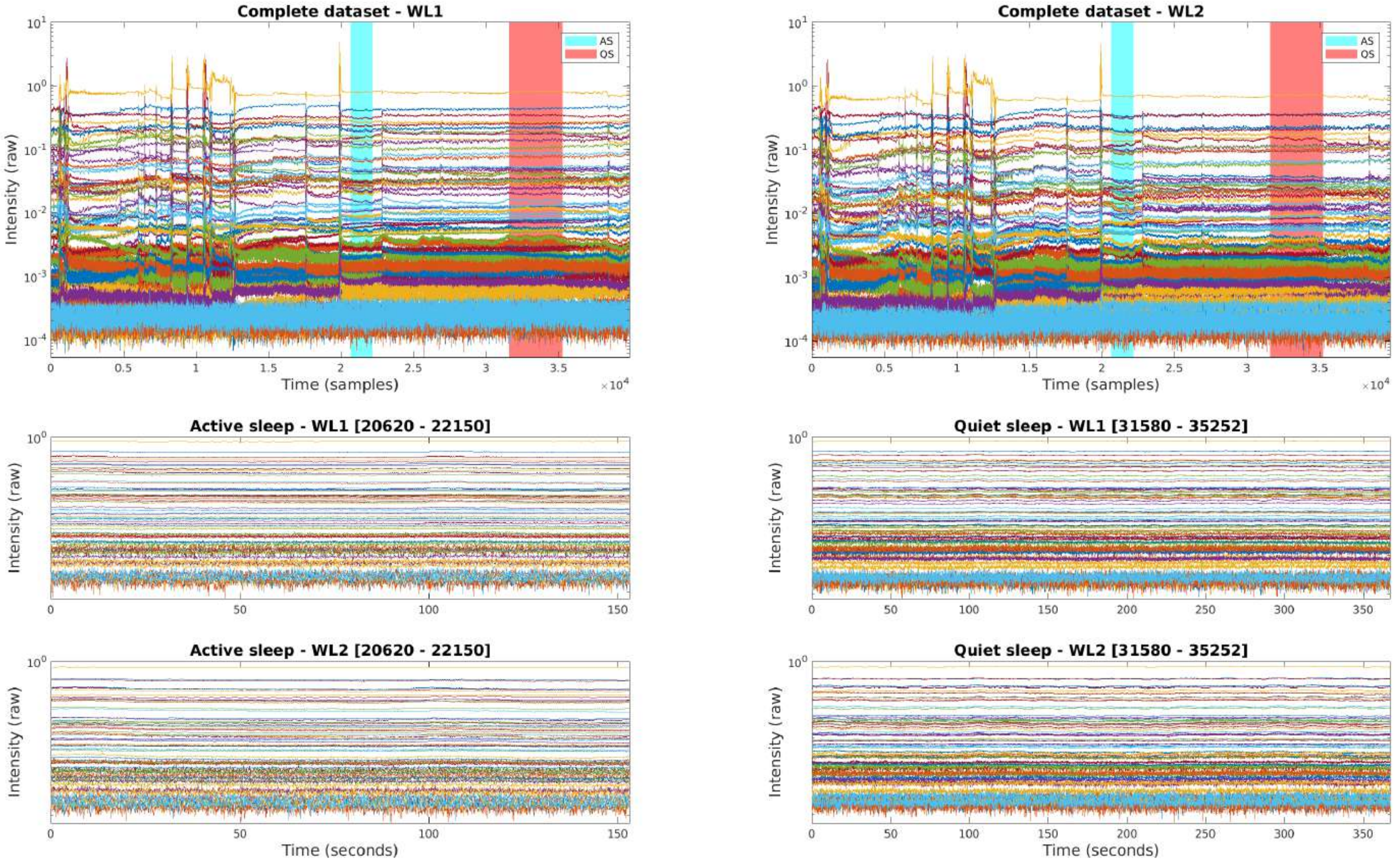

Complete dataset - WL1
Complete dataset - WL2
AS
QS
Intensity (raw)
Time (samples)
$\times 10^4$
0
0.5
1
1.5
2
2.5
3
3.5
10^{1}
10^{0}
10^{-1}
10^{-2}
10^{-3}
10^{-4}
Active sleep - WL1 [20620 - 22150]
Quiet sleep - WL1 [31580 - 35252]
Active sleep - WL2 [20620 - 22150]
Quiet sleep - WL2 [31580 - 35252]
Time (seconds)
50
100
150
200
250
300
350

HT_006

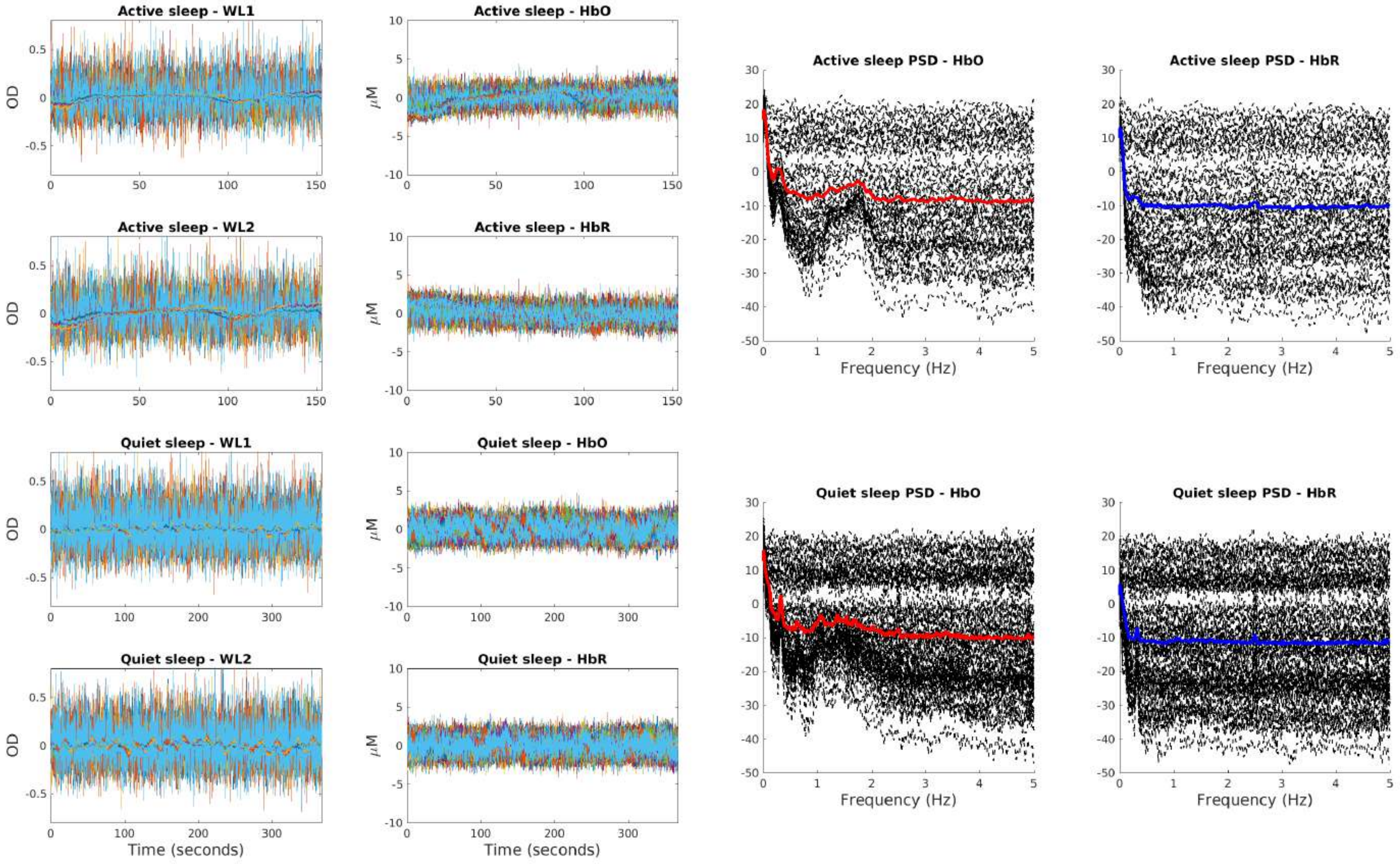
Active sleep - WL1
Active sleep - HbO
Active sleep PSD - HbO
Active sleep PSD - HbR
Active sleep - WL2
Active sleep - HbR
Quiet sleep - WL1
Quiet sleep - HbO
Quiet sleep PSD - HbO
Quiet sleep PSD - HbR
Quiet sleep - WL2
Quiet sleep - HbR
OD
μM
Time (seconds)
Frequency (Hz)

HT_006

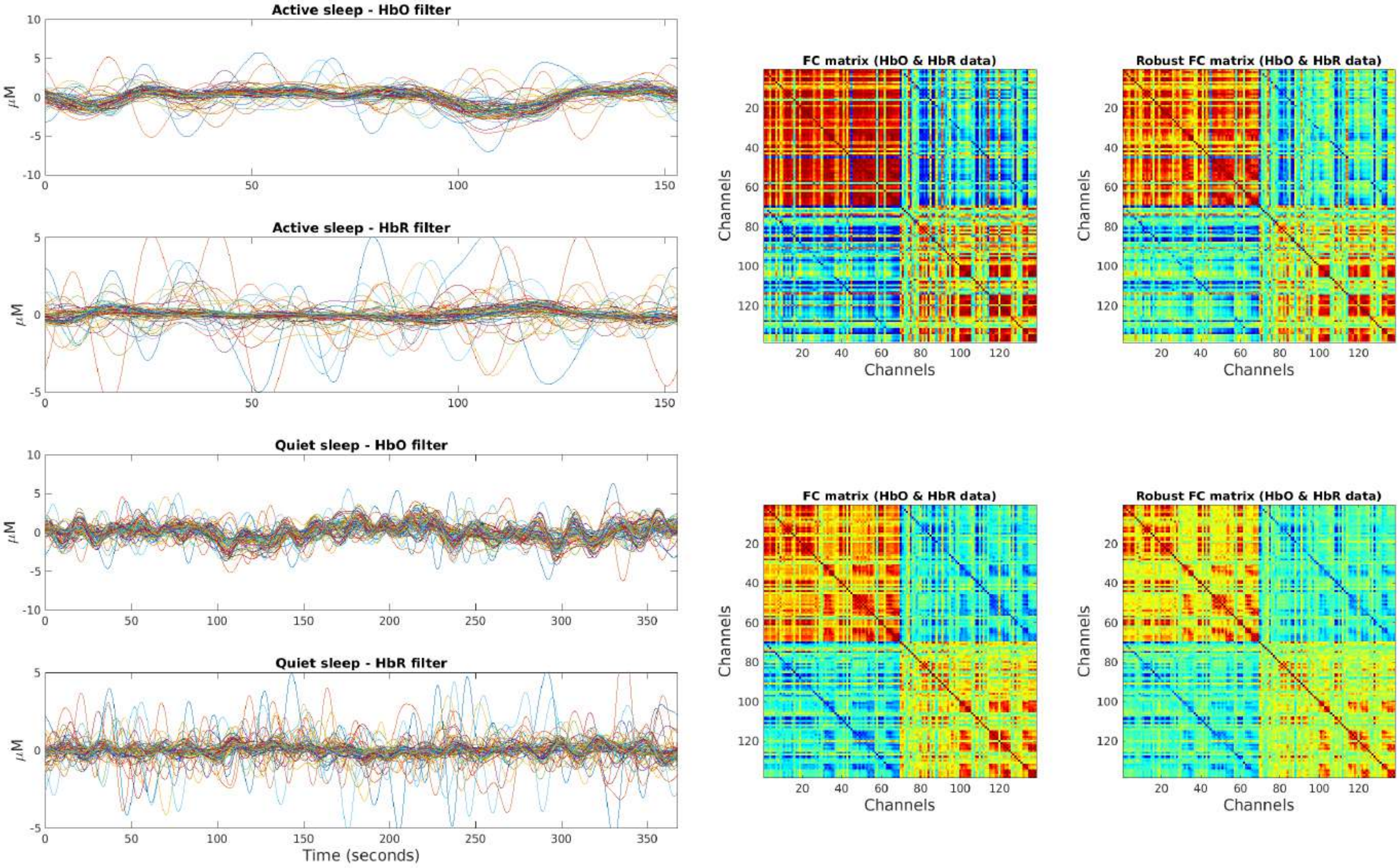

Active sleep - HbO filter
Active sleep - HbR filter
Quiet sleep - HbO filter
Quiet sleep - HbR filter
μM
Time (seconds)
FC matrix (HbO & HbR data)
Robust FC matrix (HbO & HbR data)
Channels

HT_006

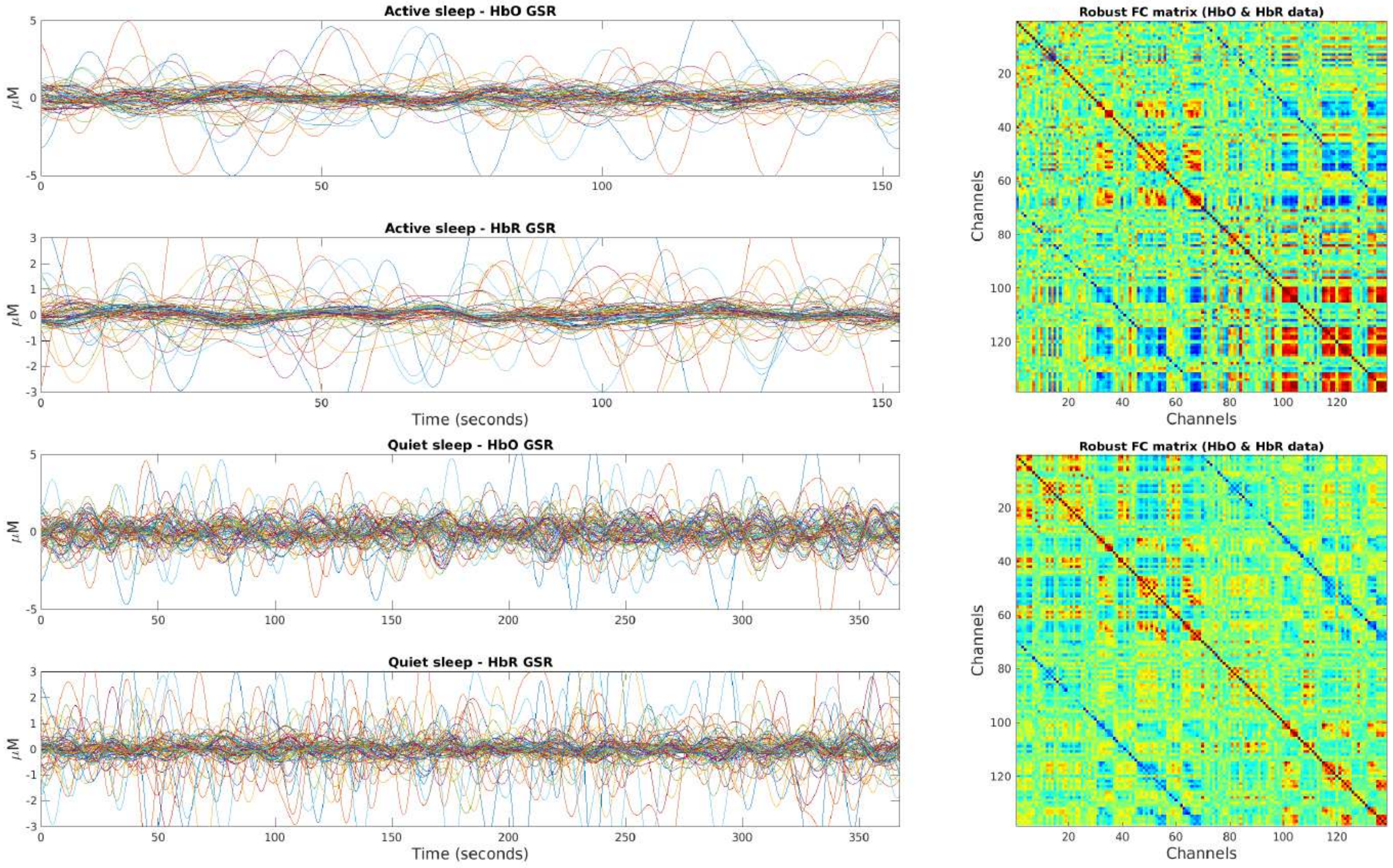

Active sleep - HbO GSR
Active sleep - HbR GSR
Quiet sleep - HbO GSR
Quiet sleep - HbR GSR
μM
Time (seconds)
Robust FC matrix (HbO & HbR data)
Channels

HT_007

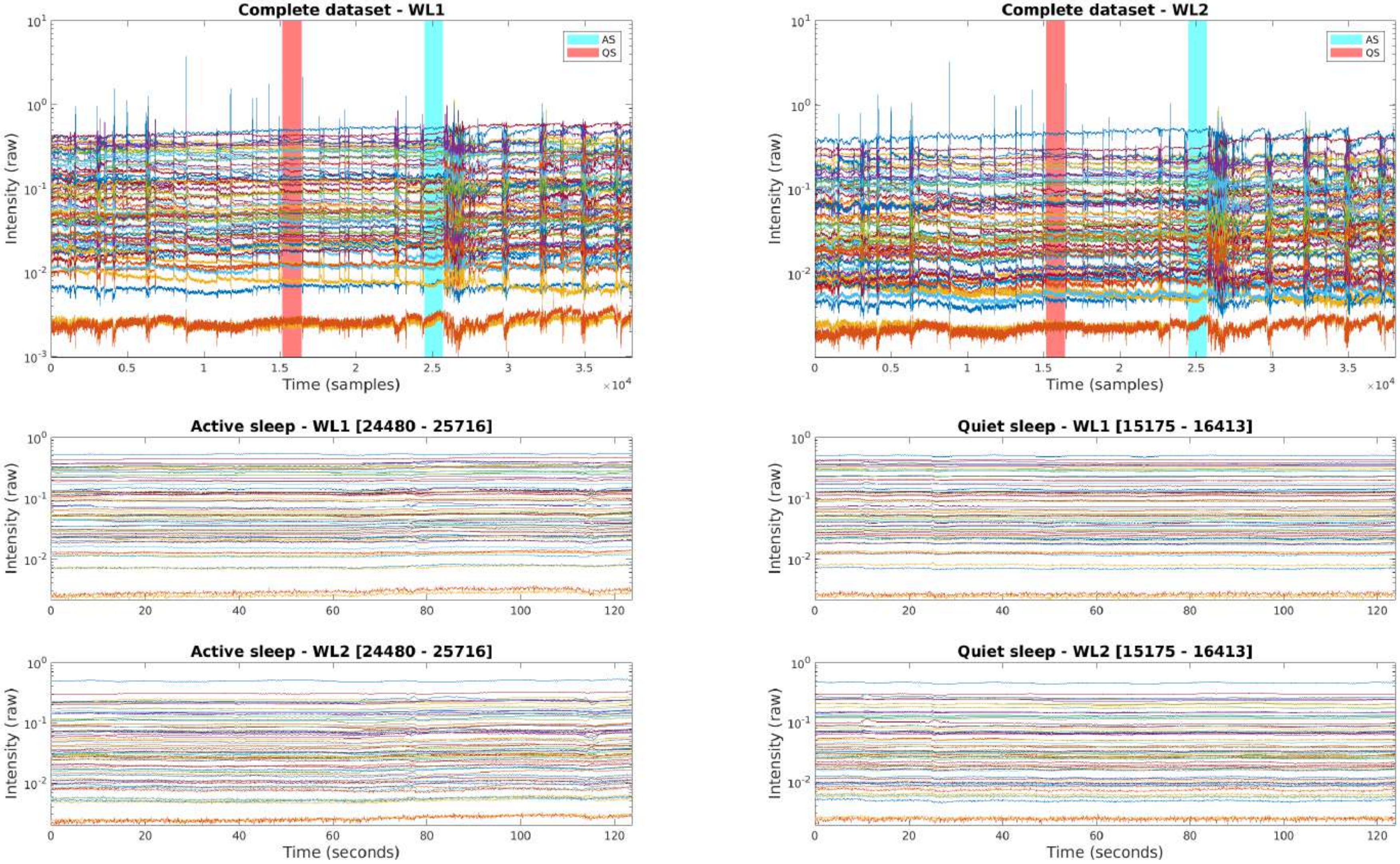

Complete dataset - WL1
Complete dataset - WL2
AS
QS
Intensity (raw)
Time (samples)
×10^4
Active sleep - WL1 [24480 - 25716]
Quiet sleep - WL1 [15175 - 16413]
Active sleep - WL2 [24480 - 25716]
Quiet sleep - WL2 [15175 - 16413]
Time (seconds)

HT_007

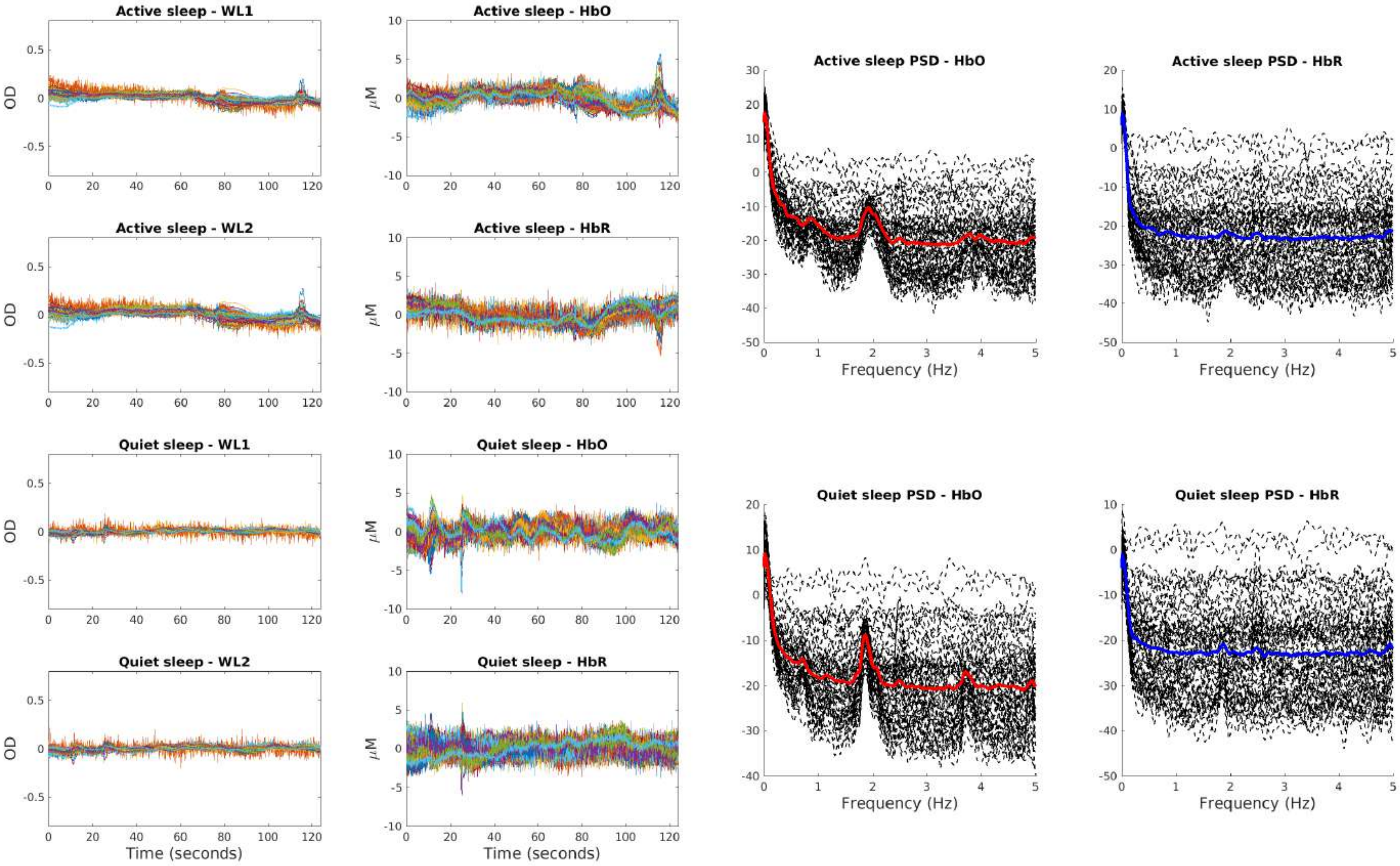
Active sleep - WL1
Active sleep - WL2
Quiet sleep - WL1
Quiet sleep - WL2
Active sleep - HbO
Active sleep - HbR
Quiet sleep - HbO
Quiet sleep - HbR
Active sleep PSD - HbO
Active sleep PSD - HbR
Quiet sleep PSD - HbO
Quiet sleep PSD - HbR
OD
μM
Time (seconds)
Frequency (Hz)

HT_007

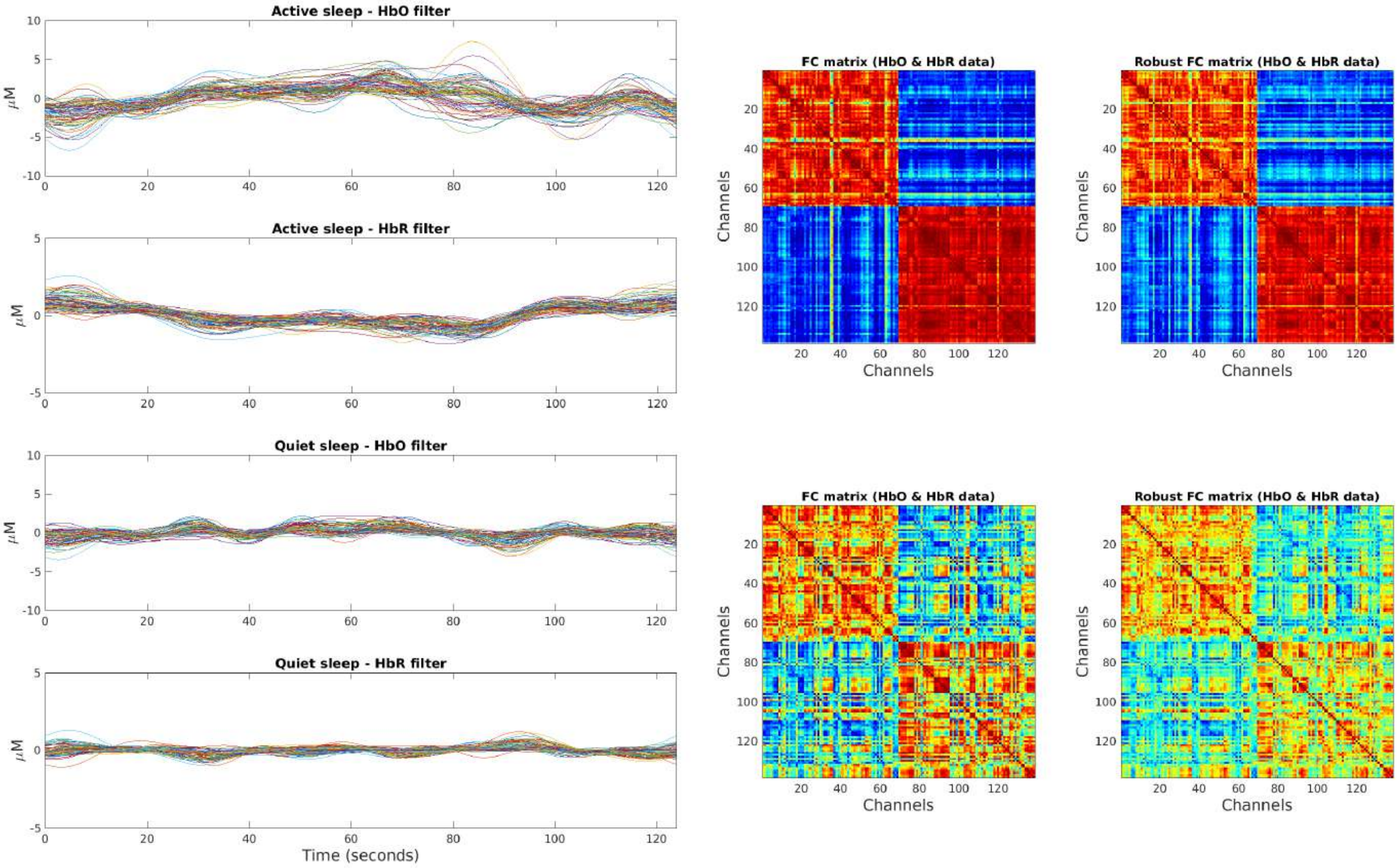

Active sleep - HbO filter
Active sleep - HbR filter
Quiet sleep - HbO filter
Quiet sleep - HbR filter
μM
Time (seconds)
FC matrix (HbO & HbR data)
Robust FC matrix (HbO & HbR data)
Channels

HT_007

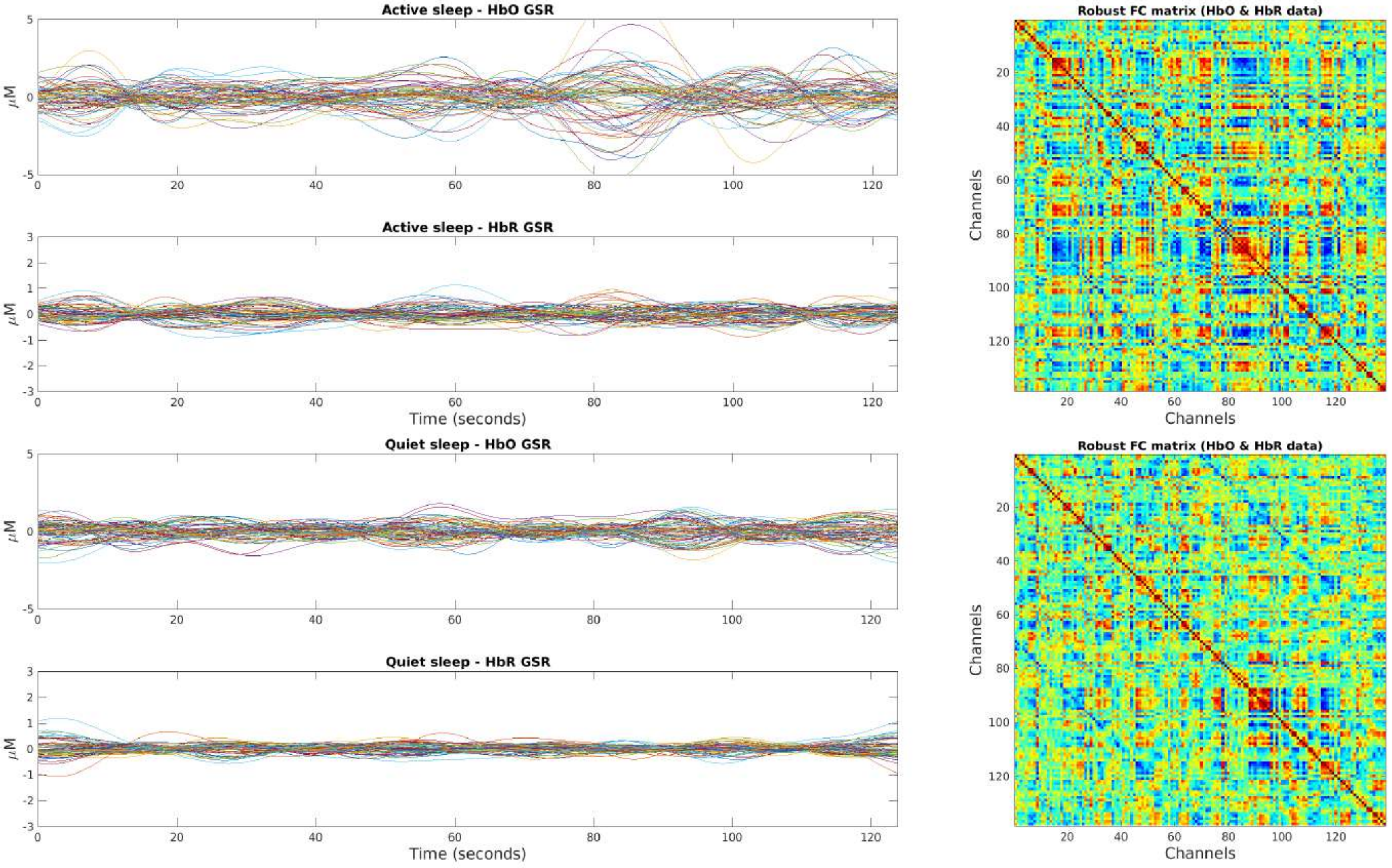
Active sleep - HbO GSR
Active sleep - HbR GSR
Quiet sleep - HbO GSR
Quiet sleep - HbR GSR
μM
Time (seconds)
Robust FC matrix (HbO & HbR data)
Robust FC matrix (HbO & HbR data)
Channels
Channels

HT_008

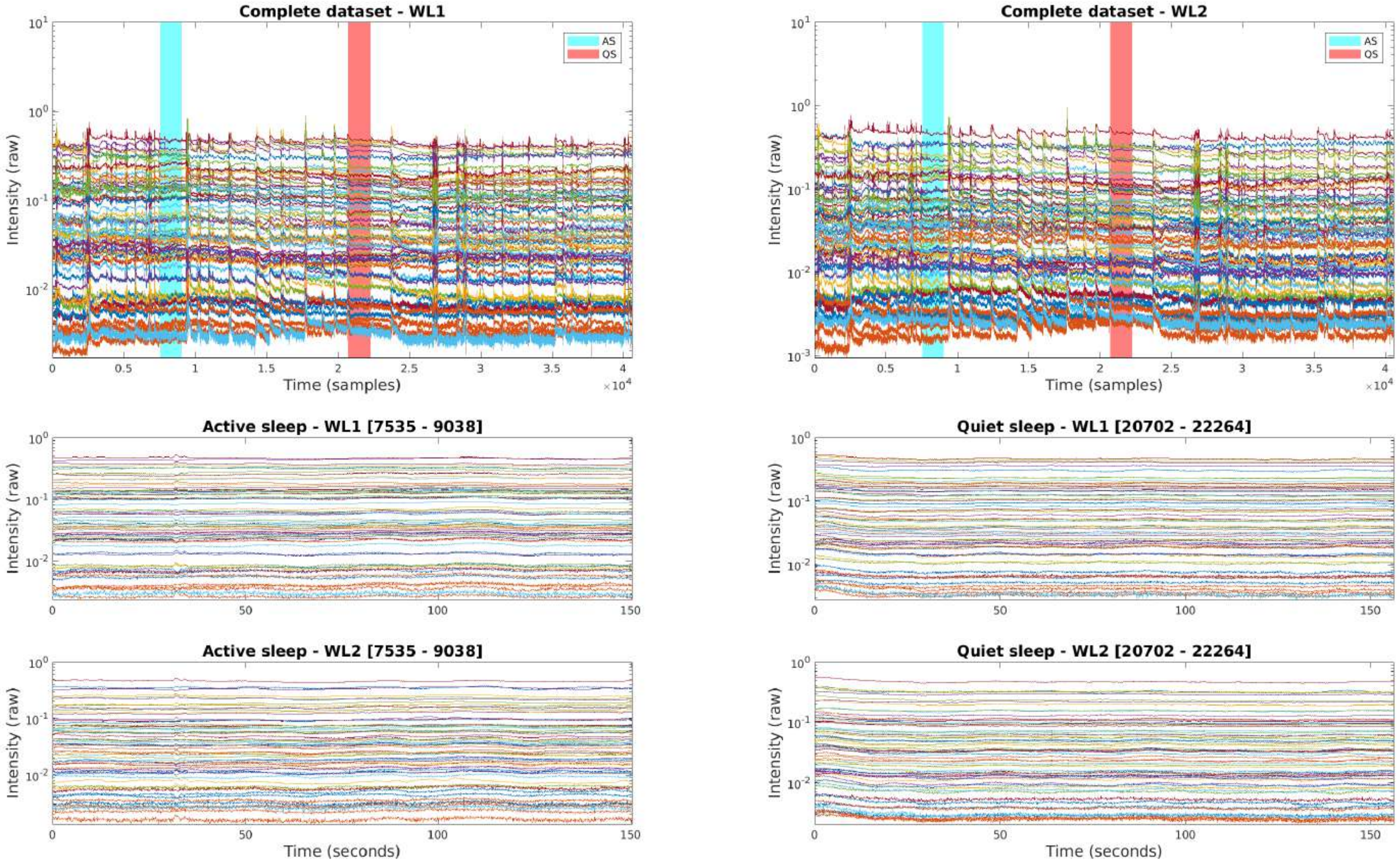
Complete dataset - WL1
Complete dataset - WL2
AS
QS
Intensity (raw)
Time (samples)
×10^4
Active sleep - WL1 [7535 - 9038]
Quiet sleep - WL1 [20702 - 22264]
Active sleep - WL2 [7535 - 9038]
Quiet sleep - WL2 [20702 - 22264]
Time (seconds)

HT_008

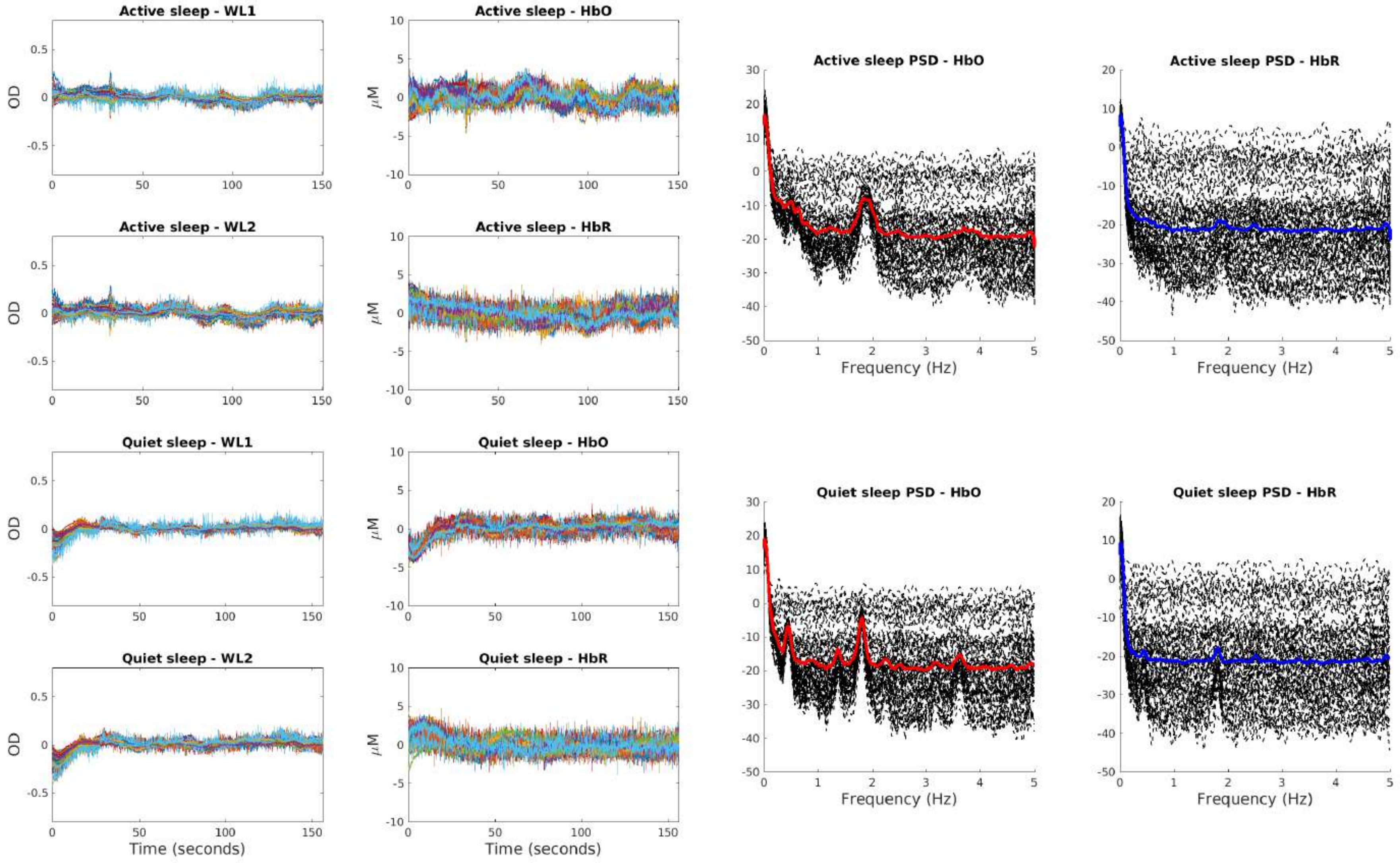

Active sleep - WL1
Active sleep - HbO
Active sleep PSD - HbO
Active sleep PSD - HbR
Active sleep - WL2
Active sleep - HbR
Quiet sleep - WL1
Quiet sleep - HbO
Quiet sleep PSD - HbO
Quiet sleep PSD - HbR
Quiet sleep - WL2
Quiet sleep - HbR
OD
μM
Time (seconds)
Frequency (Hz)

HT_008

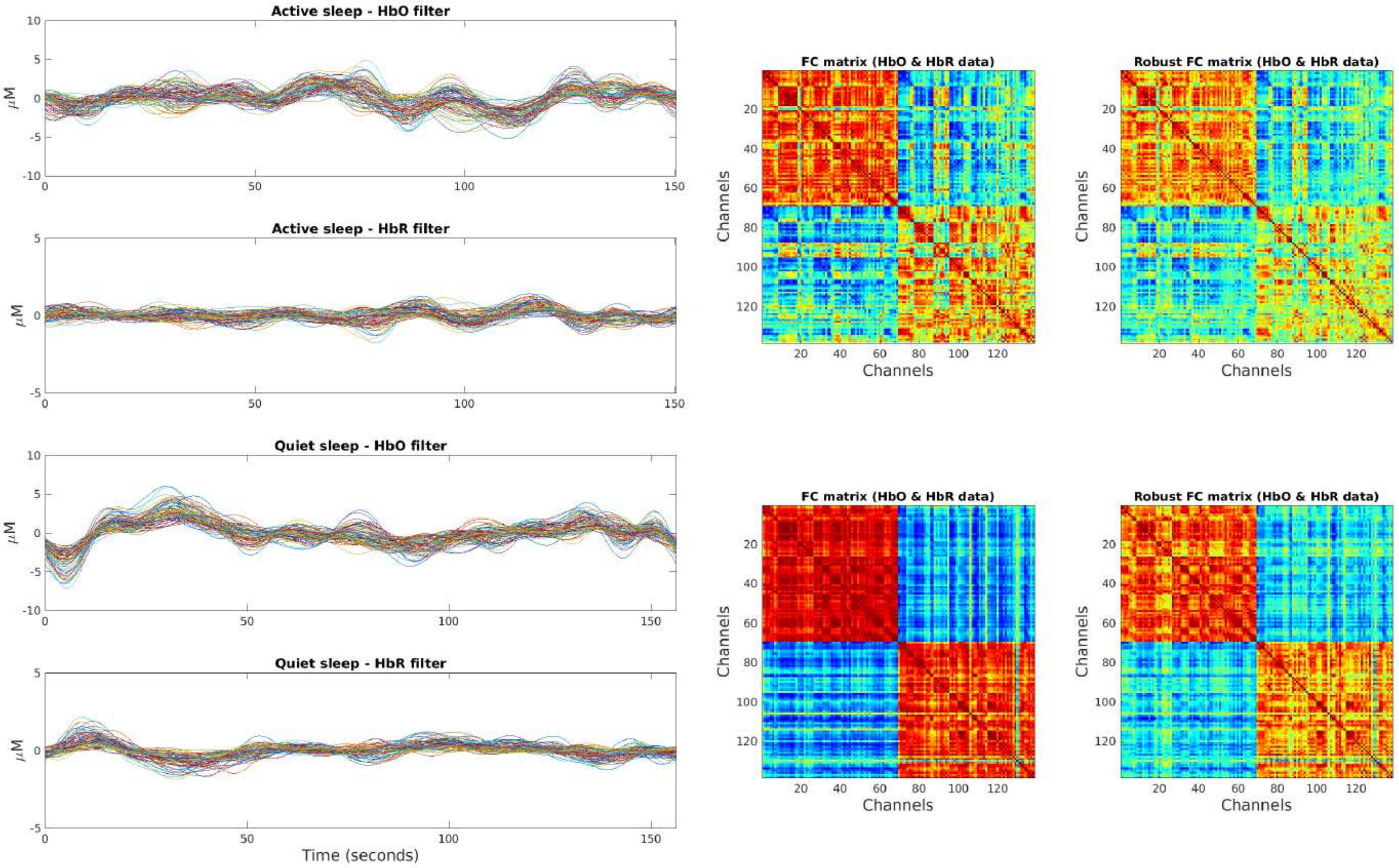
Active sleep - HbO filter
Active sleep - HbR filter
Quiet sleep - HbO filter
Quiet sleep - HbR filter
μM
Time (seconds)
FC matrix (HbO & HbR data)
Robust FC matrix (HbO & HbR data)
Channels

HT_008

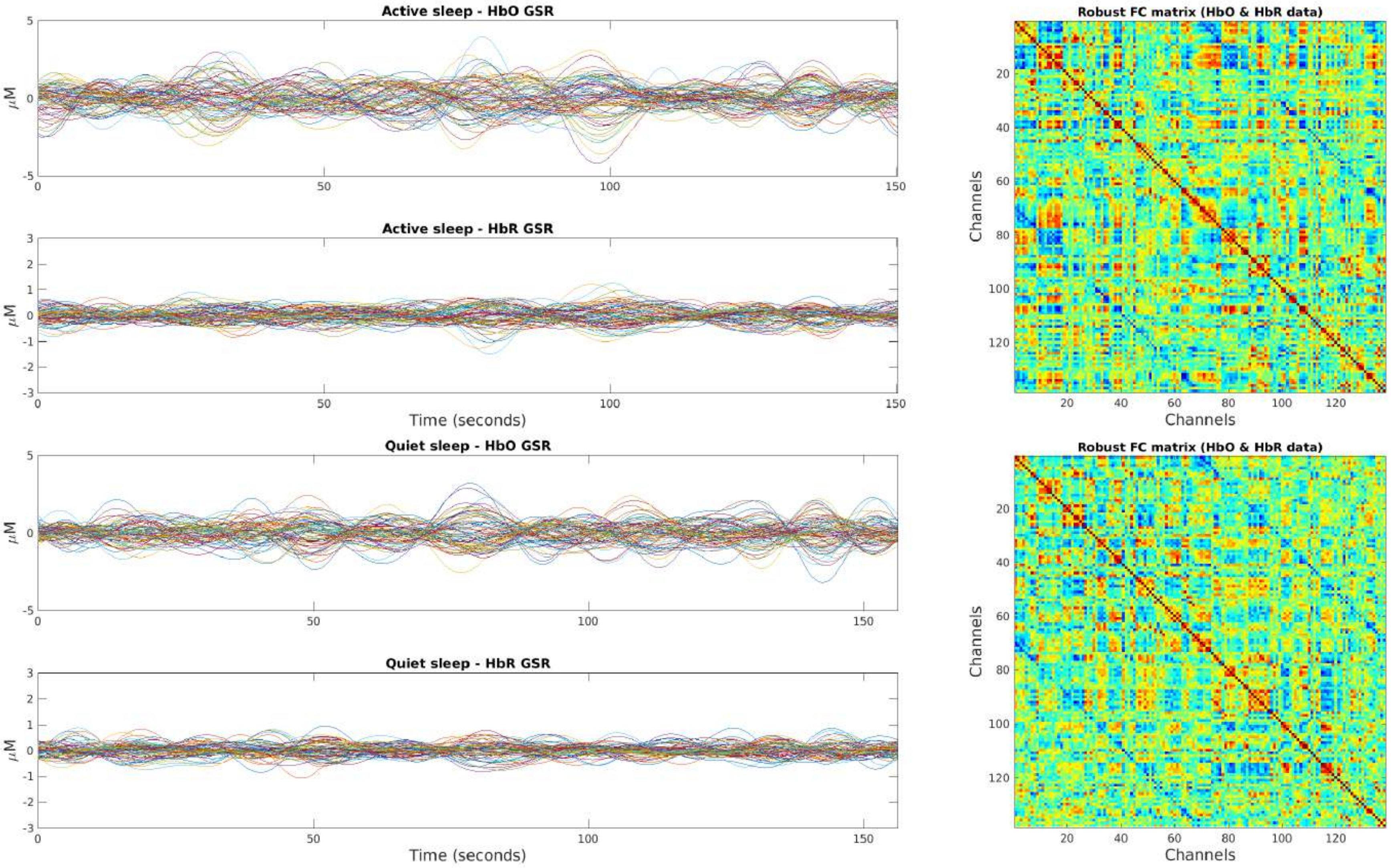

Active sleep - HbO GSR
Active sleep - HbR GSR
Quiet sleep - HbO GSR
Quiet sleep - HbR GSR
μM
Time (seconds)
Robust FC matrix (HbO & HbR data)
Channels

HT_009

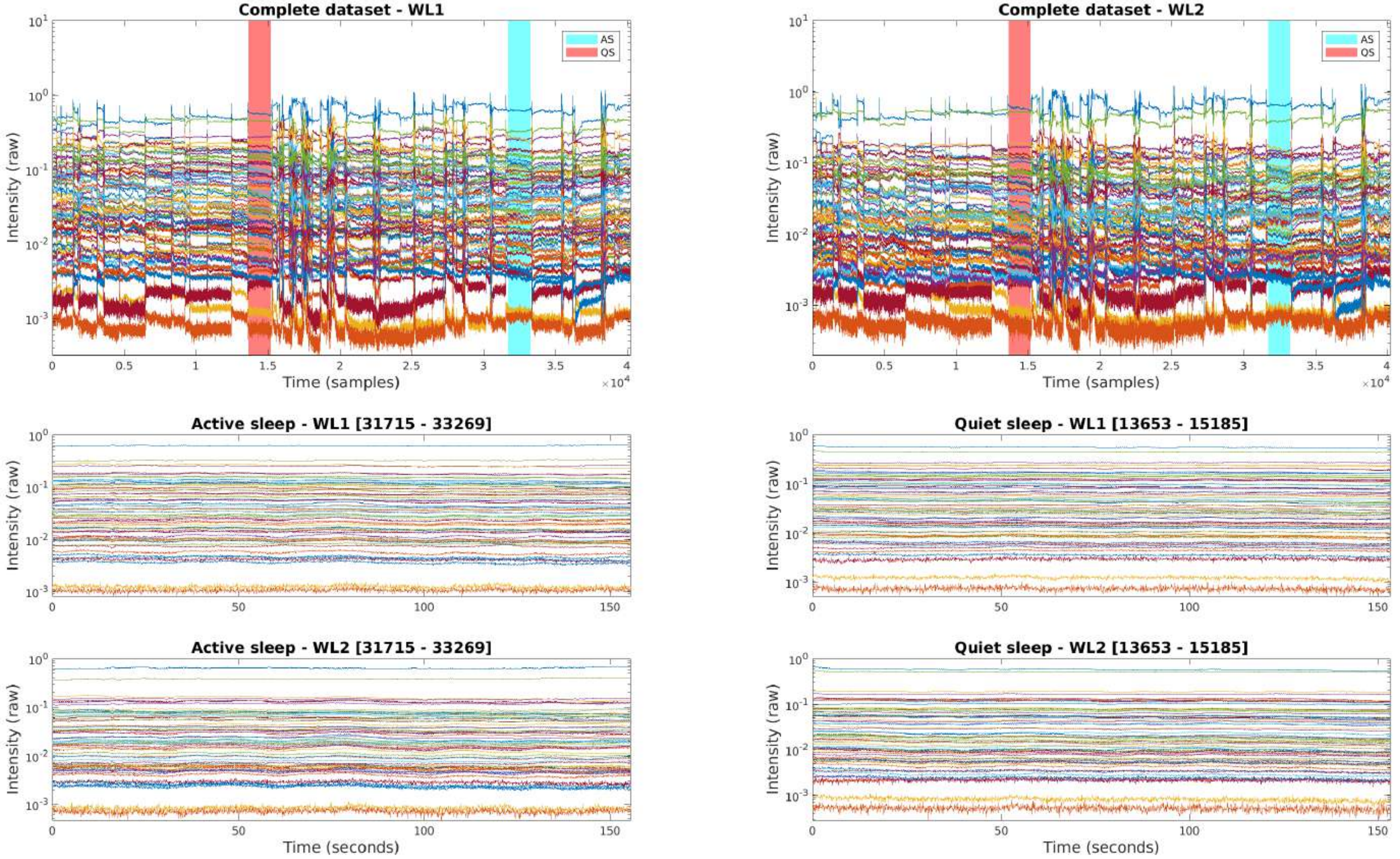
Complete dataset - WL1
Complete dataset - WL2
AS
QS
Intensity (raw)
Time (samples)
×10^4
Active sleep - WL1 [31715 - 33269]
Quiet sleep - WL1 [13653 - 15185]
Active sleep - WL2 [31715 - 33269]
Quiet sleep - WL2 [13653 - 15185]
Time (seconds)

HT_009

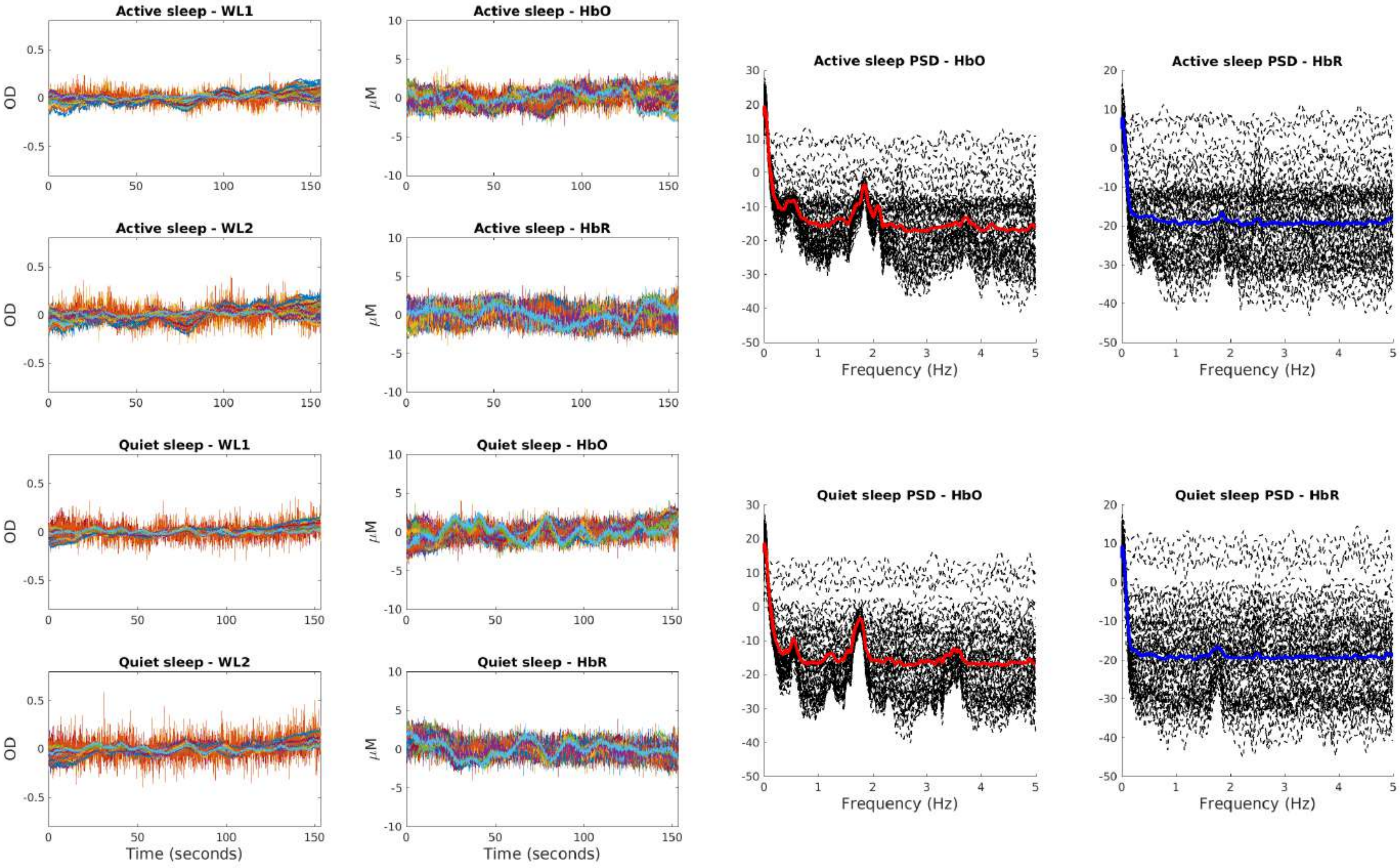
Active sleep - WL1
Active sleep - HbO
Active sleep PSD - HbO
Active sleep PSD - HbR
Active sleep - WL2
Active sleep - HbR
Quiet sleep - WL1
Quiet sleep - HbO
Quiet sleep PSD - HbO
Quiet sleep PSD - HbR
Quiet sleep - WL2
Quiet sleep - HbR
OD
μM
Time (seconds)
Frequency (Hz)

HT_009

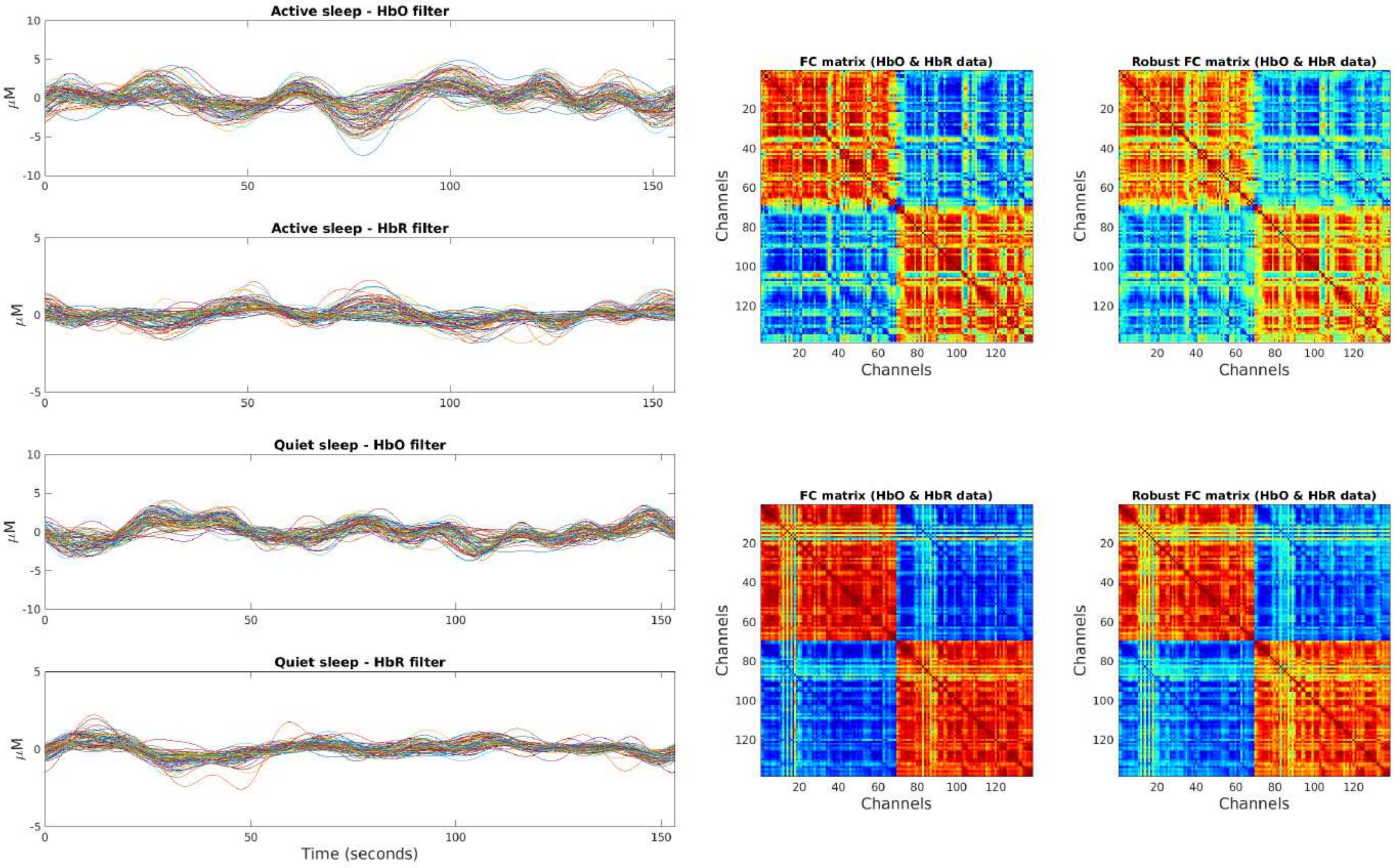

Active sleep - HbO filter
Active sleep - HbR filter
Quiet sleep - HbO filter
Quiet sleep - HbR filter
μM
Time (seconds)
FC matrix (HbO & HbR data)
Robust FC matrix (HbO & HbR data)
Channels

HT_009

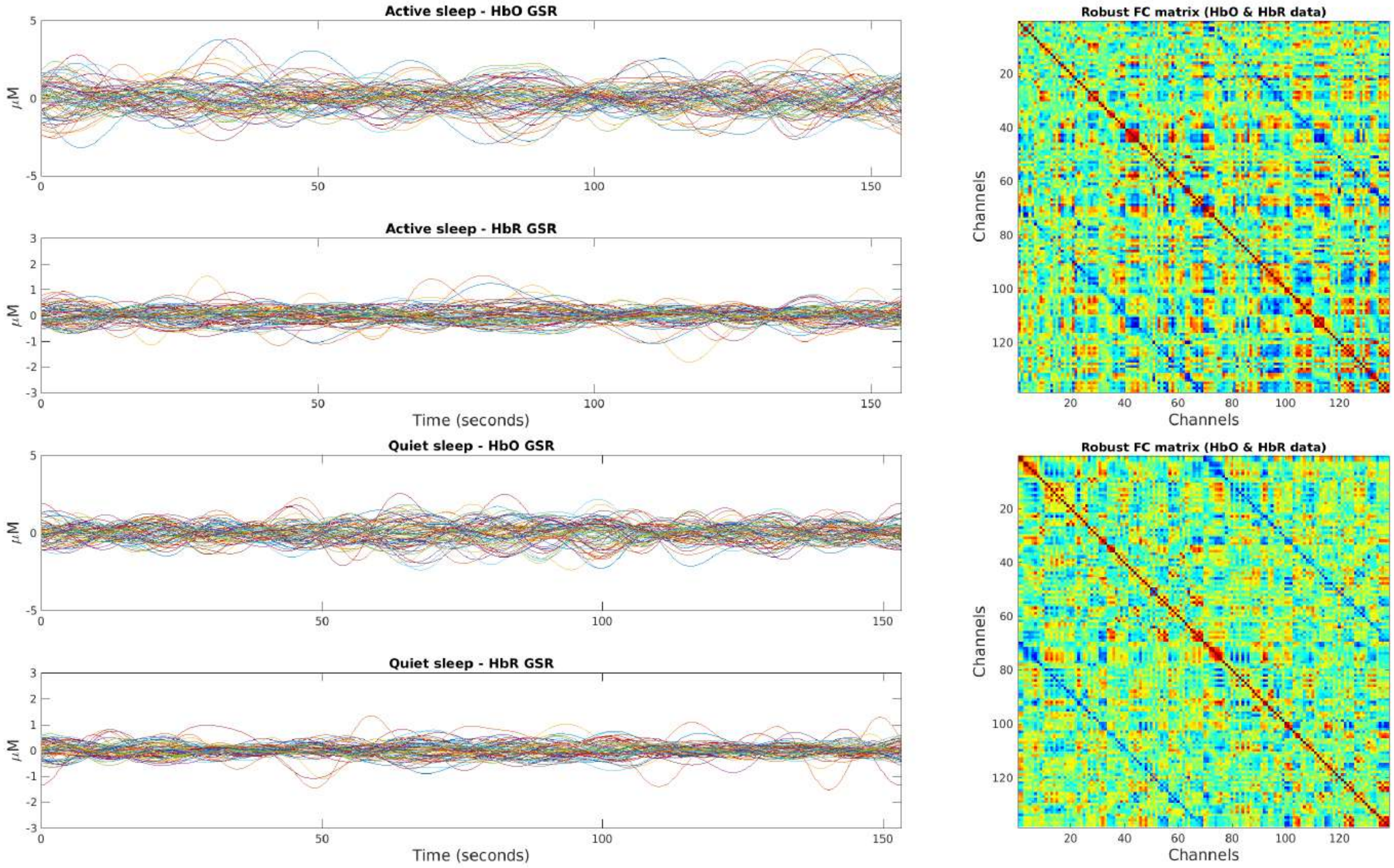
Active sleep - HbO GSR
Active sleep - HbR GSR
Quiet sleep - HbO GSR
Quiet sleep - HbR GSR
μM
Time (seconds)
Robust FC matrix (HbO & HbR data)
Channels
Channels
Robust FC matrix (HbO & HbR data)
Channels
Channels

HT_012

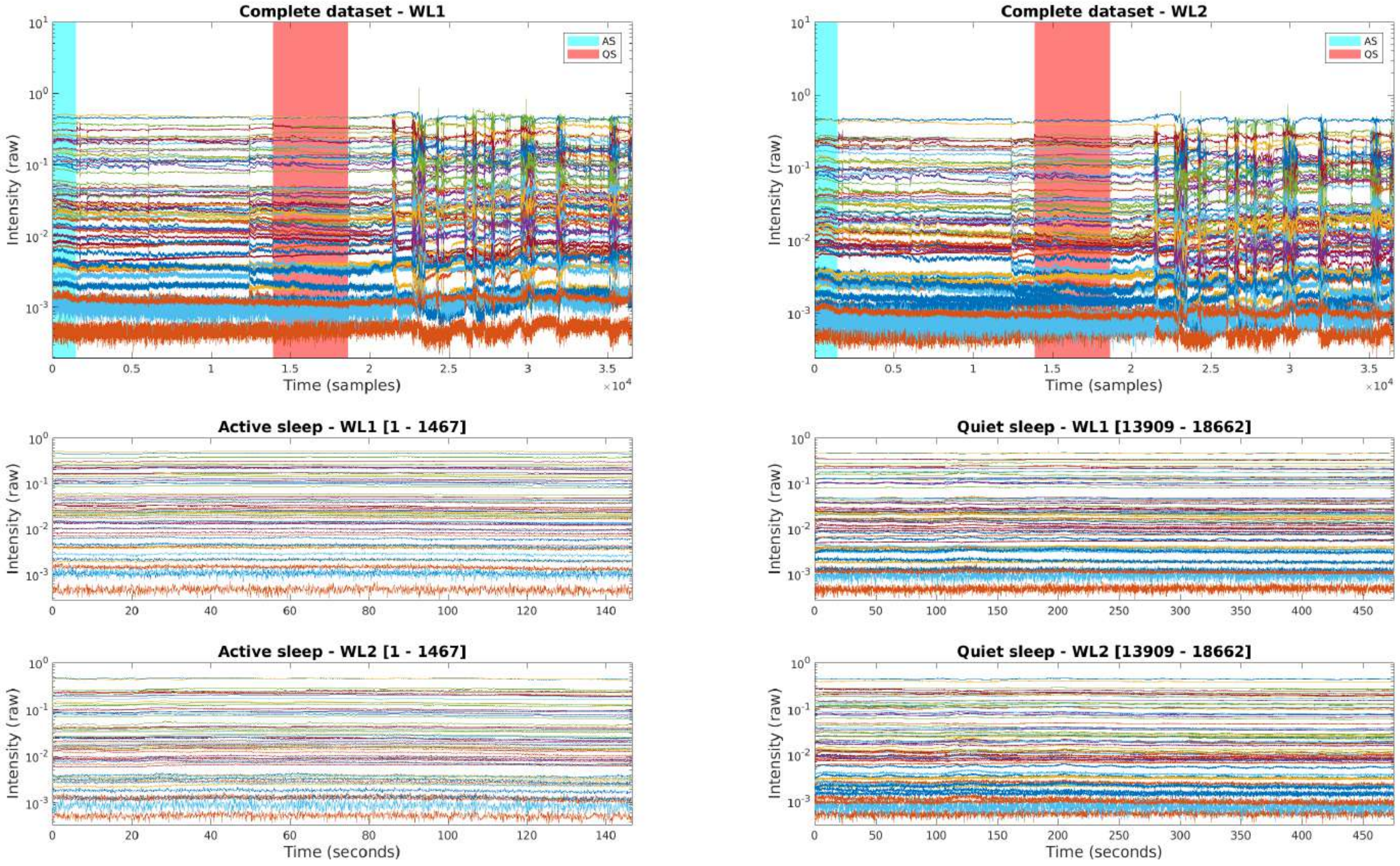
Complete dataset - WL1
Complete dataset - WL2
AS
QS
Intensity (raw)
Time (samples)
Active sleep - WL1 [1 - 1467]
Quiet sleep - WL1 [13909 - 18662]
Active sleep - WL2 [1 - 1467]
Quiet sleep - WL2 [13909 - 18662]
Time (seconds)

HT_012

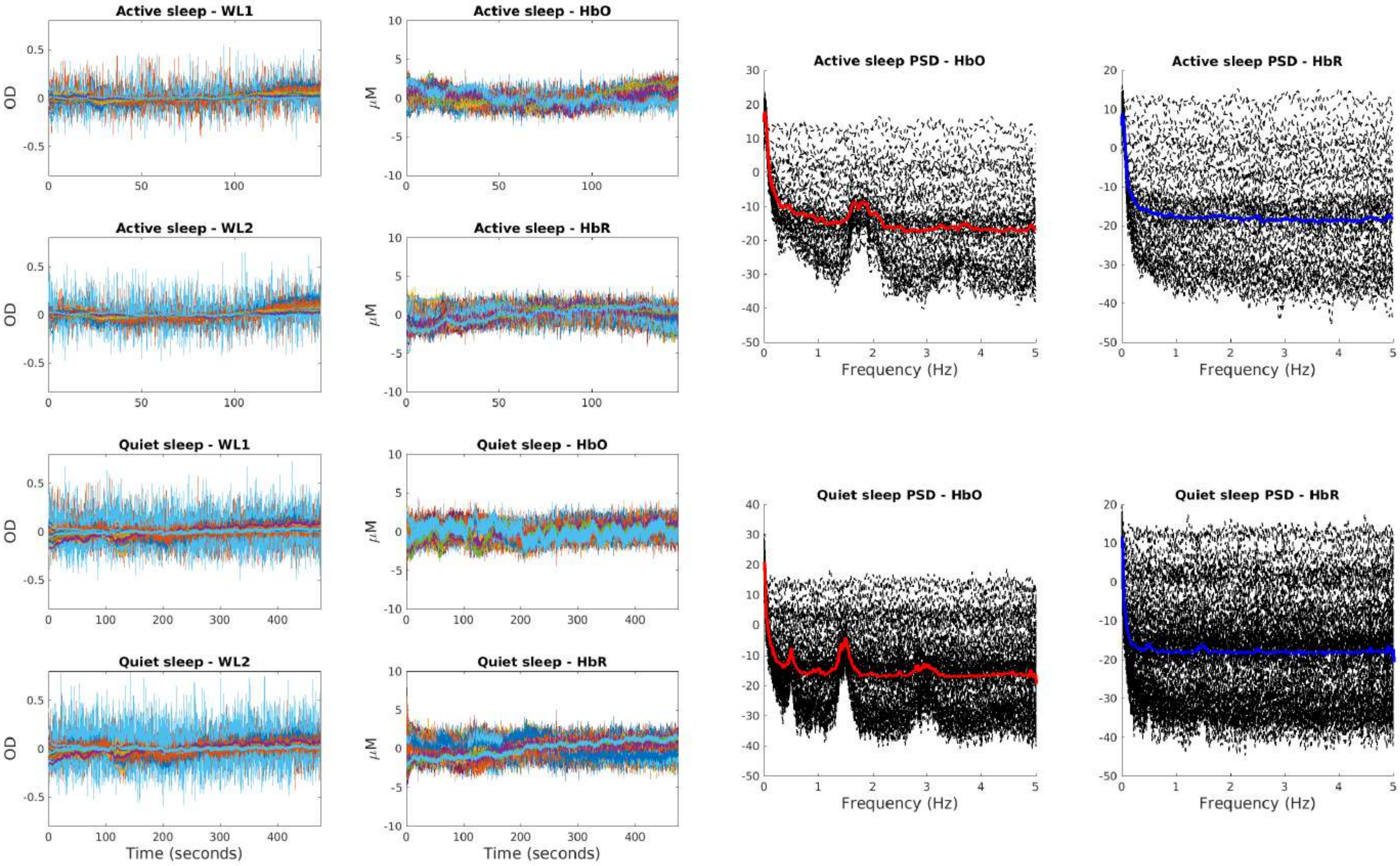
Active sleep - WL1
Active sleep - HbO
Active sleep PSD - HbO
Active sleep PSD - HbR
Active sleep - WL2
Active sleep - HbR
Quiet sleep - WL1
Quiet sleep - HbO
Quiet sleep PSD - HbO
Quiet sleep PSD - HbR
Quiet sleep - WL2
Quiet sleep - HbR
OD
μM
Time (seconds)
Frequency (Hz)

HT_012

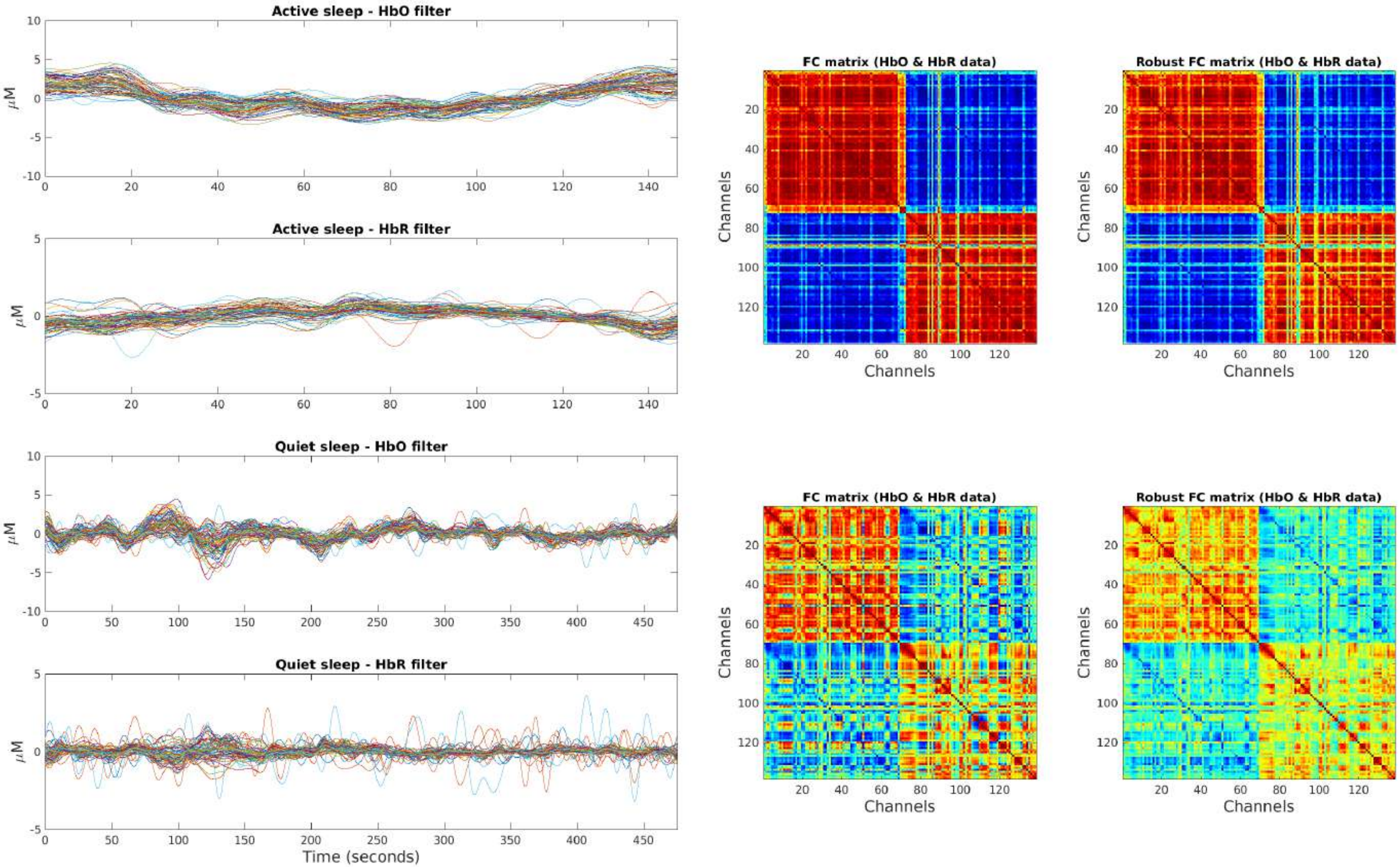
Active sleep - HbO filter
Active sleep - HbR filter
Quiet sleep - HbO filter
Quiet sleep - HbR filter
μM
Time (seconds)
FC matrix (HbO & HbR data)
Robust FC matrix (HbO & HbR data)
Channels

HT_012

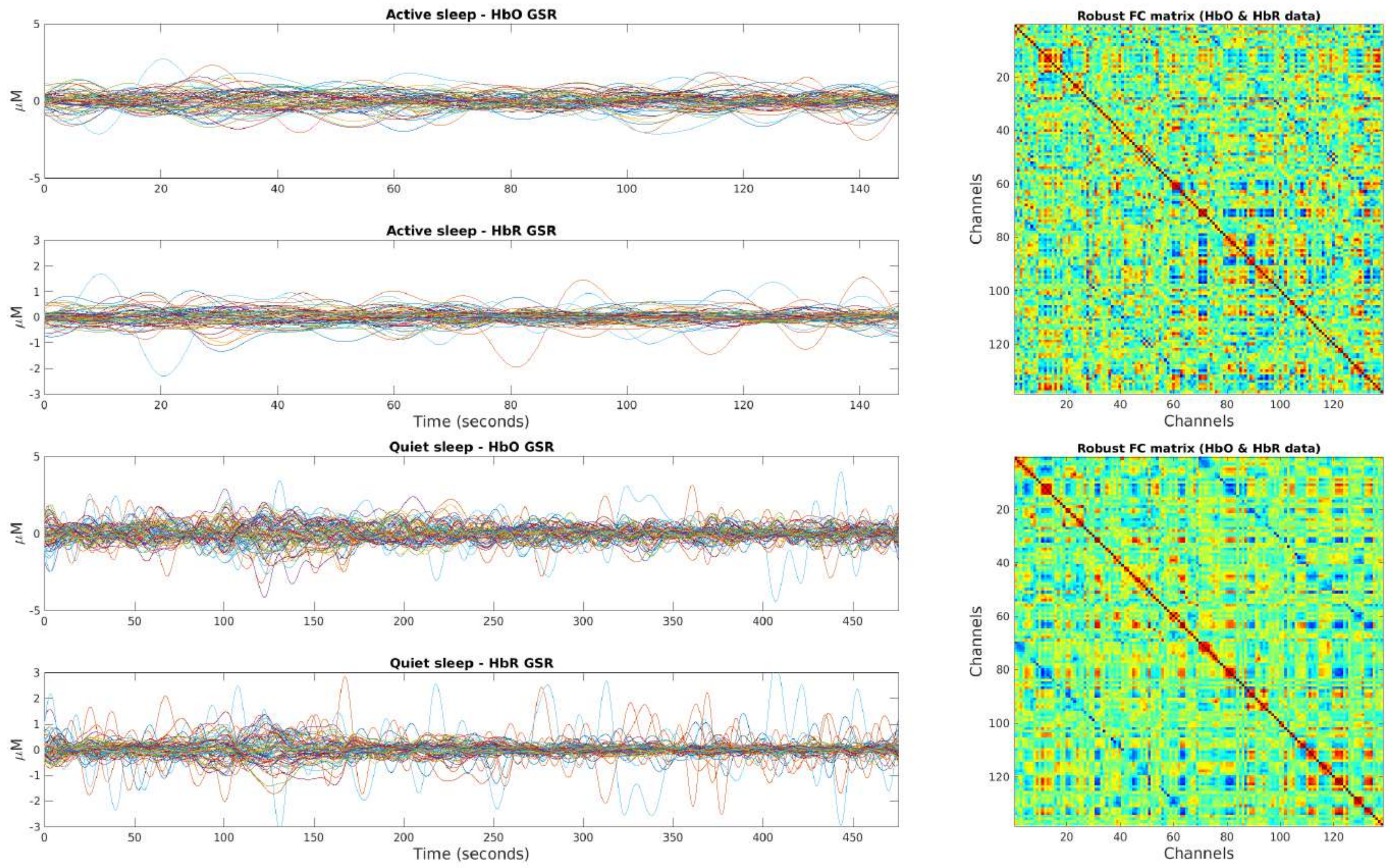
Active sleep - HbO GSR
Active sleep - HbR GSR
Quiet sleep - HbO GSR
Quiet sleep - HbR GSR
μM
Time (seconds)
Robust FC matrix (HbO & HbR data)
Channels

HT_013

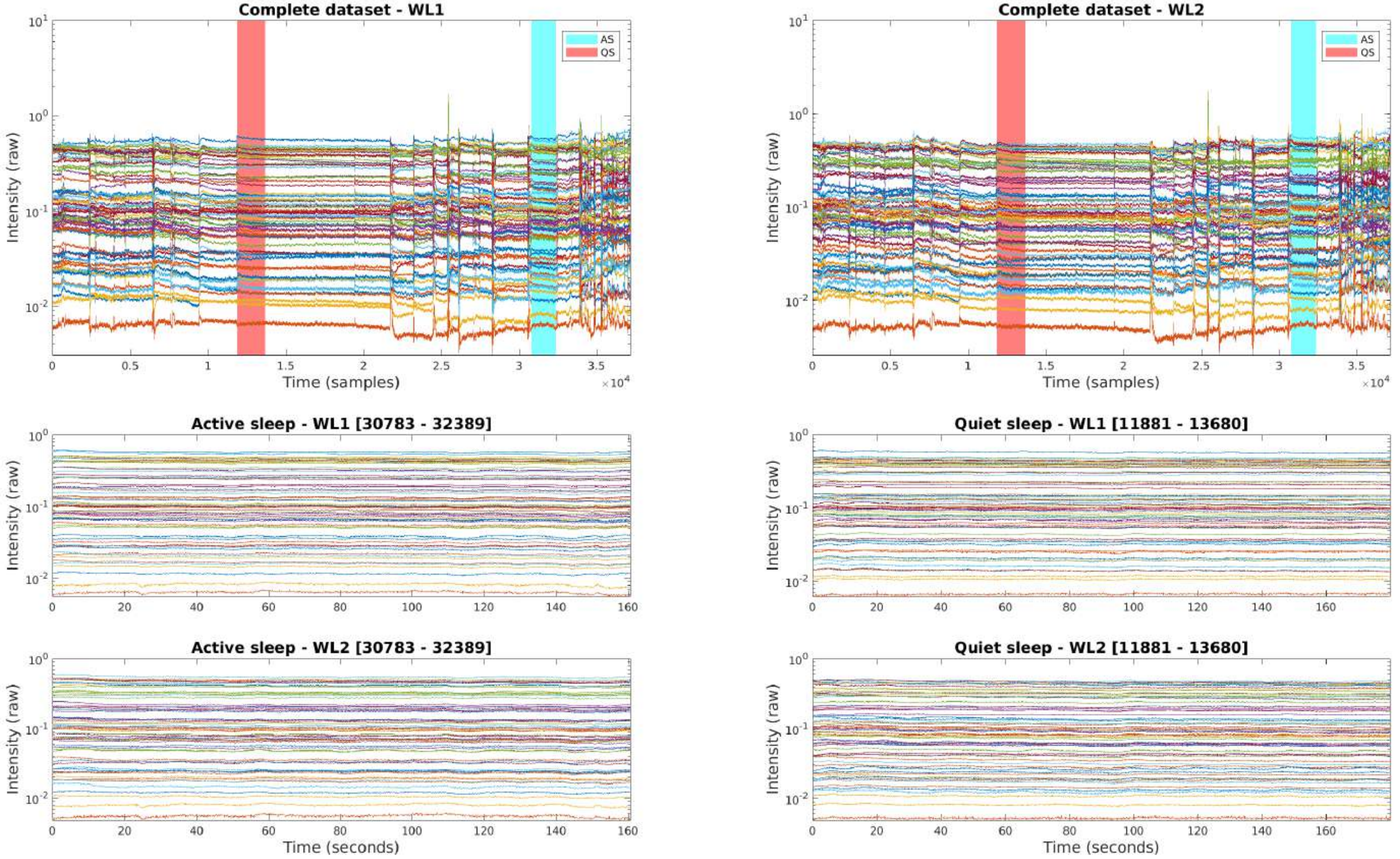
Complete dataset - WL1
Complete dataset - WL2
AS
QS
Intensity (raw)
Time (samples)
×10^4
Active sleep - WL1 [30783 - 32389]
Quiet sleep - WL1 [11881 - 13680]
Active sleep - WL2 [30783 - 32389]
Quiet sleep - WL2 [11881 - 13680]
Time (seconds)

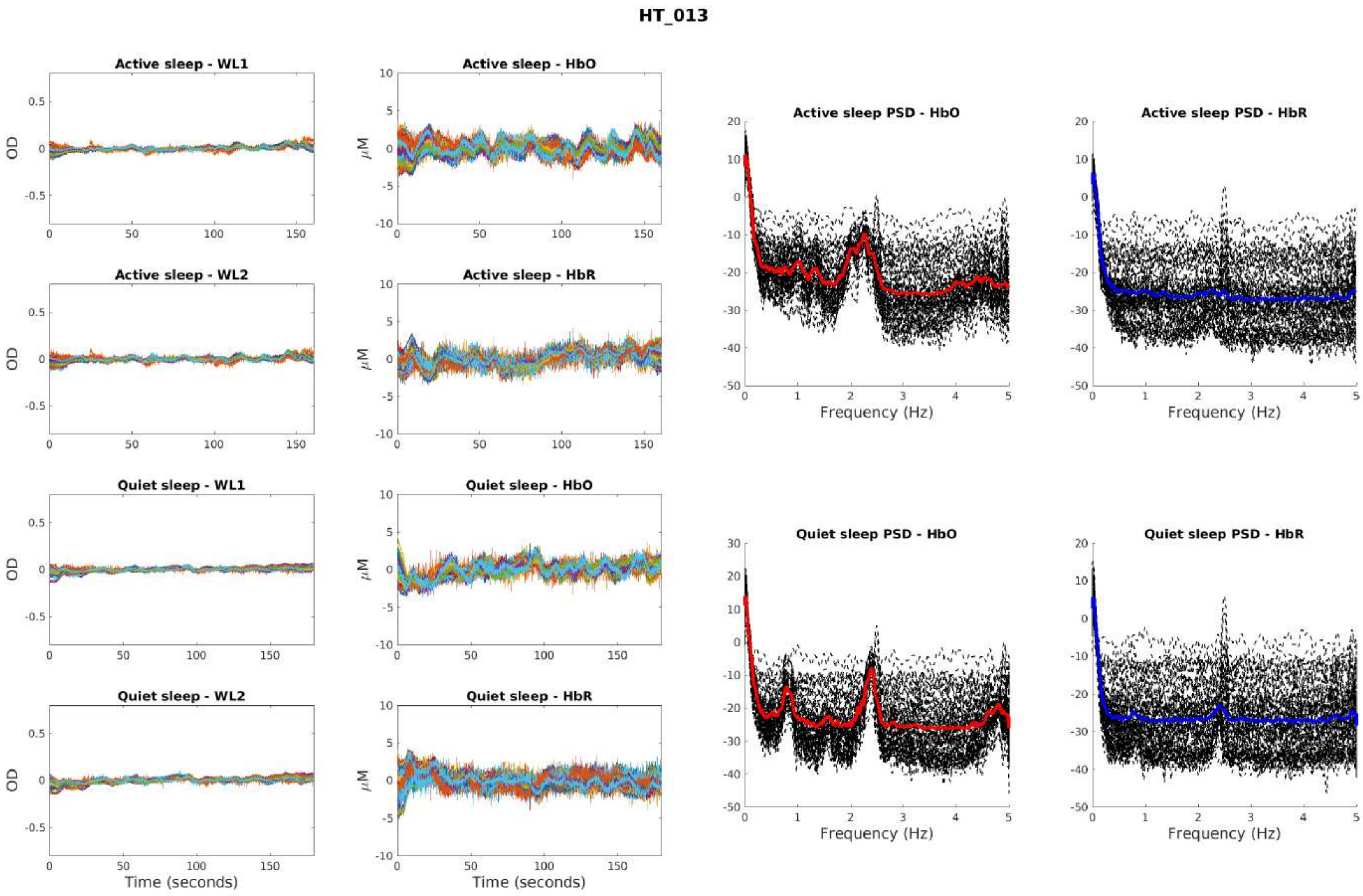

HT_013
Active sleep - WL1
Active sleep - HbO
Active sleep PSD - HbO
Active sleep PSD - HbR
Active sleep - WL2
Active sleep - HbR
Quiet sleep - WL1
Quiet sleep - HbO
Quiet sleep PSD - HbO
Quiet sleep PSD - HbR
Quiet sleep - WL2
Quiet sleep - HbR
OD
μM
Time (seconds)
Frequency (Hz)

HT_013

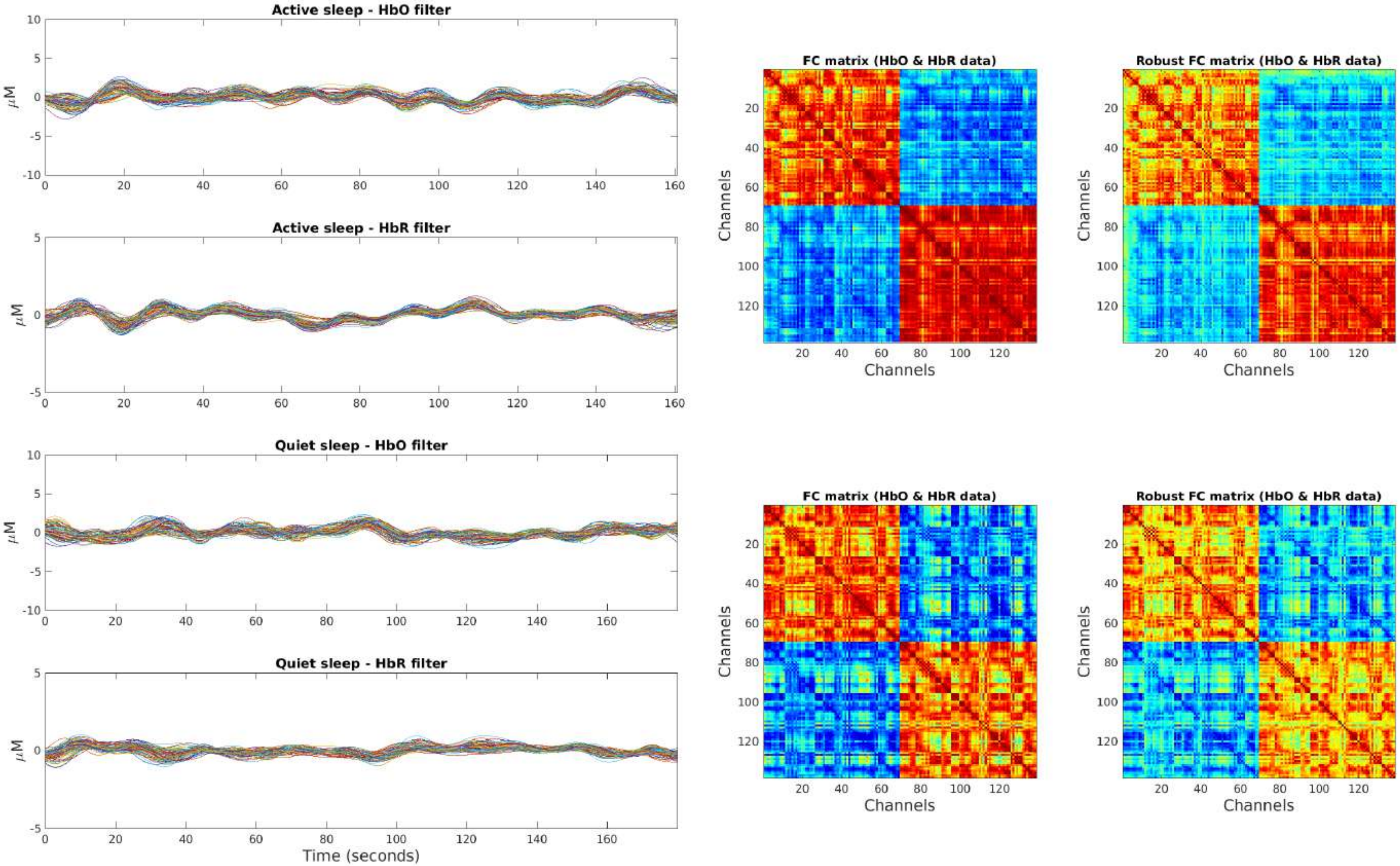

Active sleep - HbO filter
Active sleep - HbR filter
Quiet sleep - HbO filter
Quiet sleep - HbR filter
μM
Time (seconds)
FC matrix (HbO & HbR data)
Robust FC matrix (HbO & HbR data)
Channels

HT_013

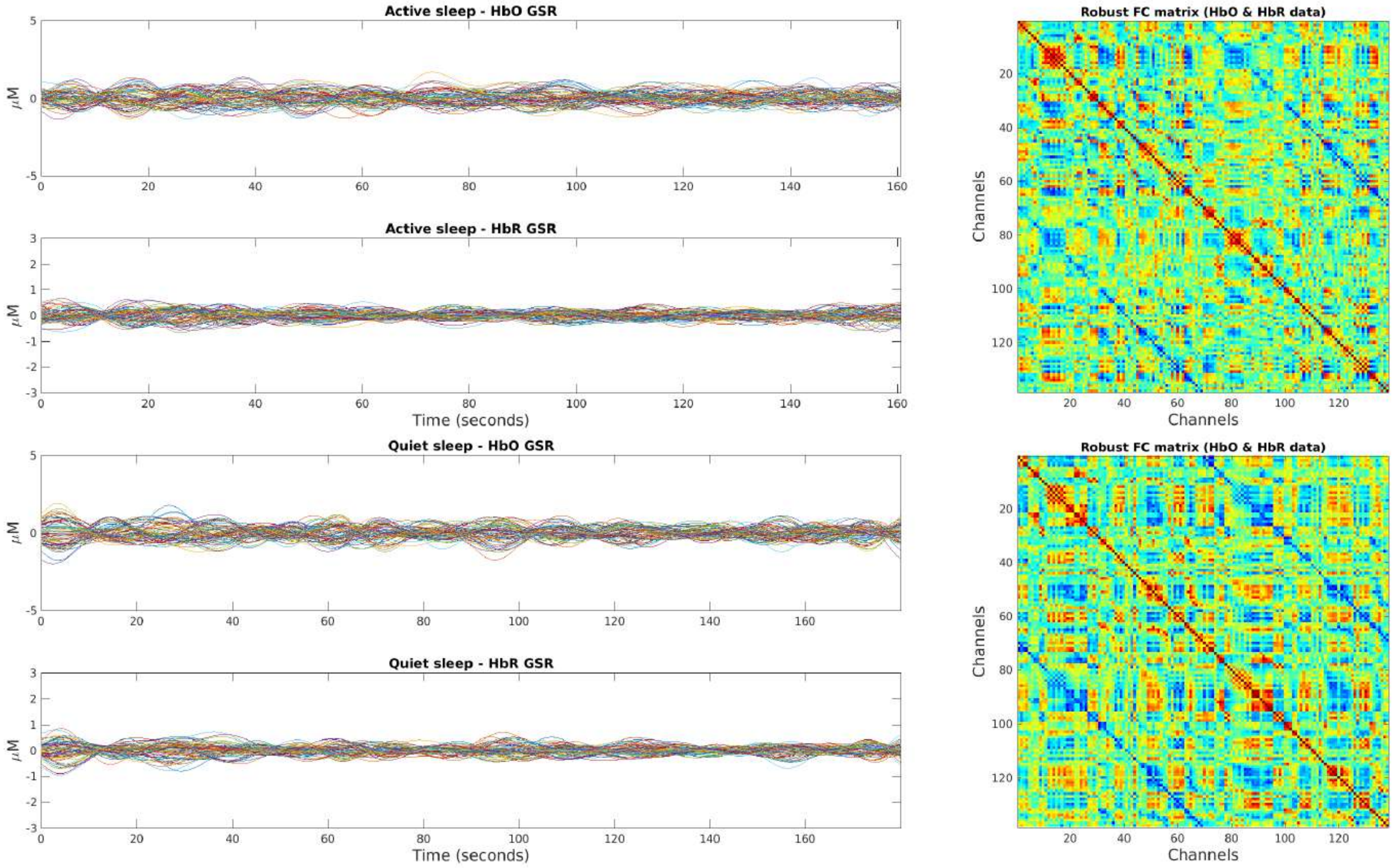

Active sleep - HbO GSR
Active sleep - HbR GSR
Quiet sleep - HbO GSR
Quiet sleep - HbR GSR
μM
Time (seconds)
Robust FC matrix (HbO & HbR data)
Channels

HT_015

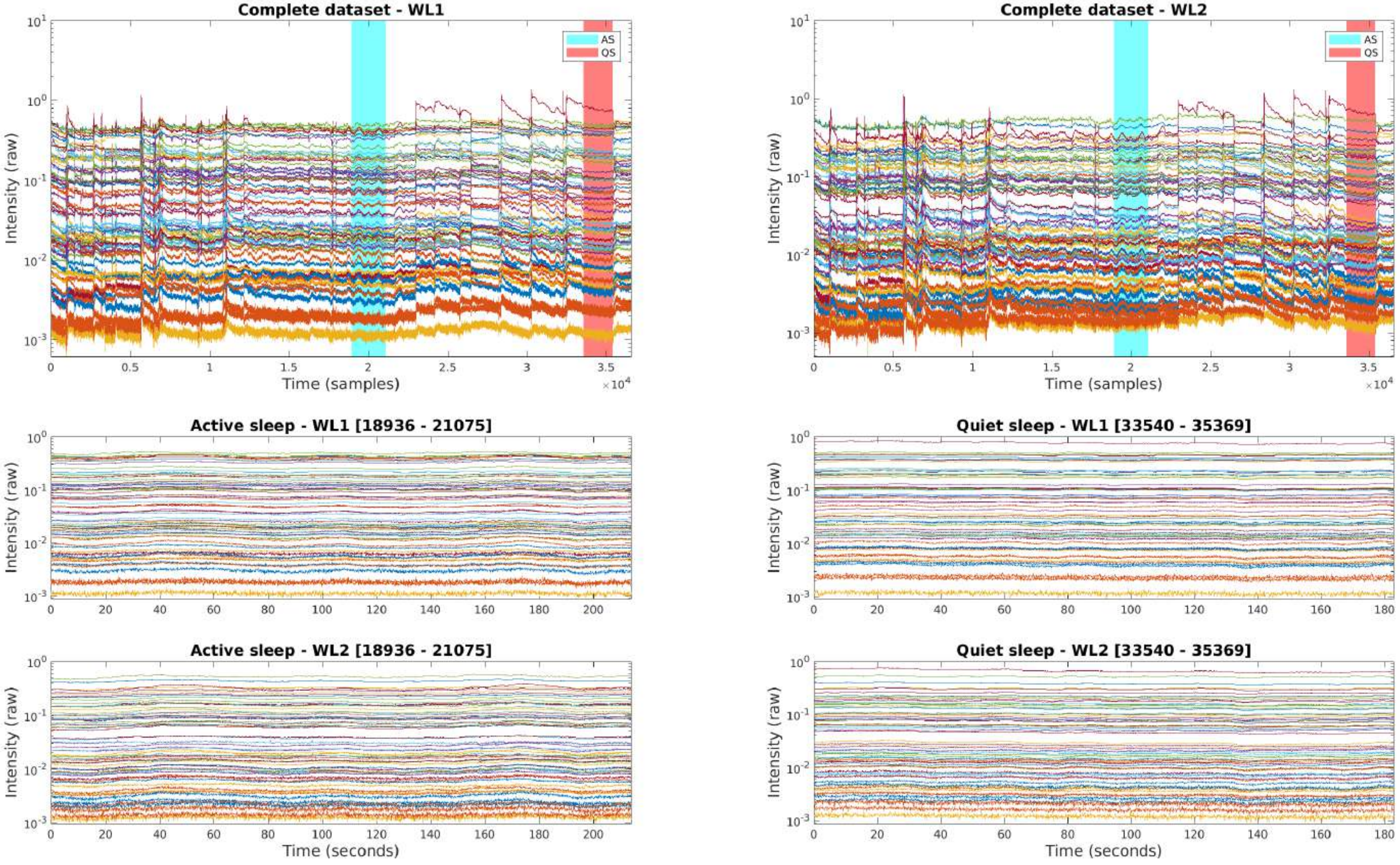

Complete dataset - WL1
Complete dataset - WL2
AS
QS
Intensity (raw)
Time (samples)
$\times 10^4$
Active sleep - WL1 [18936 - 21075]
Quiet sleep - WL1 [33540 - 35369]
Active sleep - WL2 [18936 - 21075]
Quiet sleep - WL2 [33540 - 35369]
Time (seconds)

HT_015

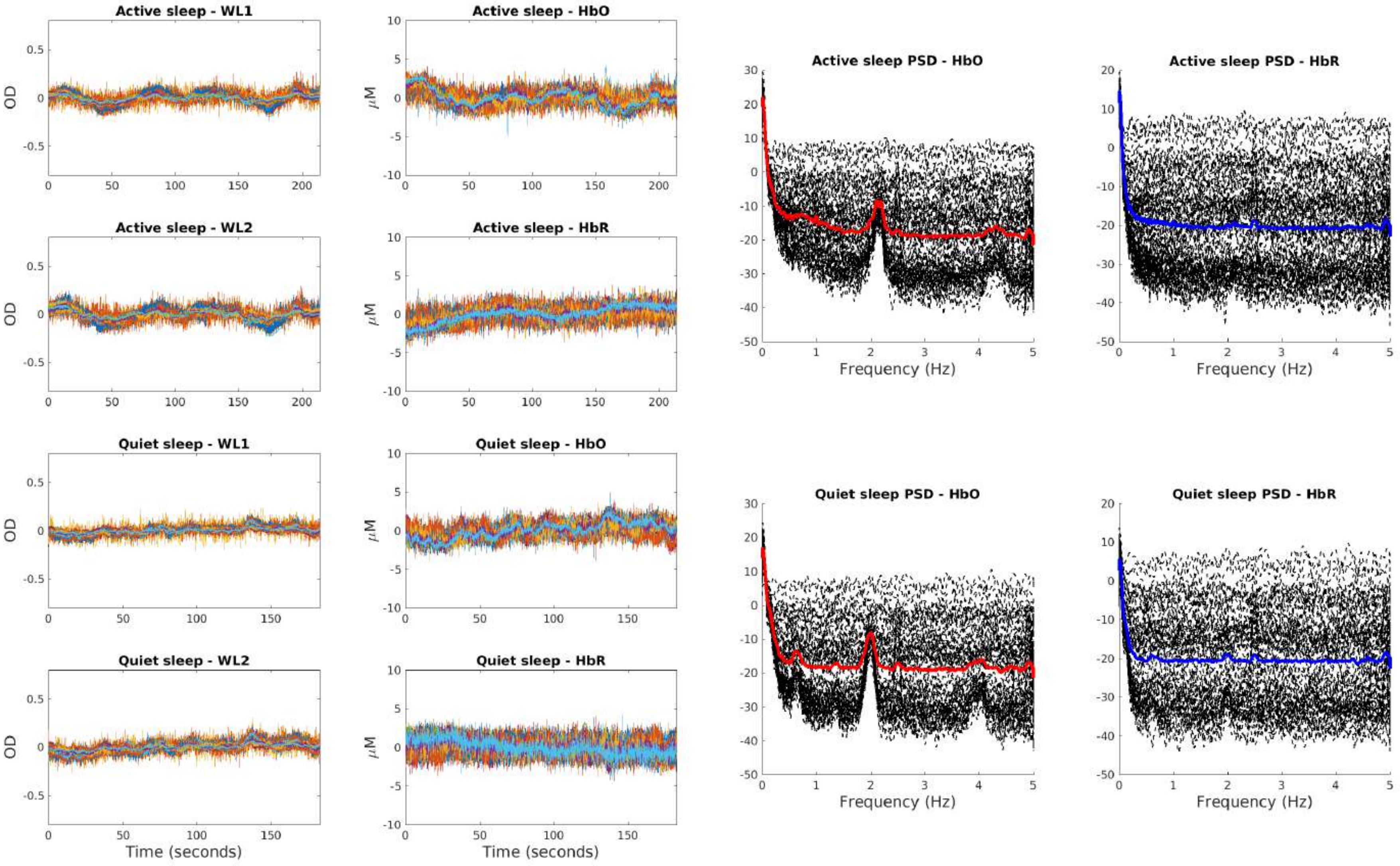

Active sleep - WL1
Active sleep - WL2
Quiet sleep - WL1
Quiet sleep - WL2
OD
Time (seconds)
Active sleep - HbO
Active sleep - HbR
Quiet sleep - HbO
Quiet sleep - HbR
μM
Active sleep PSD - HbO
Active sleep PSD - HbR
Quiet sleep PSD - HbO
Quiet sleep PSD - HbR
Frequency (Hz)

HT_015

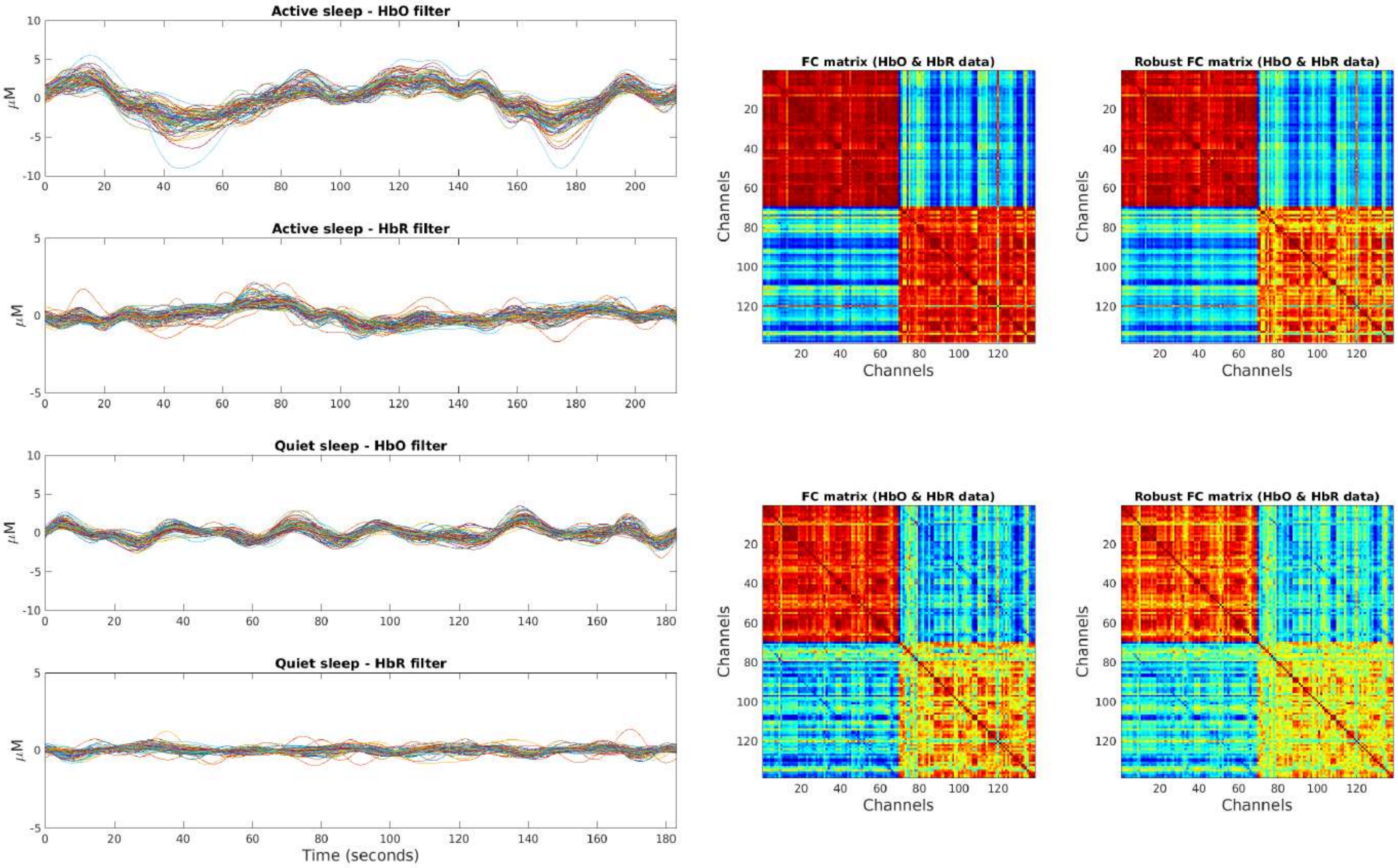

Active sleep - HbO filter
Active sleep - HbR filter
Quiet sleep - HbO filter
Quiet sleep - HbR filter
μM
Time (seconds)
FC matrix (HbO & HbR data)
Robust FC matrix (HbO & HbR data)
Channels

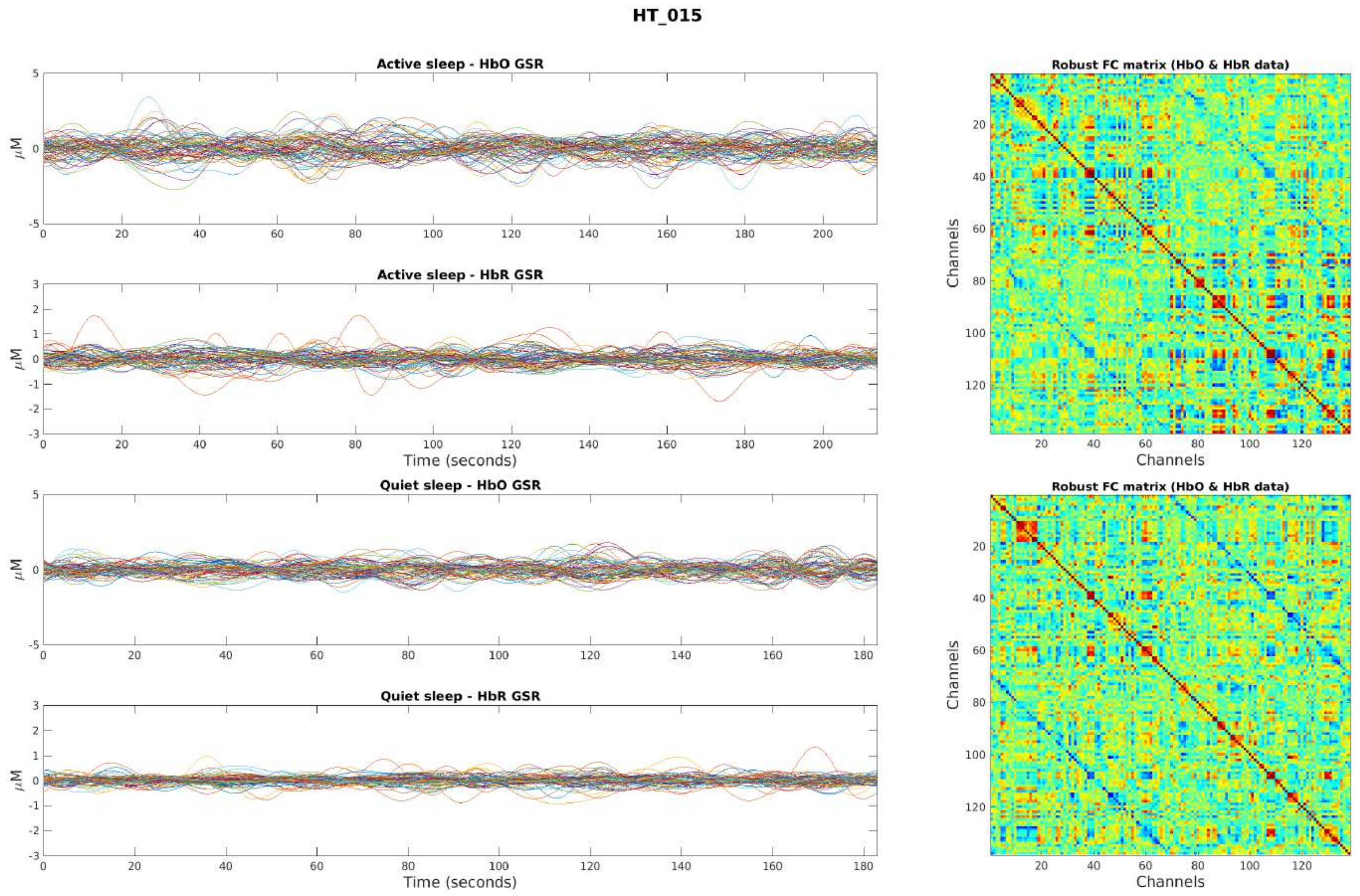
HT_015
Active sleep - HbO GSR
Active sleep - HbR GSR
Quiet sleep - HbO GSR
Quiet sleep - HbR GSR
μM
Time (seconds)
Robust FC matrix (HbO & HbR data)
Channels

HT_016

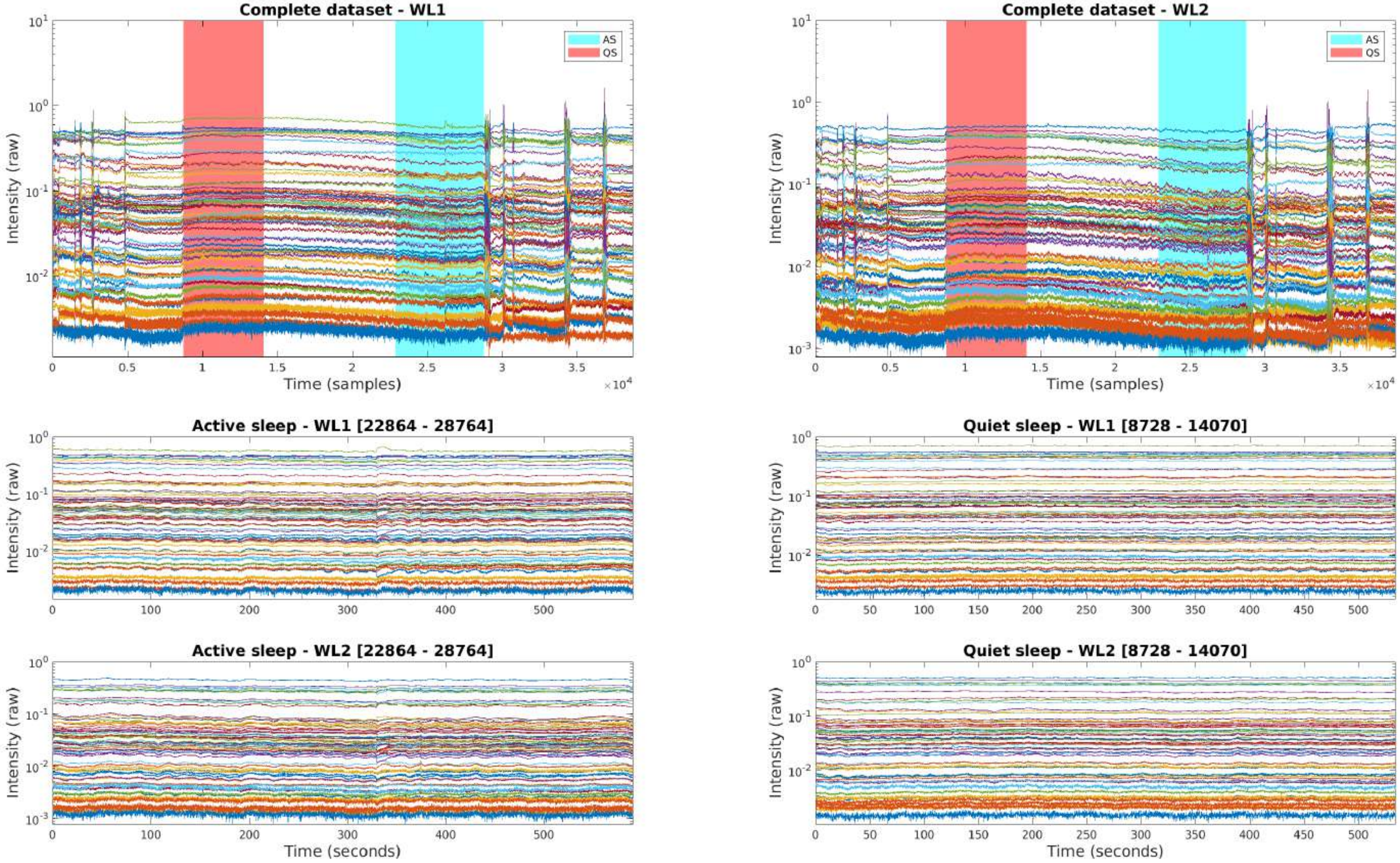
Complete dataset - WL1
Complete dataset - WL2
AS
QS
Intensity (raw)
Time (samples)
×10^4
Active sleep - WL1 [22864 - 28764]
Quiet sleep - WL1 [8728 - 14070]
Active sleep - WL2 [22864 - 28764]
Quiet sleep - WL2 [8728 - 14070]
Time (seconds)

HT_016

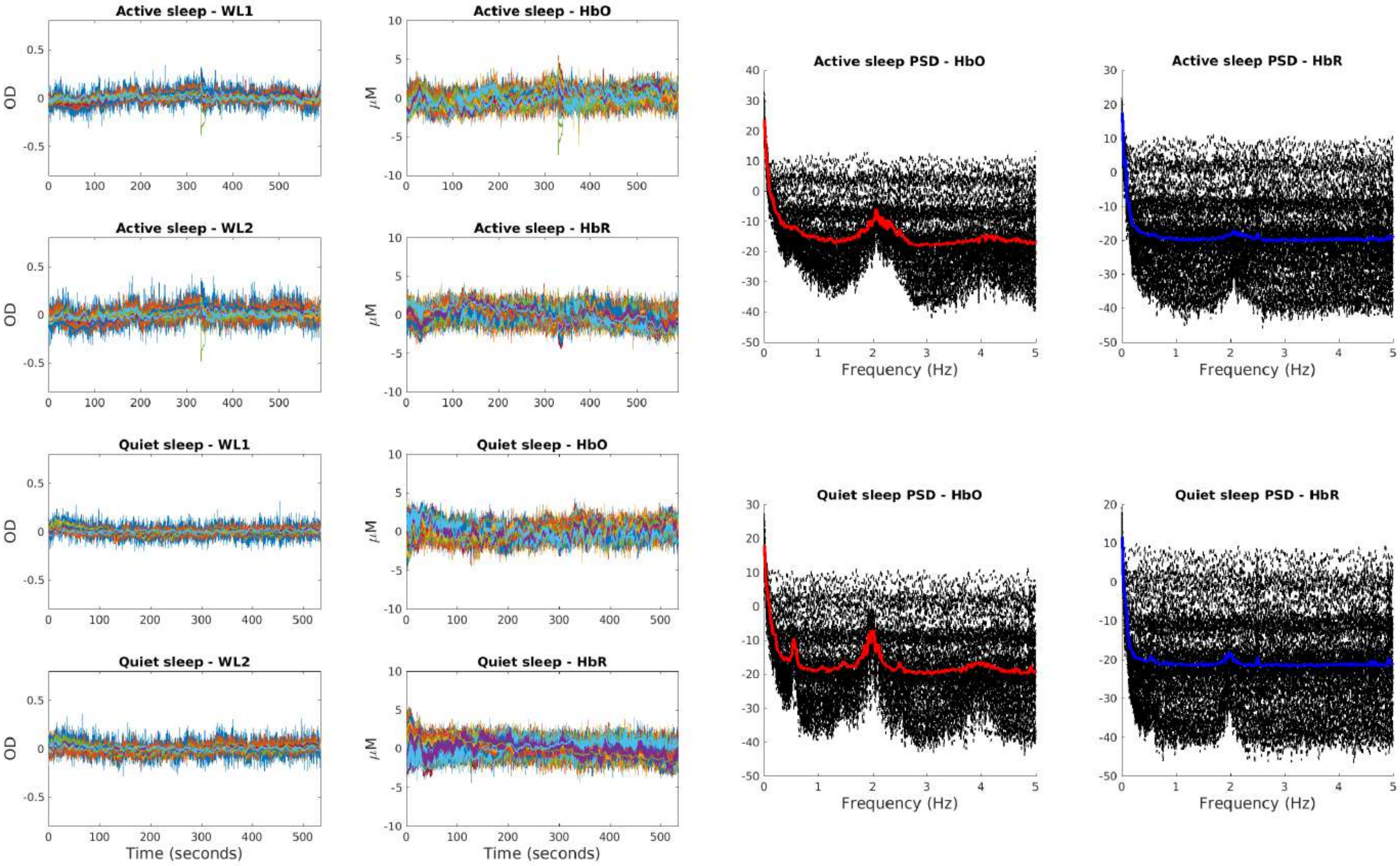

Active sleep - WL1
Active sleep - HbO
Active sleep PSD - HbO
Active sleep PSD - HbR
Active sleep - WL2
Active sleep - HbR
Quiet sleep - WL1
Quiet sleep - HbO
Quiet sleep PSD - HbO
Quiet sleep PSD - HbR
Quiet sleep - WL2
Quiet sleep - HbR
OD
μM
Time (seconds)
Frequency (Hz)

HT_016

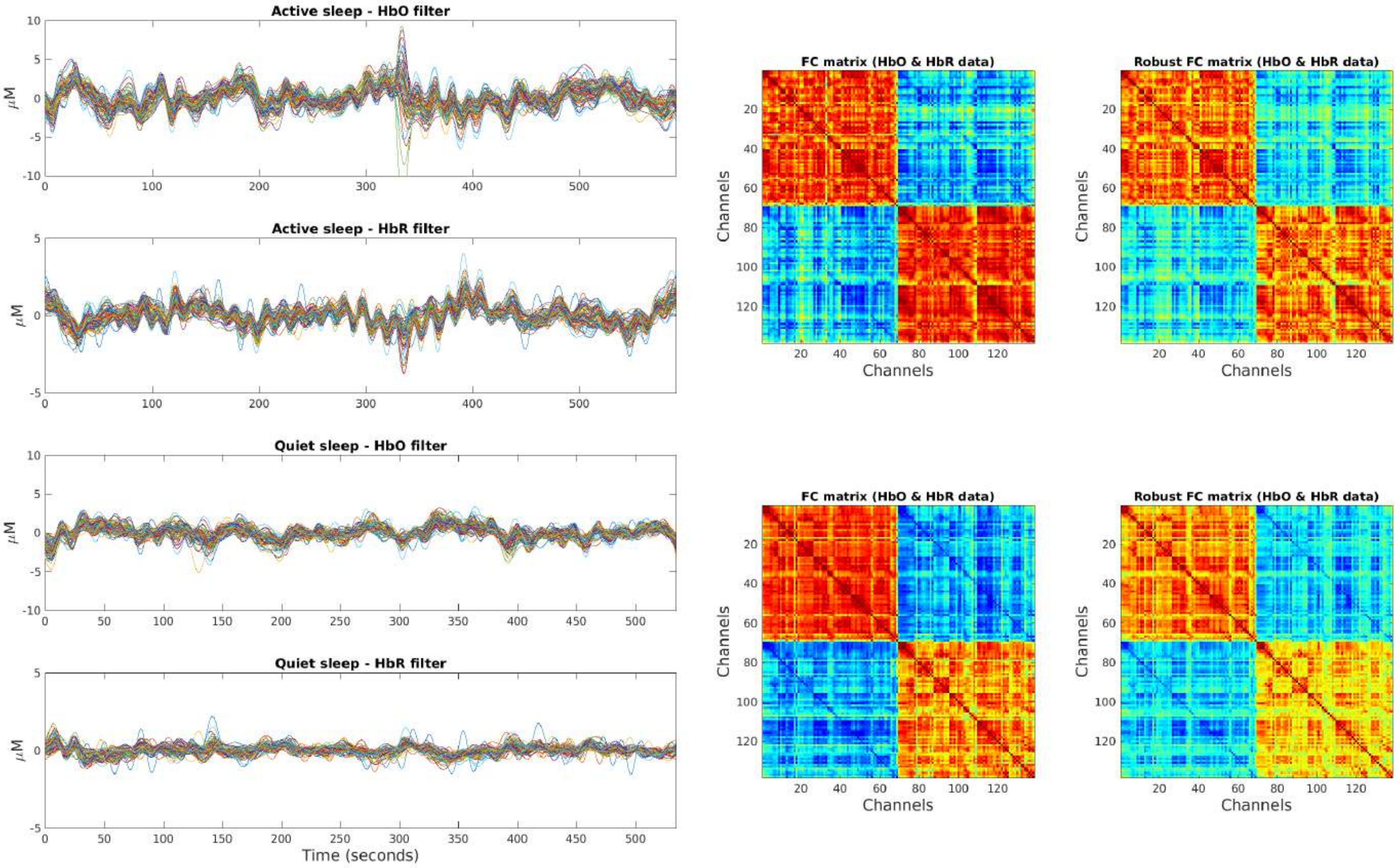
Active sleep - HbO filter
Active sleep - HbR filter
Quiet sleep - HbO filter
Quiet sleep - HbR filter
μM
Time (seconds)
FC matrix (HbO & HbR data)
Robust FC matrix (HbO & HbR data)
Channels

HT_016

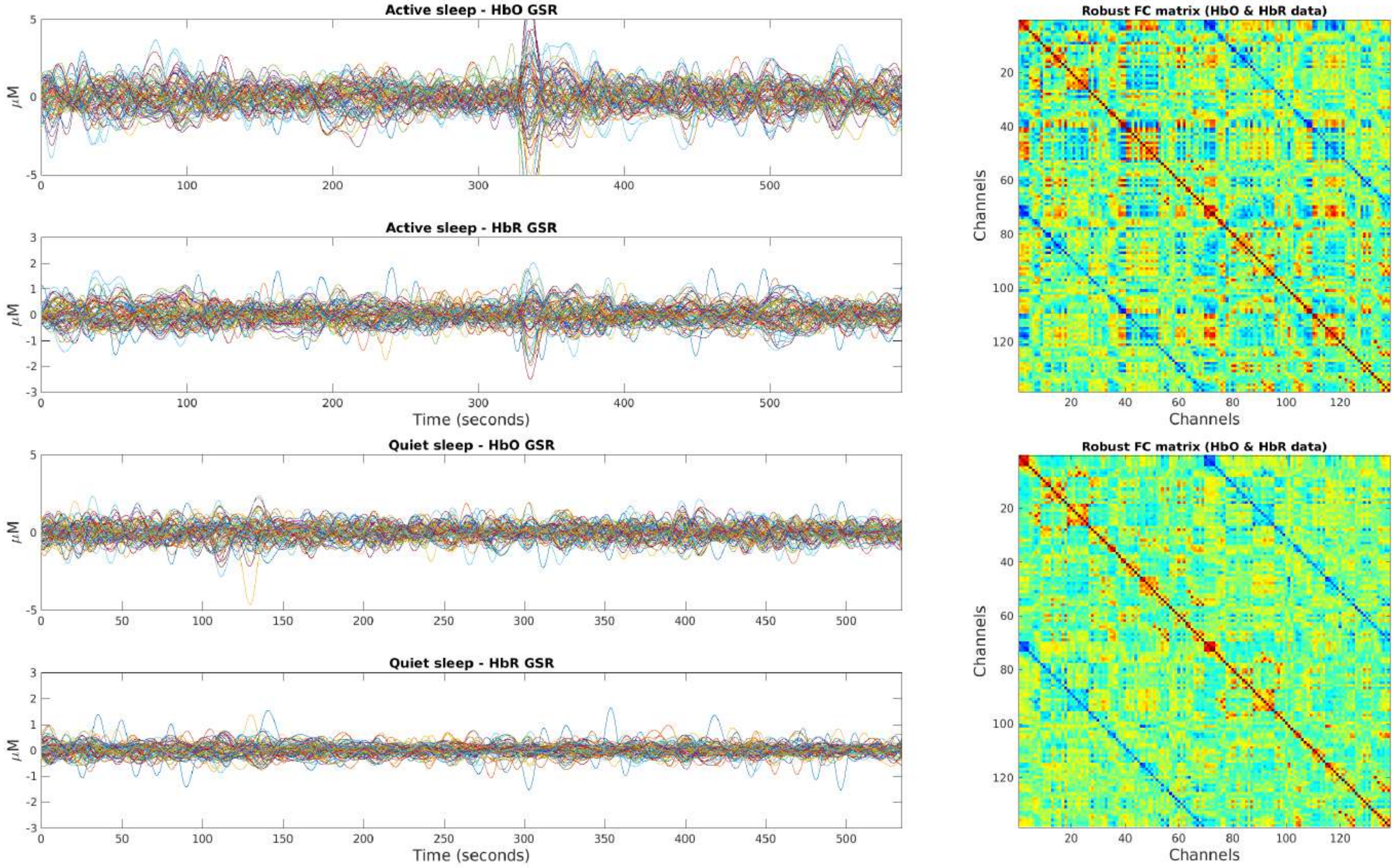
Active sleep - HbO GSR
Active sleep - HbR GSR
Quiet sleep - HbO GSR
Quiet sleep - HbR GSR
μM
Time (seconds)
Robust FC matrix (HbO & HbR data)
Channels

HT_018

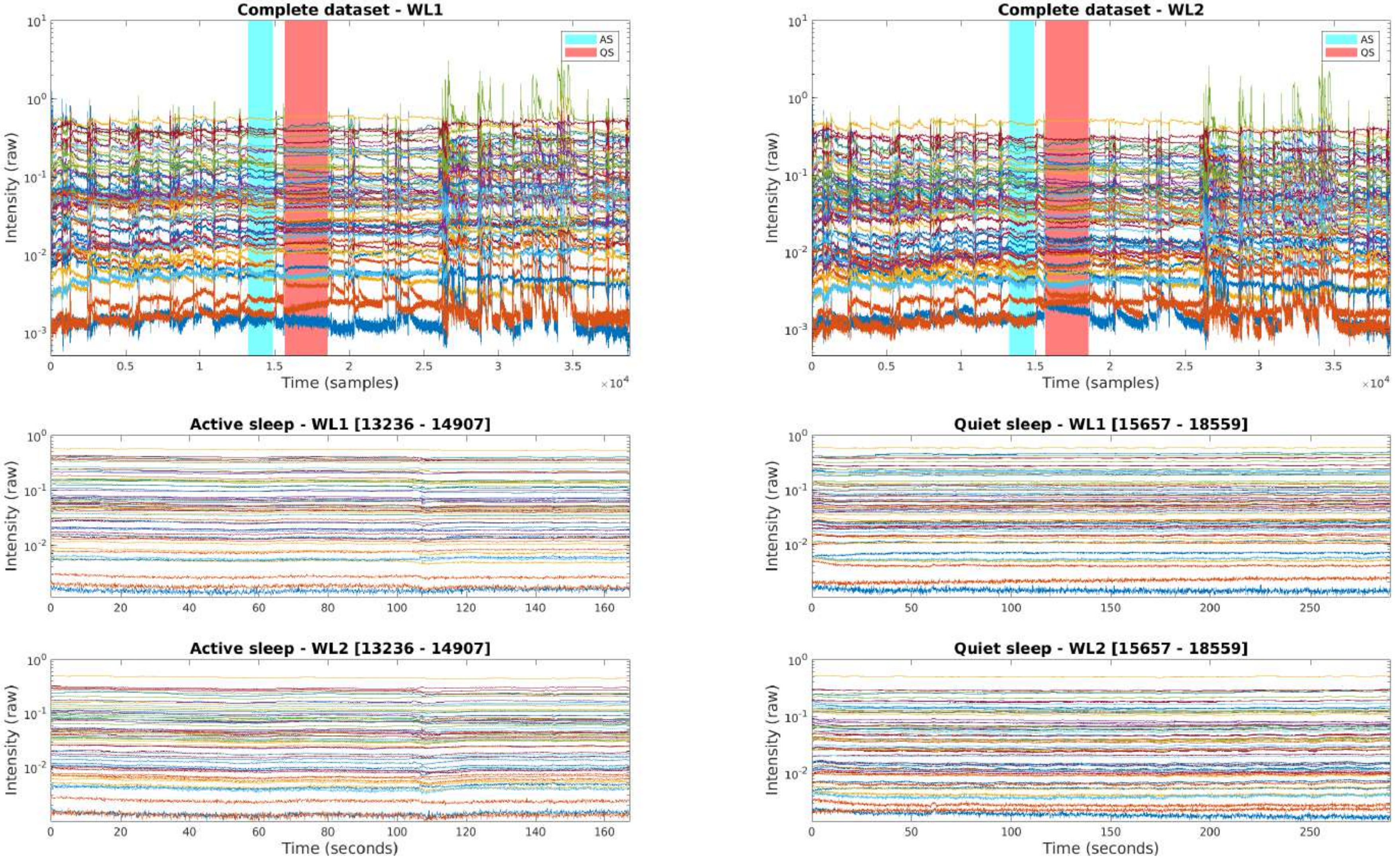
Complete dataset - WL1
Complete dataset - WL2
AS
QS
Intensity (raw)
Time (samples)
×10^4
Active sleep - WL1 [13236 - 14907]
Quiet sleep - WL1 [15657 - 18559]
Active sleep - WL2 [13236 - 14907]
Quiet sleep - WL2 [15657 - 18559]
Time (seconds)

HT_018

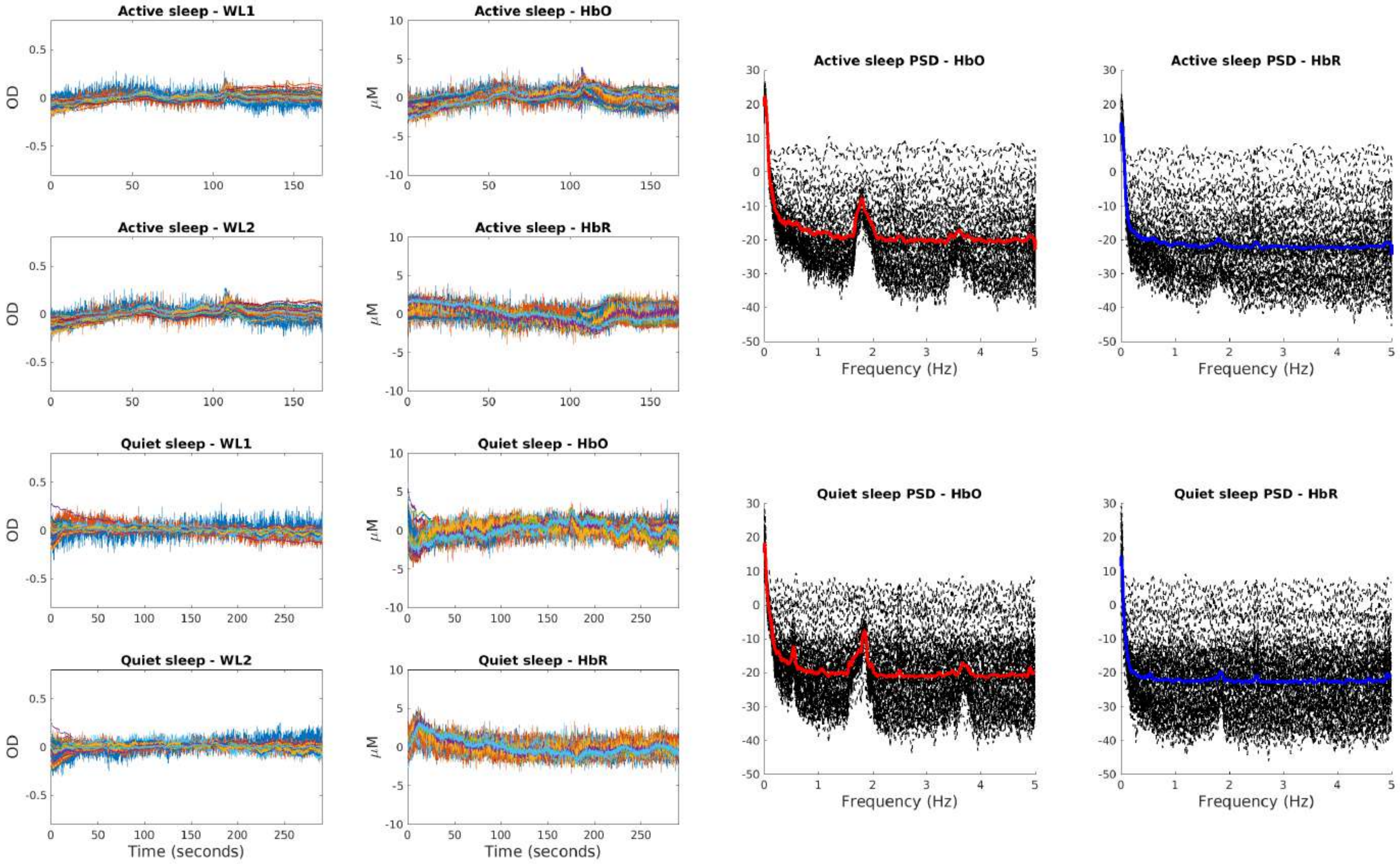

Active sleep - WL1
Active sleep - HbO
Active sleep PSD - HbO
Active sleep PSD - HbR
Active sleep - WL2
Active sleep - HbR
Quiet sleep - WL1
Quiet sleep - HbO
Quiet sleep PSD - HbO
Quiet sleep PSD - HbR
Quiet sleep - WL2
Quiet sleep - HbR
OD
μM
Time (seconds)
Frequency (Hz)

HT_018

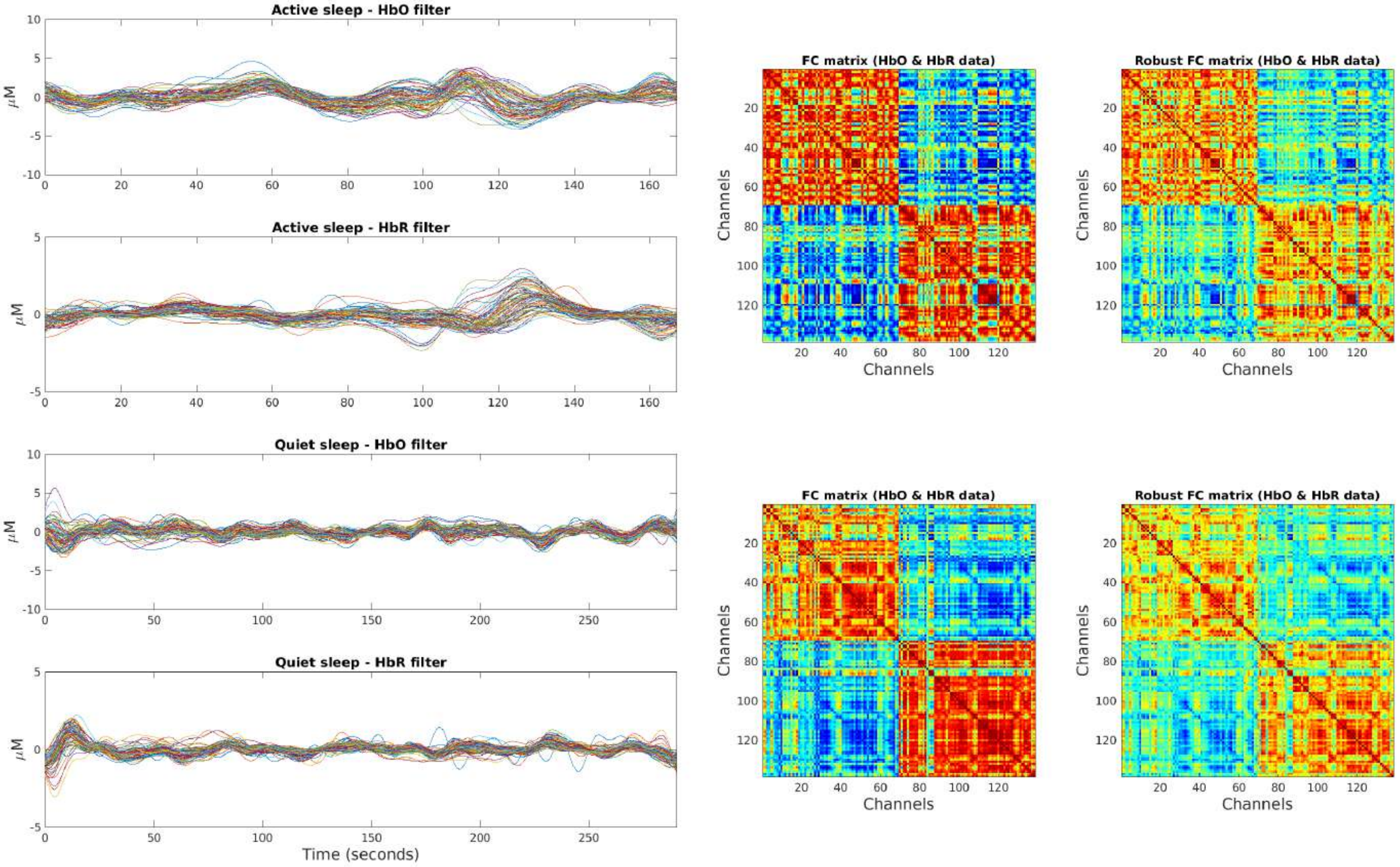

Active sleep - HbO filter
Active sleep - HbR filter
Quiet sleep - HbO filter
Quiet sleep - HbR filter
μM
Time (seconds)
FC matrix (HbO & HbR data)
Robust FC matrix (HbO & HbR data)
FC matrix (HbO & HbR data)
Robust FC matrix (HbO & HbR data)
Channels

HT_018

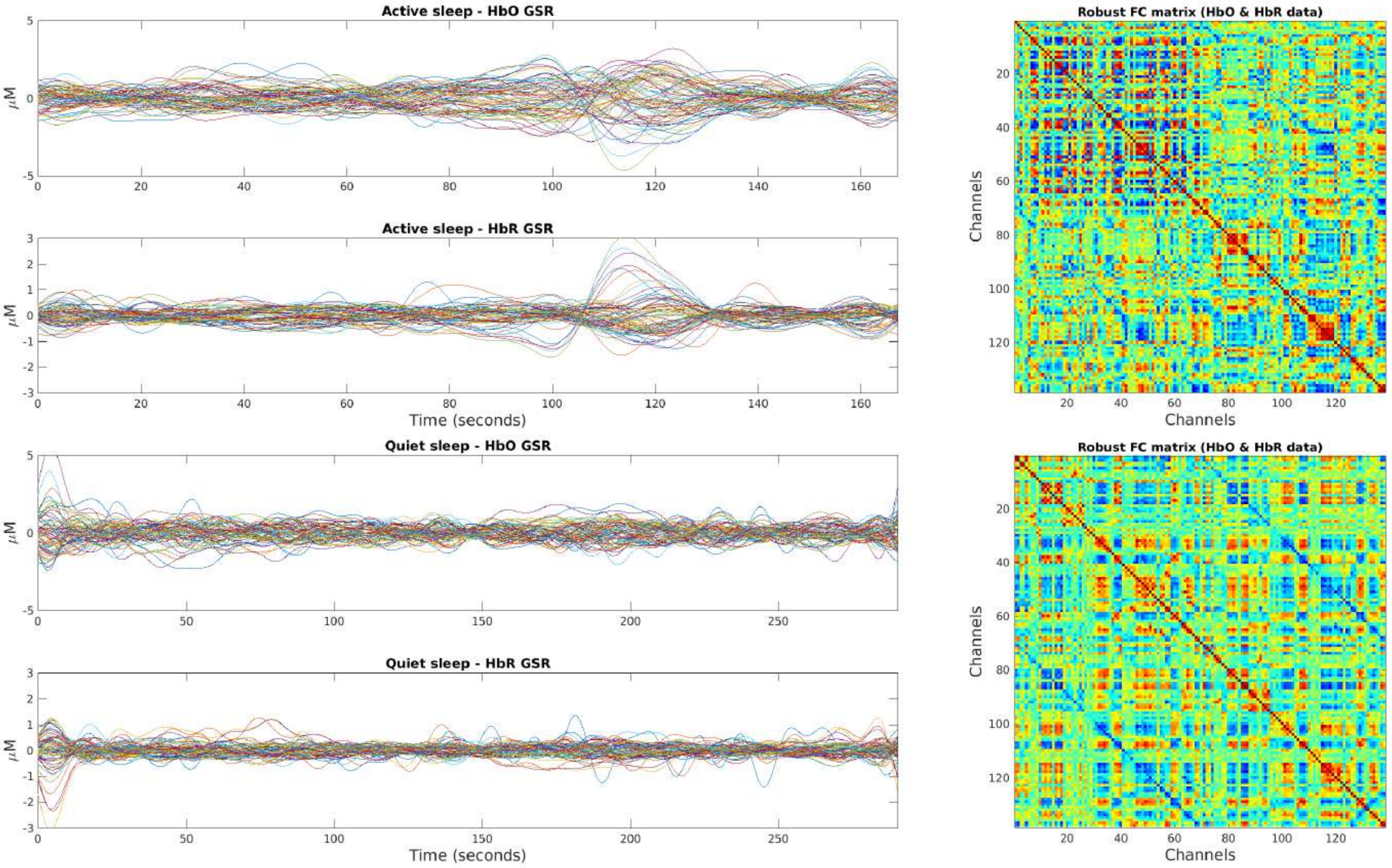

Active sleep - HbO GSR
Active sleep - HbR GSR
Quiet sleep - HbO GSR
Quiet sleep - HbR GSR
μM
Time (seconds)
Robust FC matrix (HbO & HbR data)
Robust FC matrix (HbO & HbR data)
Channels
Channels

HT_019

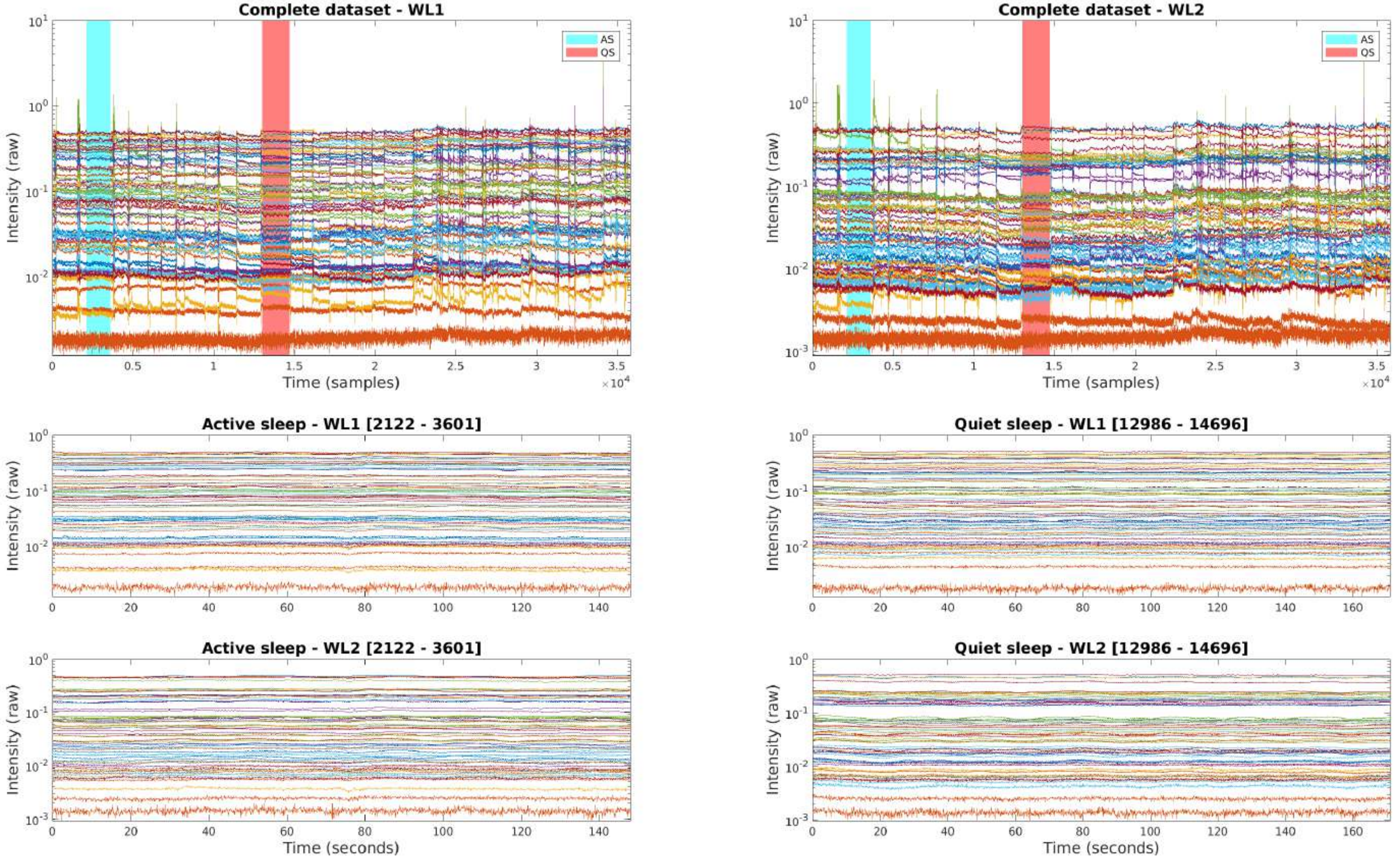

Complete dataset - WL1
Complete dataset - WL2
AS
QS
Intensity (raw)
Time (samples)
Active sleep - WL1 [2122 - 3601]
Quiet sleep - WL1 [12986 - 14696]
Active sleep - WL2 [2122 - 3601]
Quiet sleep - WL2 [12986 - 14696]
Time (seconds)

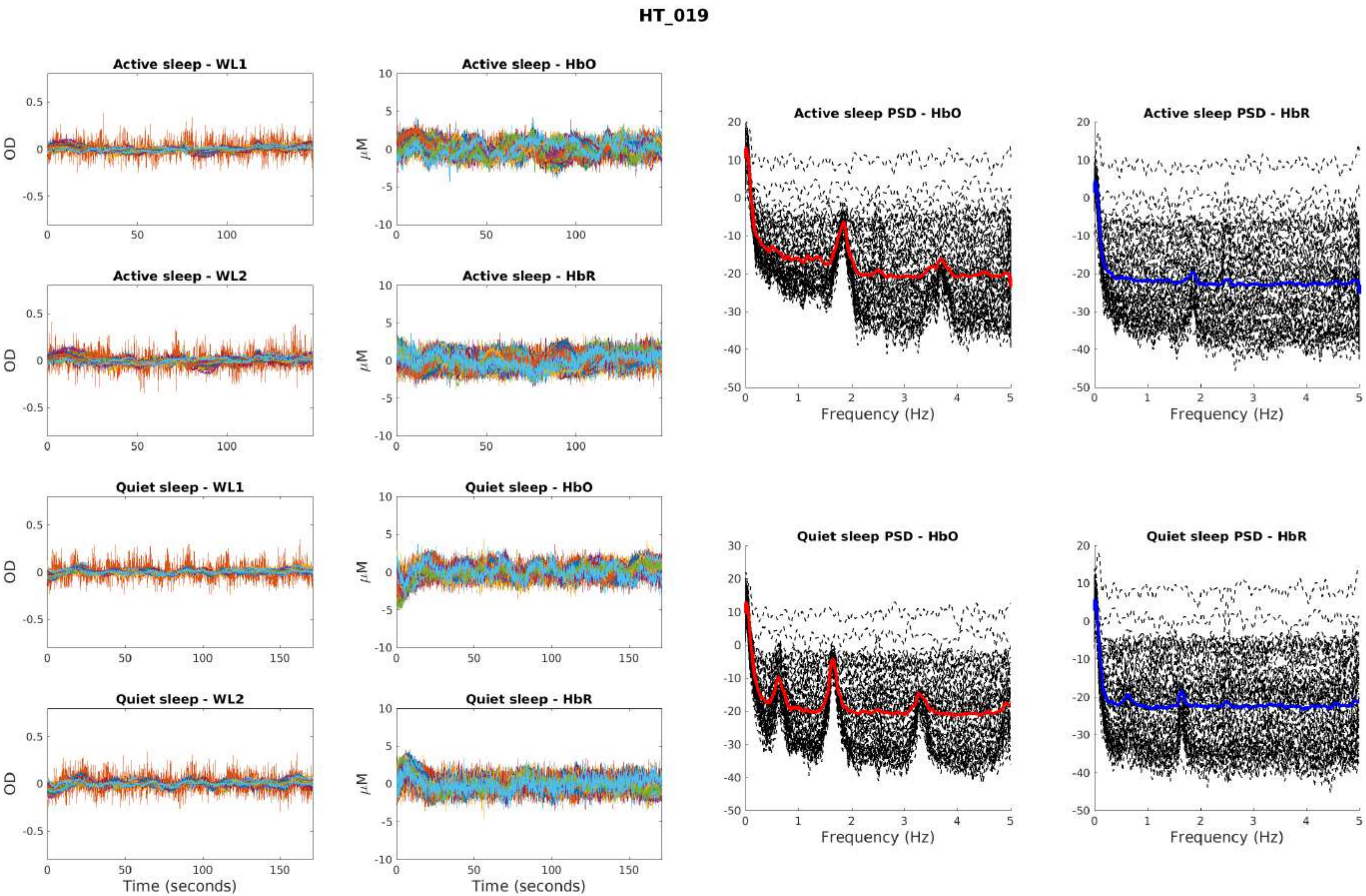
HT_019
Active sleep - WL1
Active sleep - HbO
Active sleep PSD - HbO
Active sleep PSD - HbR
Active sleep - WL2
Active sleep - HbR
Quiet sleep - WL1
Quiet sleep - HbO
Quiet sleep PSD - HbO
Quiet sleep PSD - HbR
Quiet sleep - WL2
Quiet sleep - HbR
OD
μM
Time (seconds)
Frequency (Hz)

HT_019

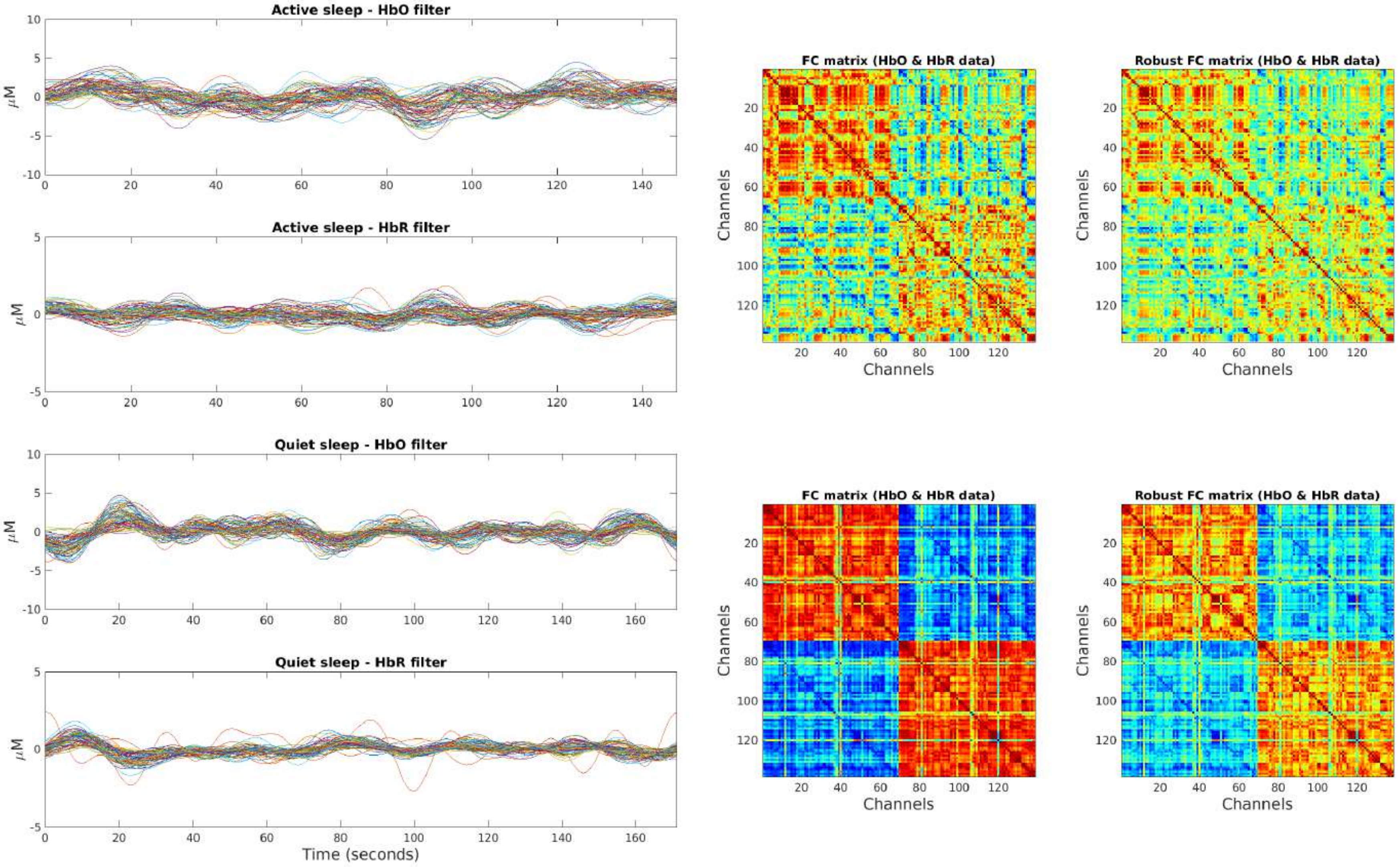
Active sleep - HbO filter
Active sleep - HbR filter
Quiet sleep - HbO filter
Quiet sleep - HbR filter
μM
Time (seconds)
FC matrix (HbO & HbR data)
Robust FC matrix (HbO & HbR data)
Channels

HT_019

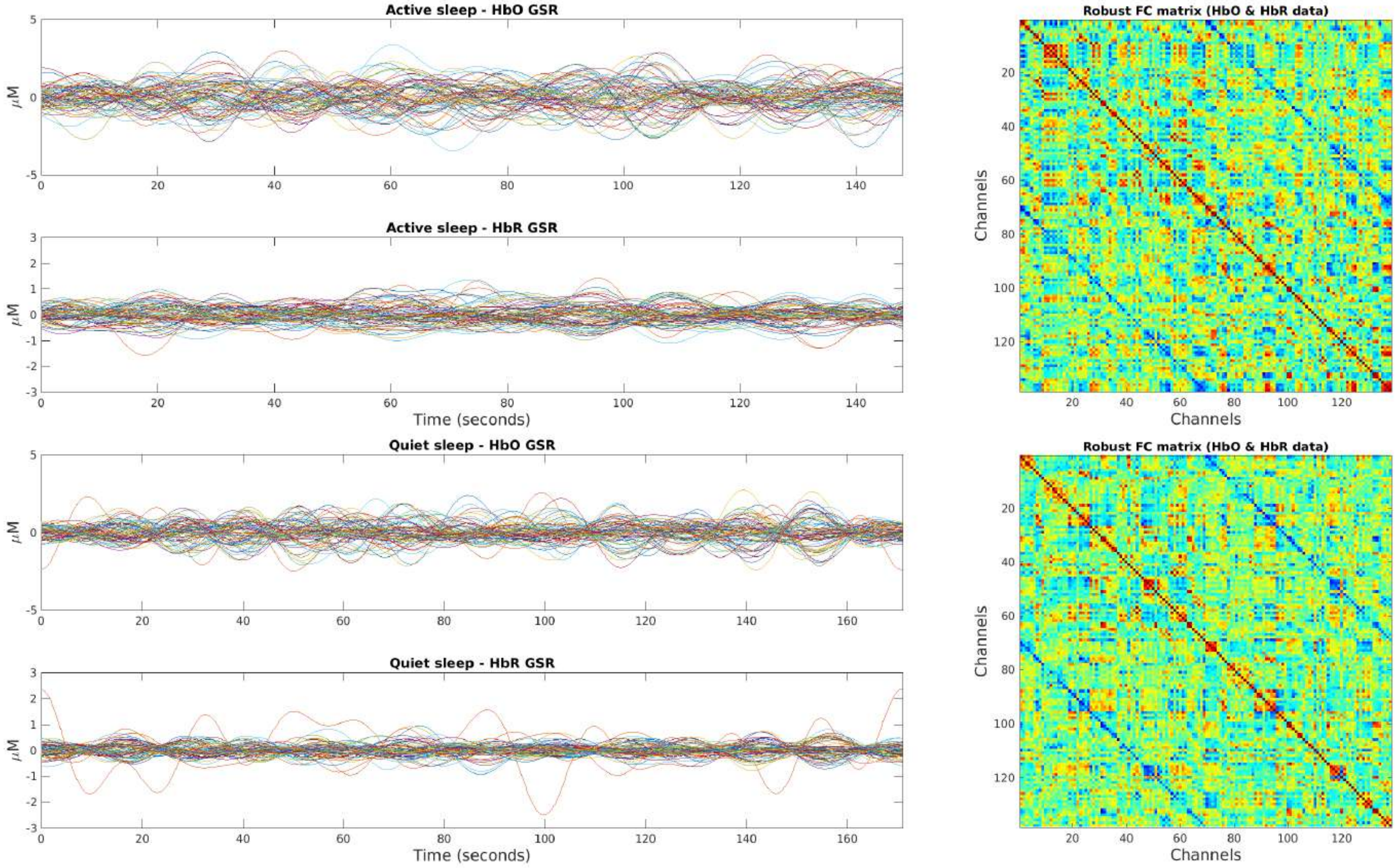
Active sleep - HbO GSR
Active sleep - HbR GSR
Quiet sleep - HbO GSR
Quiet sleep - HbR GSR
μM
Time (seconds)
Robust FC matrix (HbO & HbR data)
Robust FC matrix (HbO & HbR data)
Channels
Channels

HT_021

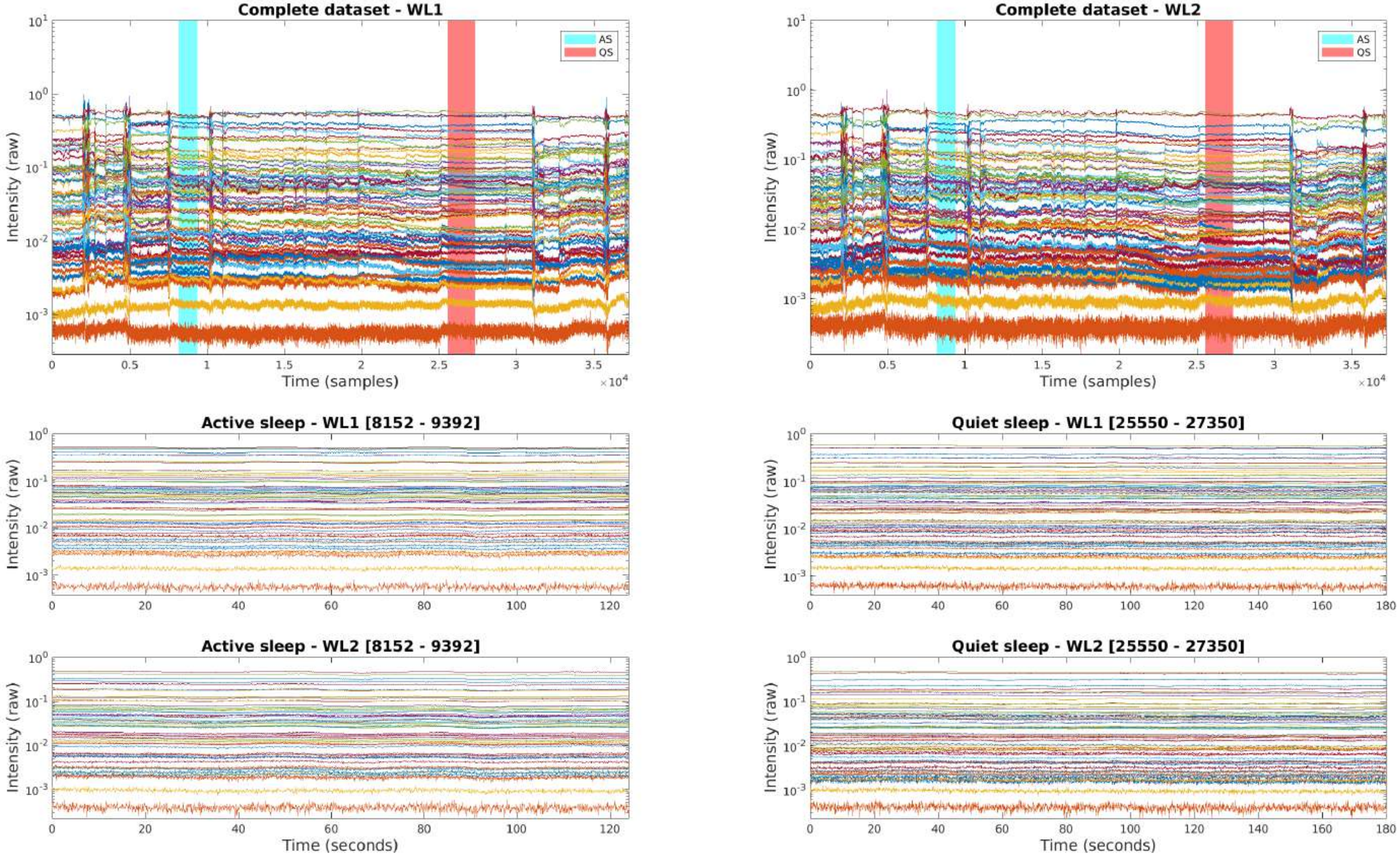

Complete dataset - WL1
Complete dataset - WL2
AS
QS
Intensity (raw)
Time (samples)
×10^4
Active sleep - WL1 [8152 - 9392]
Quiet sleep - WL1 [25550 - 27350]
Active sleep - WL2 [8152 - 9392]
Quiet sleep - WL2 [25550 - 27350]
Time (seconds)

HT_021

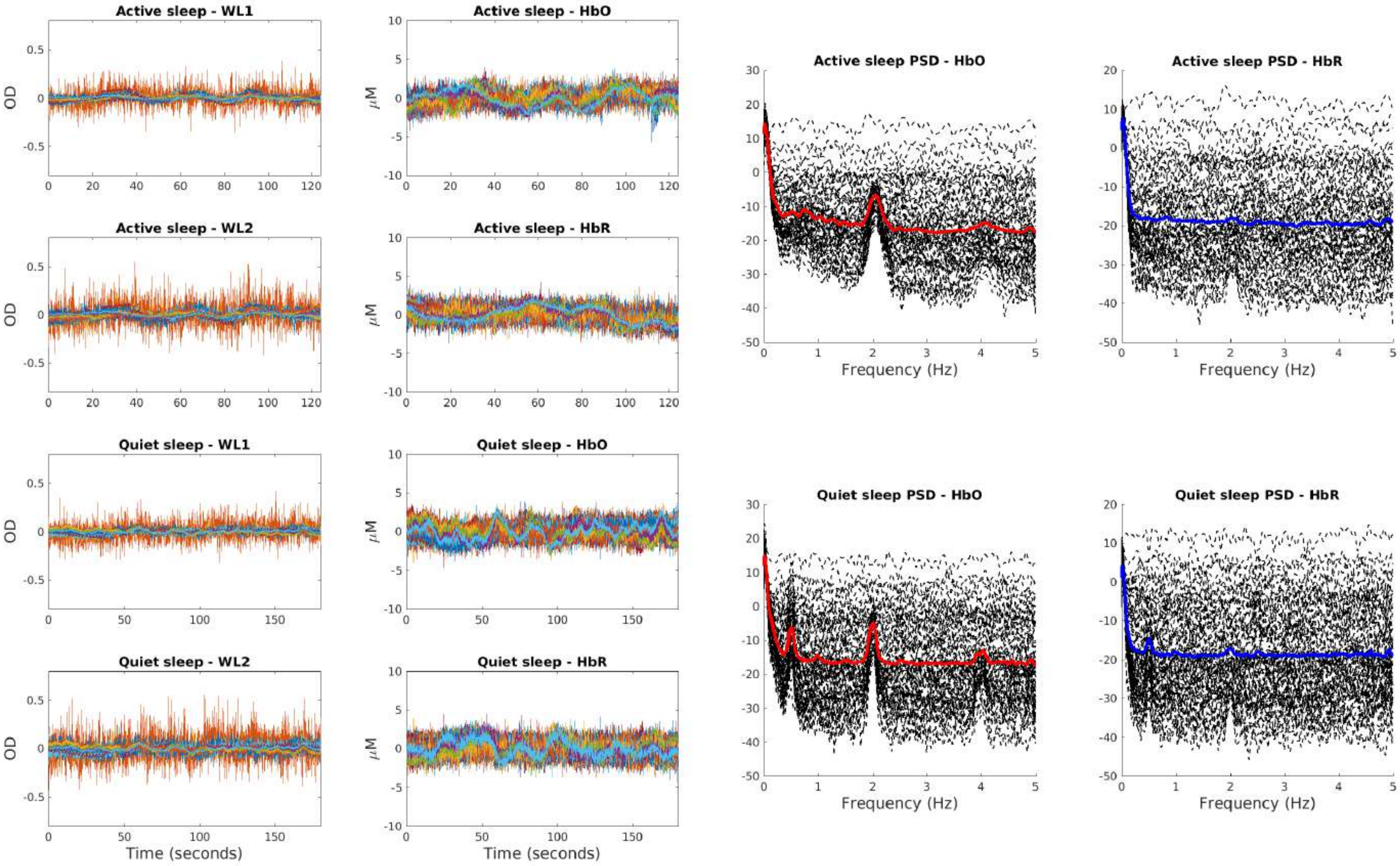

Active sleep - WL1
Active sleep - HbO
Active sleep PSD - HbO
Active sleep PSD - HbR
Active sleep - WL2
Active sleep - HbR
Quiet sleep - WL1
Quiet sleep - HbO
Quiet sleep PSD - HbO
Quiet sleep PSD - HbR
Quiet sleep - WL2
Quiet sleep - HbR
OD
μM
Time (seconds)
Frequency (Hz)

HT_021

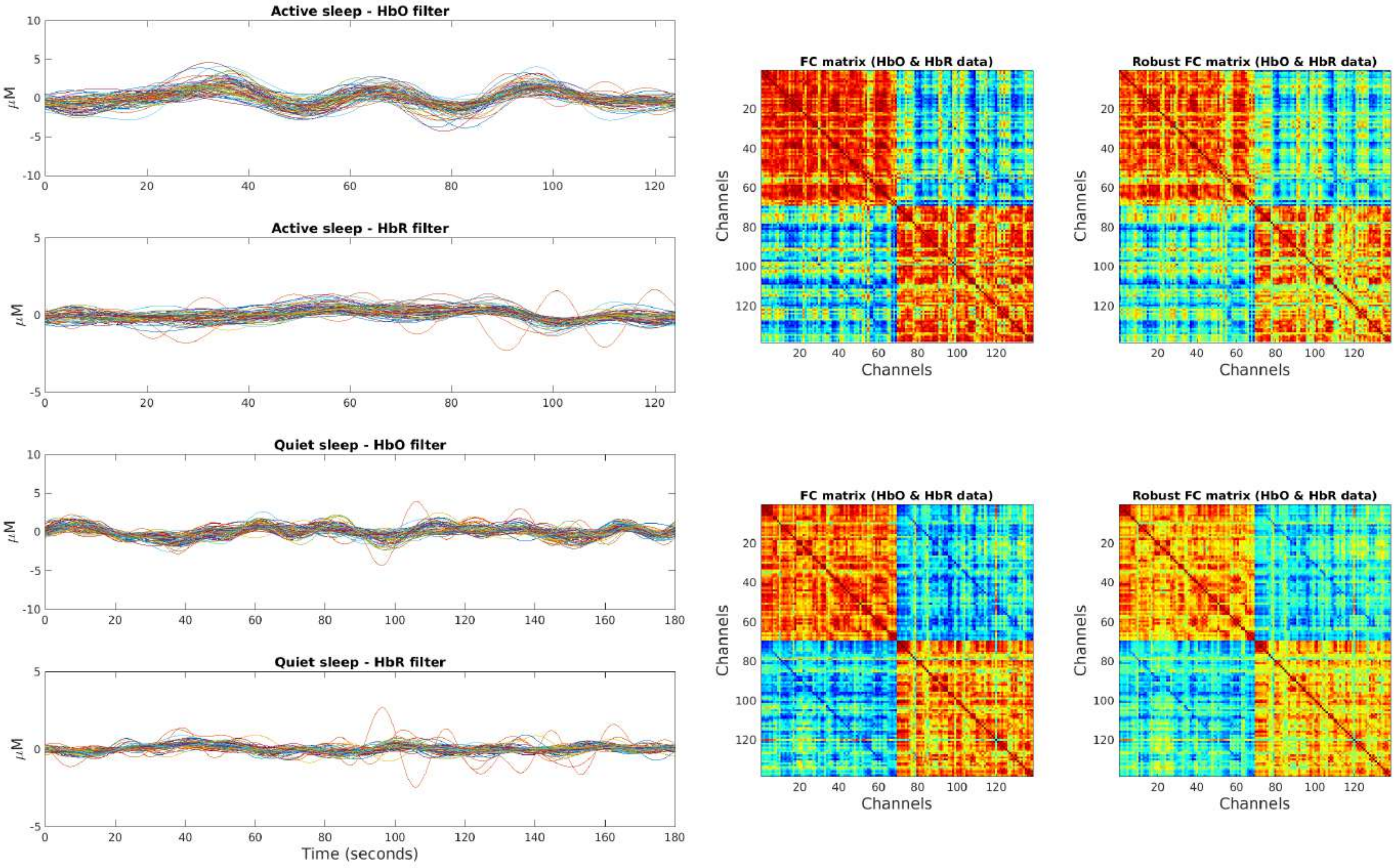
Active sleep - HbO filter
Active sleep - HbR filter
Quiet sleep - HbO filter
Quiet sleep - HbR filter
μM
Time (seconds)
FC matrix (HbO & HbR data)
Robust FC matrix (HbO & HbR data)
Channels

HT_021

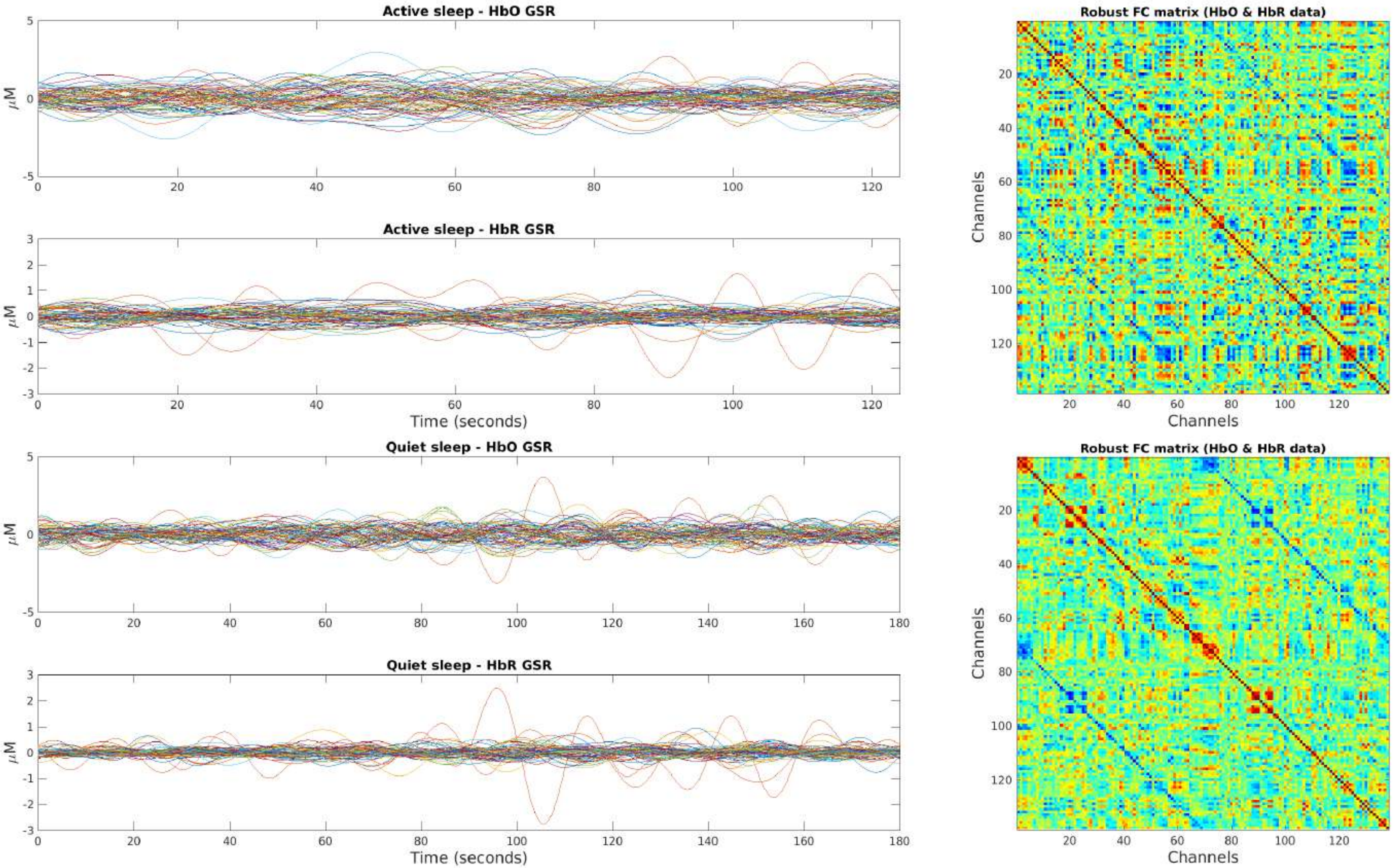

Active sleep - HbO GSR
Active sleep - HbR GSR
Quiet sleep - HbO GSR
Quiet sleep - HbR GSR
μM
Time (seconds)
Robust FC matrix (HbO & HbR data)
Channels

HT_022

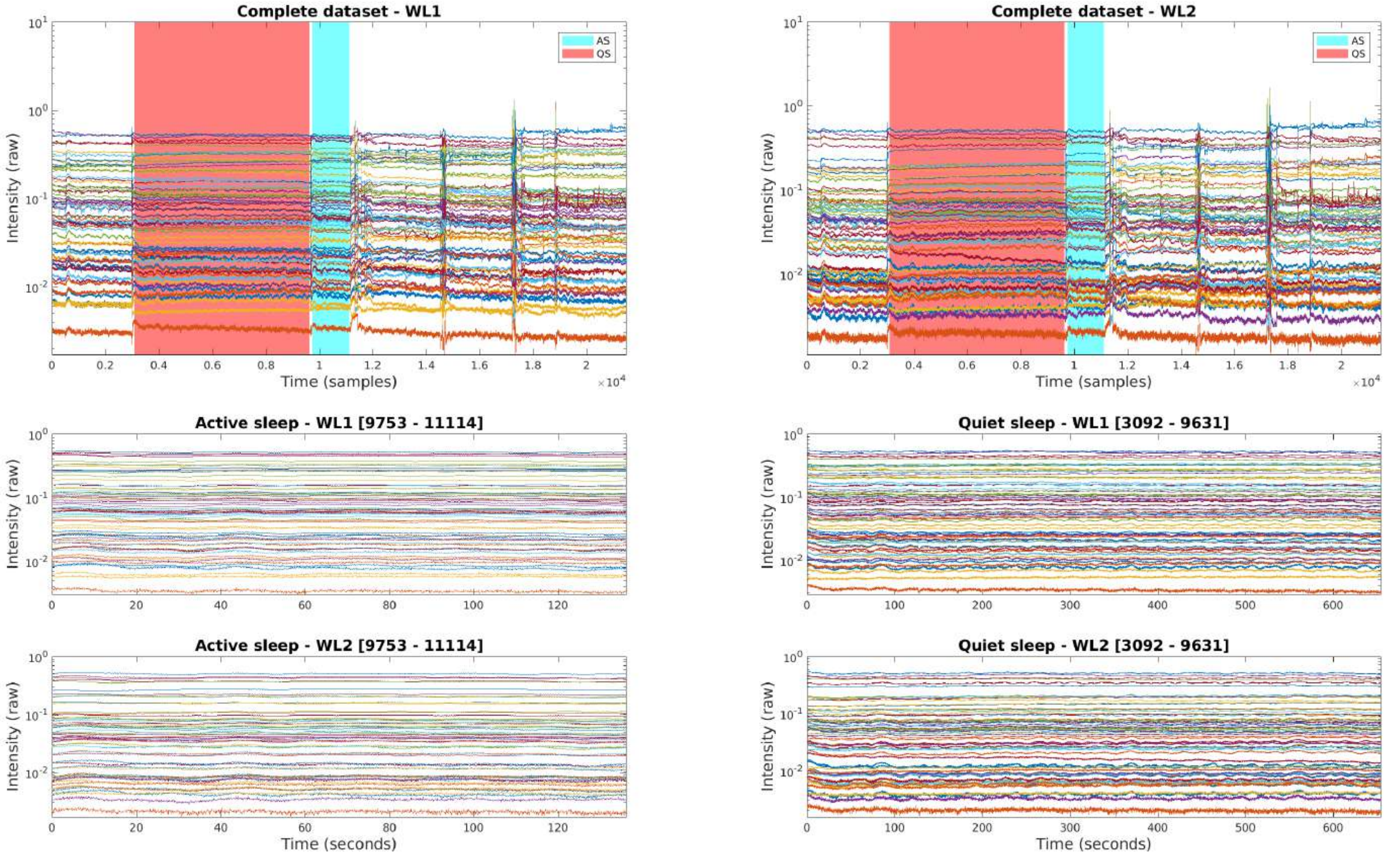
Complete dataset - WL1
Complete dataset - WL2
AS
QS
Intensity (raw)
Time (samples)
×10^4
Active sleep - WL1 [9753 - 11114]
Quiet sleep - WL1 [3092 - 9631]
Active sleep - WL2 [9753 - 11114]
Quiet sleep - WL2 [3092 - 9631]
Time (seconds)

HT_022

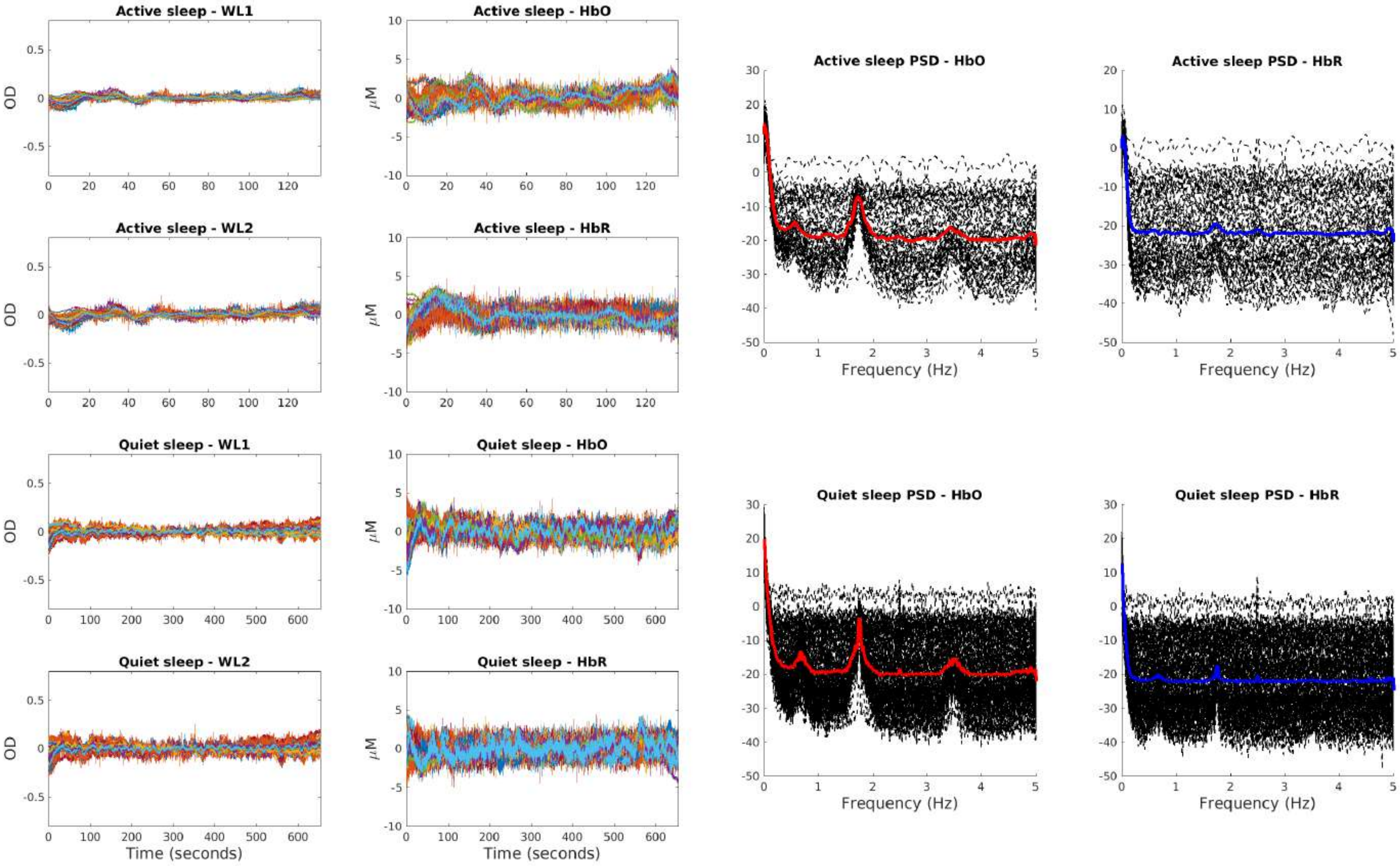

Active sleep - WL1
Active sleep - WL2
Quiet sleep - WL1
Quiet sleep - WL2
OD
Active sleep - HbO
Active sleep - HbR
Quiet sleep - HbO
Quiet sleep - HbR
μM
Time (seconds)
Active sleep PSD - HbO
Active sleep PSD - HbR
Quiet sleep PSD - HbO
Quiet sleep PSD - HbR
Frequency (Hz)

HT_022

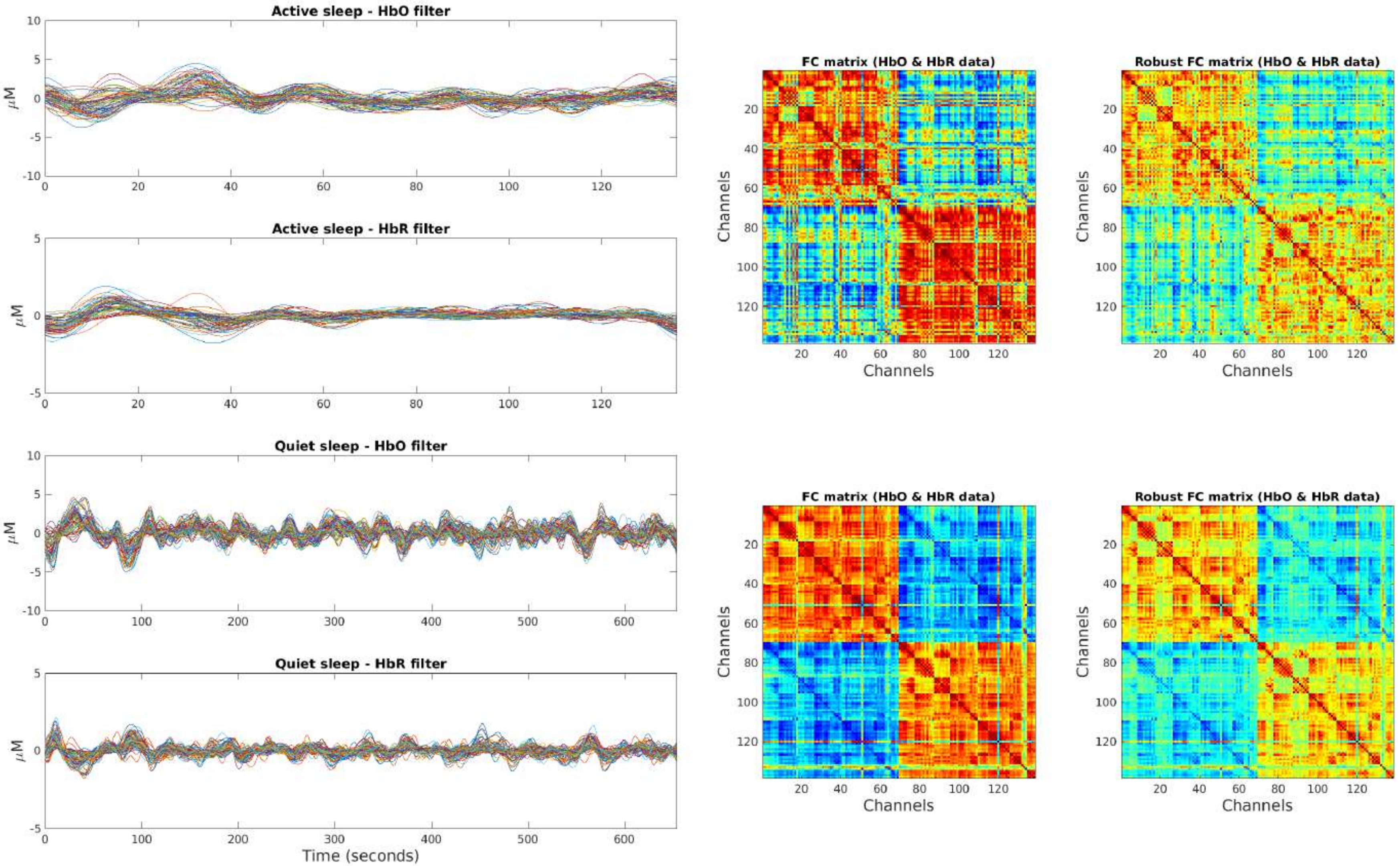

Active sleep - HbO filter
Active sleep - HbR filter
Quiet sleep - HbO filter
Quiet sleep - HbR filter
μM
Time (seconds)
FC matrix (HbO & HbR data)
Robust FC matrix (HbO & HbR data)
Channels

HT_022

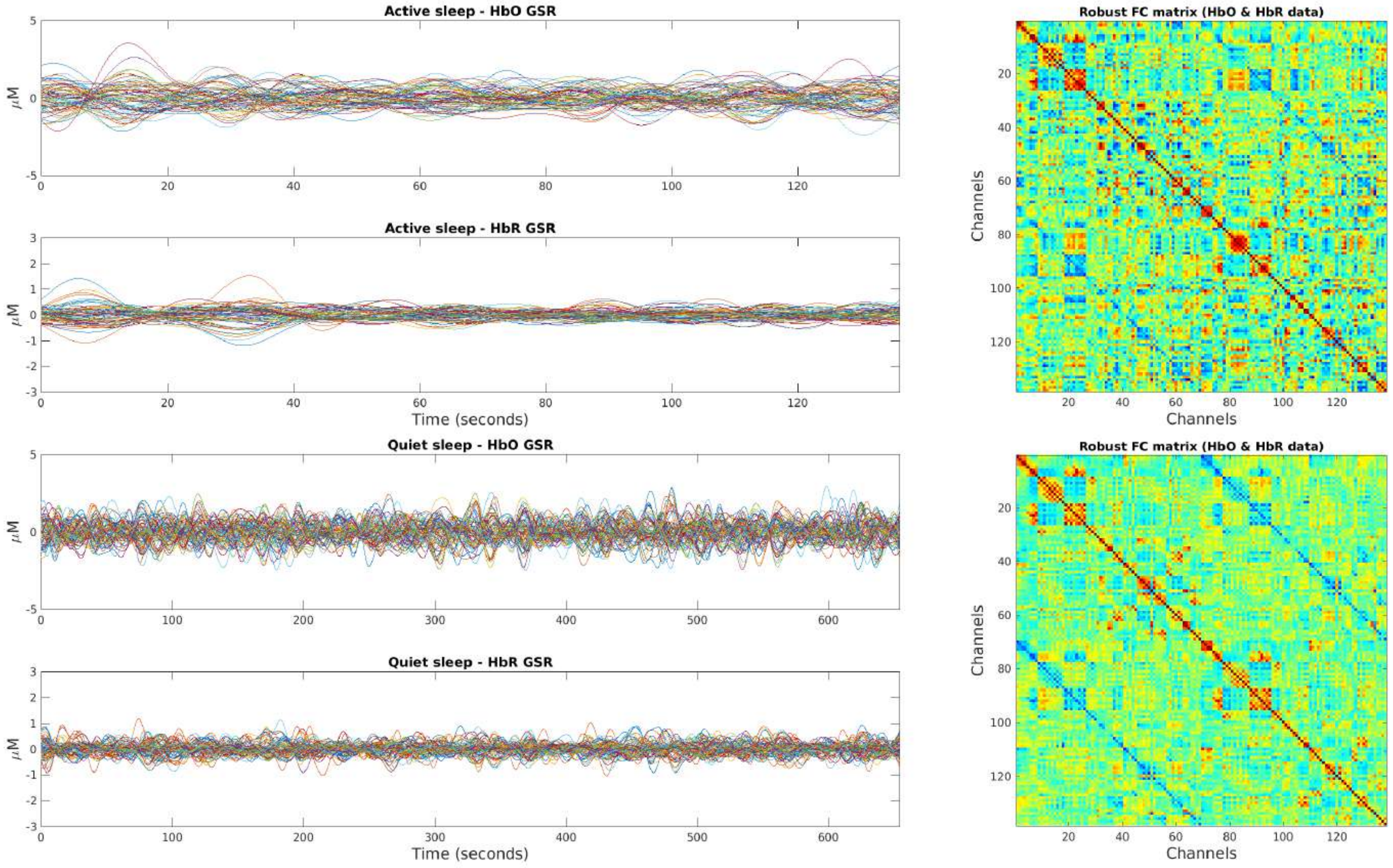

Active sleep - HbO GSR
Active sleep - HbR GSR
Quiet sleep - HbO GSR
Quiet sleep - HbR GSR
μM
Time (seconds)
Robust FC matrix (HbO & HbR data)
Channels

HT_023

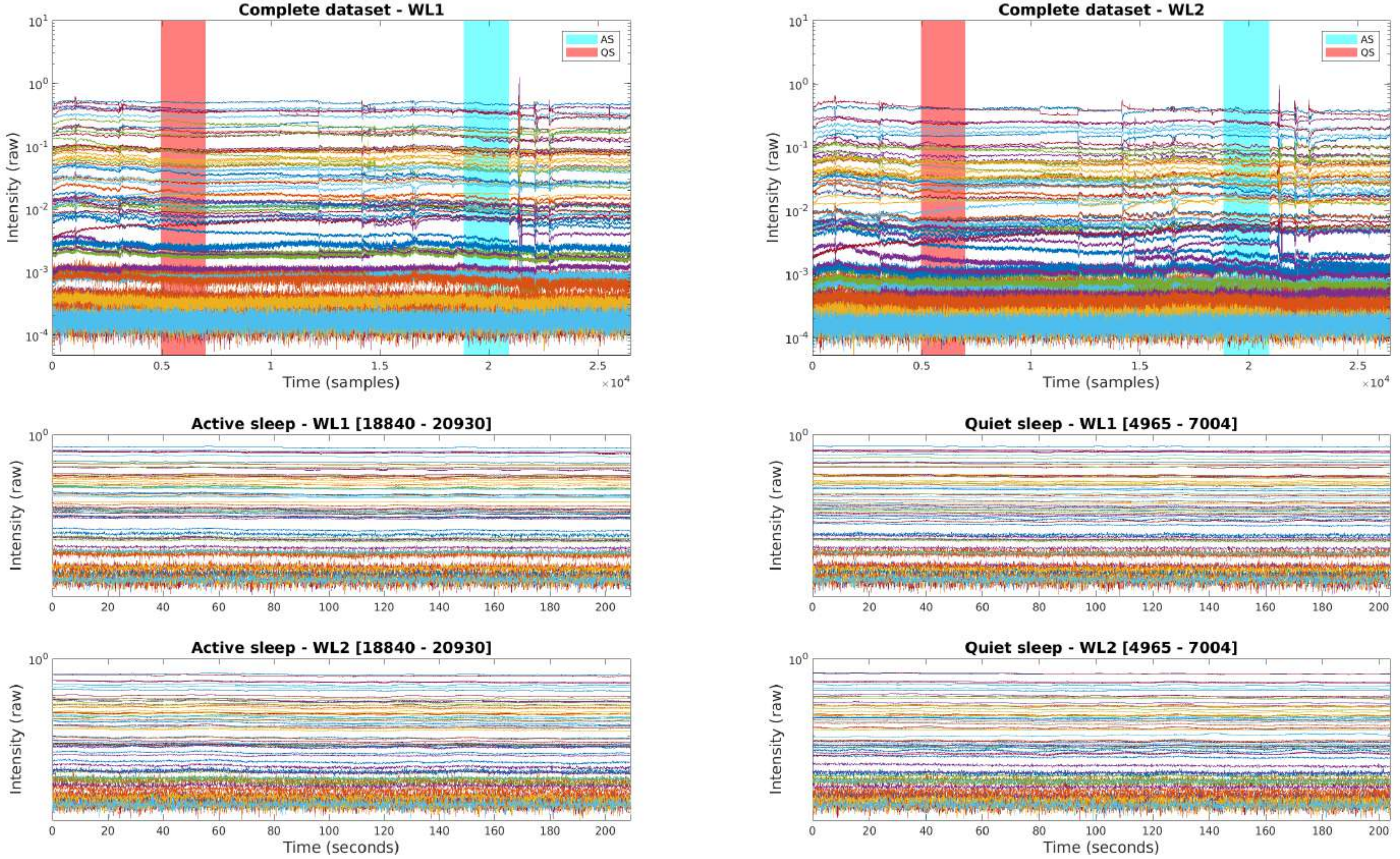

Complete dataset - WL1
Complete dataset - WL2
AS
QS
Intensity (raw)
Time (samples)
×10^4
Active sleep - WL1 [18840 - 20930]
Quiet sleep - WL1 [4965 - 7004]
Active sleep - WL2 [18840 - 20930]
Quiet sleep - WL2 [4965 - 7004]
Time (seconds)

HT_023

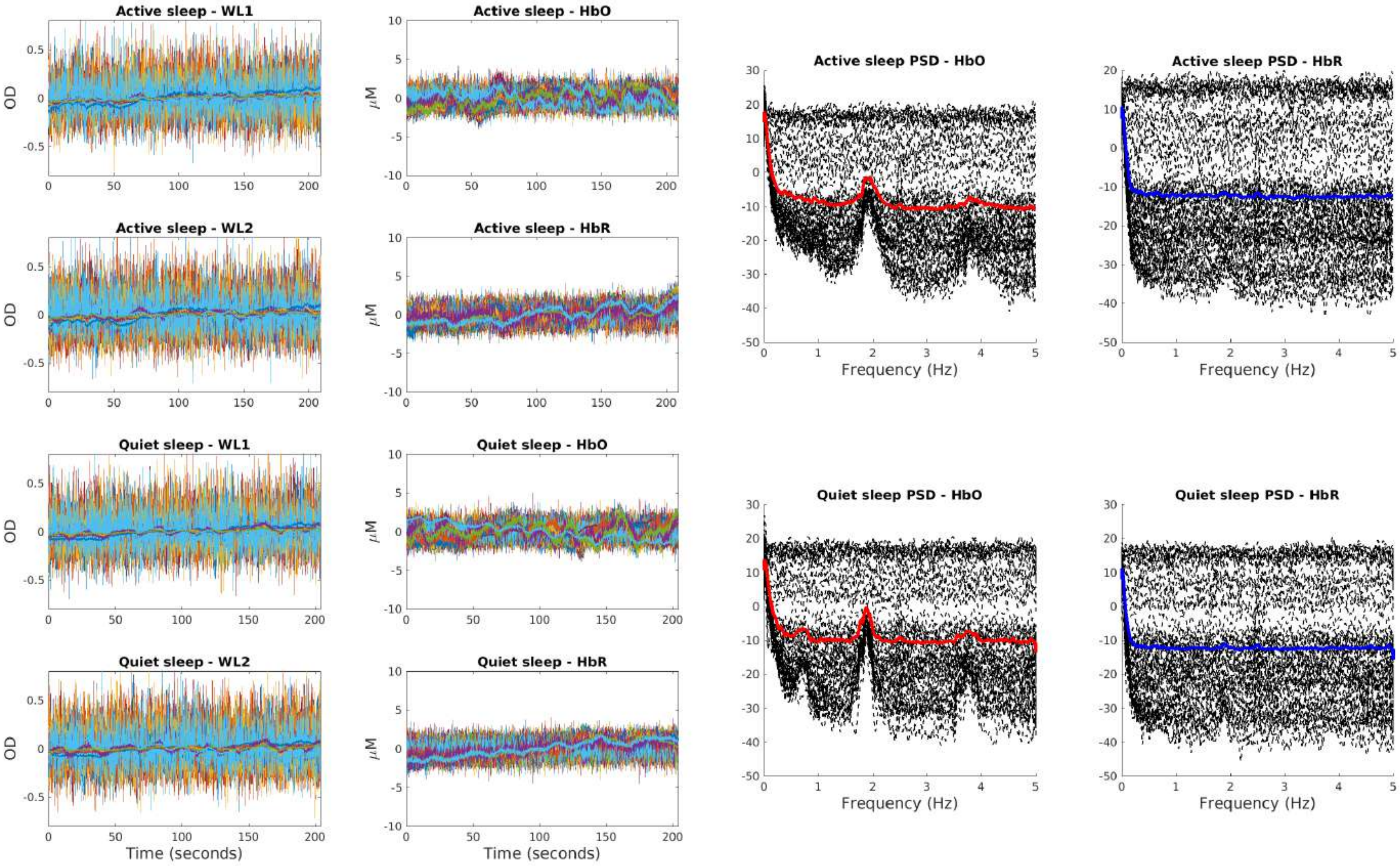

Active sleep - WL1
Active sleep - HbO
Active sleep PSD - HbO
Active sleep PSD - HbR
Active sleep - WL2
Active sleep - HbR
Quiet sleep - WL1
Quiet sleep - HbO
Quiet sleep PSD - HbO
Quiet sleep PSD - HbR
Quiet sleep - WL2
Quiet sleep - HbR
OD
μM
Time (seconds)
Frequency (Hz)

HT_023

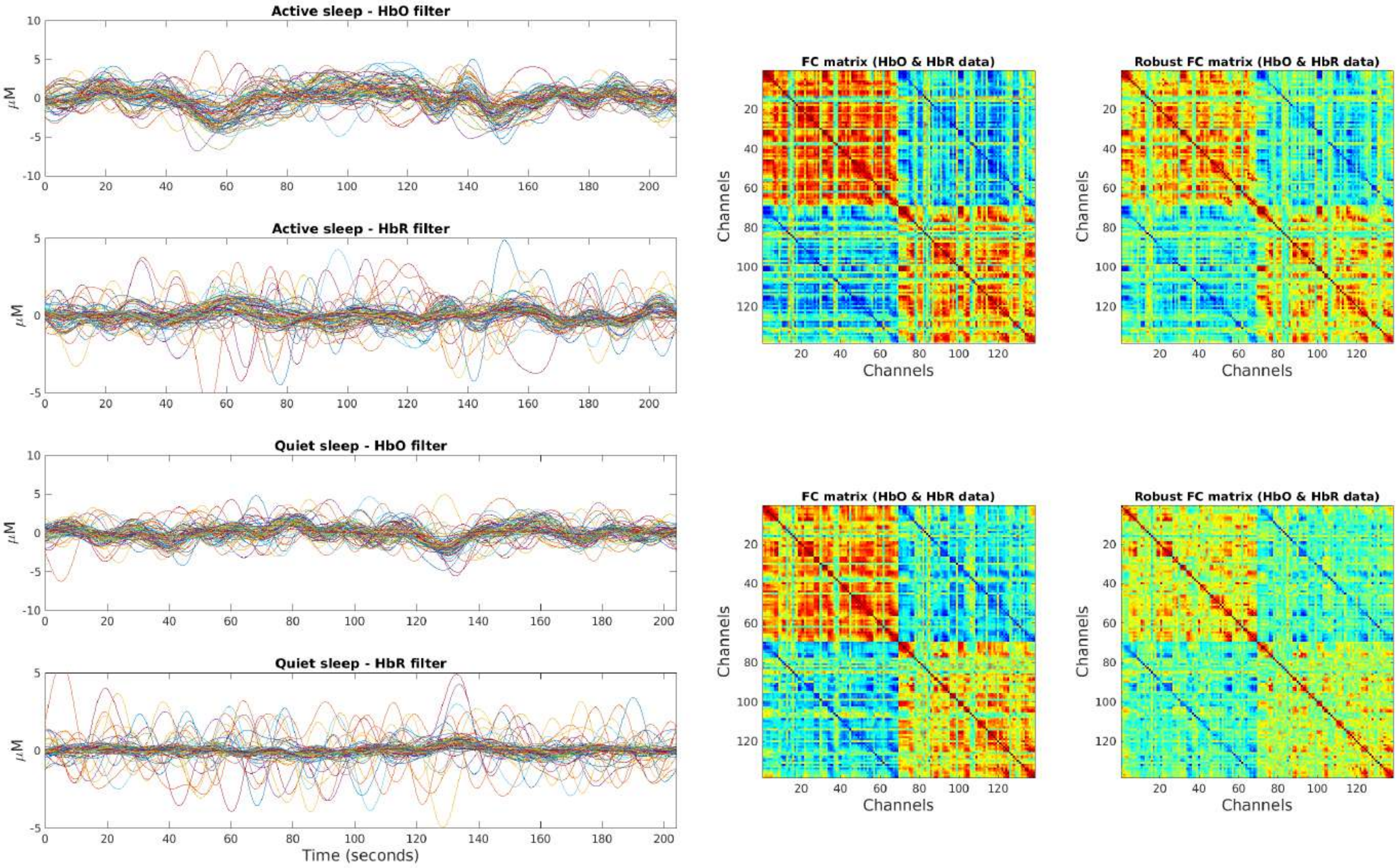
Active sleep - HbO filter
Active sleep - HbR filter
Quiet sleep - HbO filter
Quiet sleep - HbR filter
μM
Time (seconds)
FC matrix (HbO & HbR data)
Robust FC matrix (HbO & HbR data)
Channels

HT_023

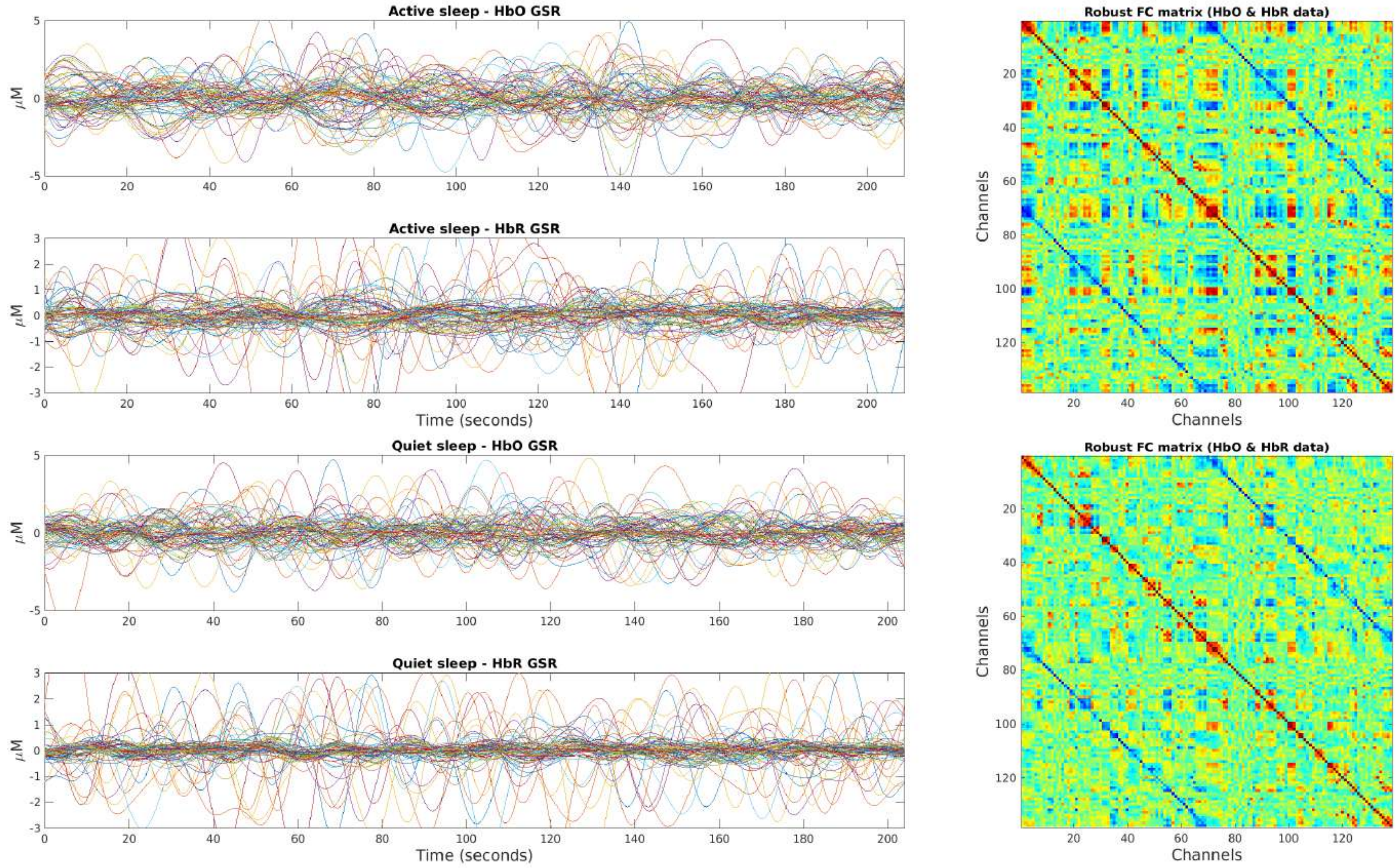

Active sleep - HbO GSR
Active sleep - HbR GSR
Quiet sleep - HbO GSR
Quiet sleep - HbR GSR
μM
Time (seconds)
Robust FC matrix (HbO & HbR data)
Channels

HT_024

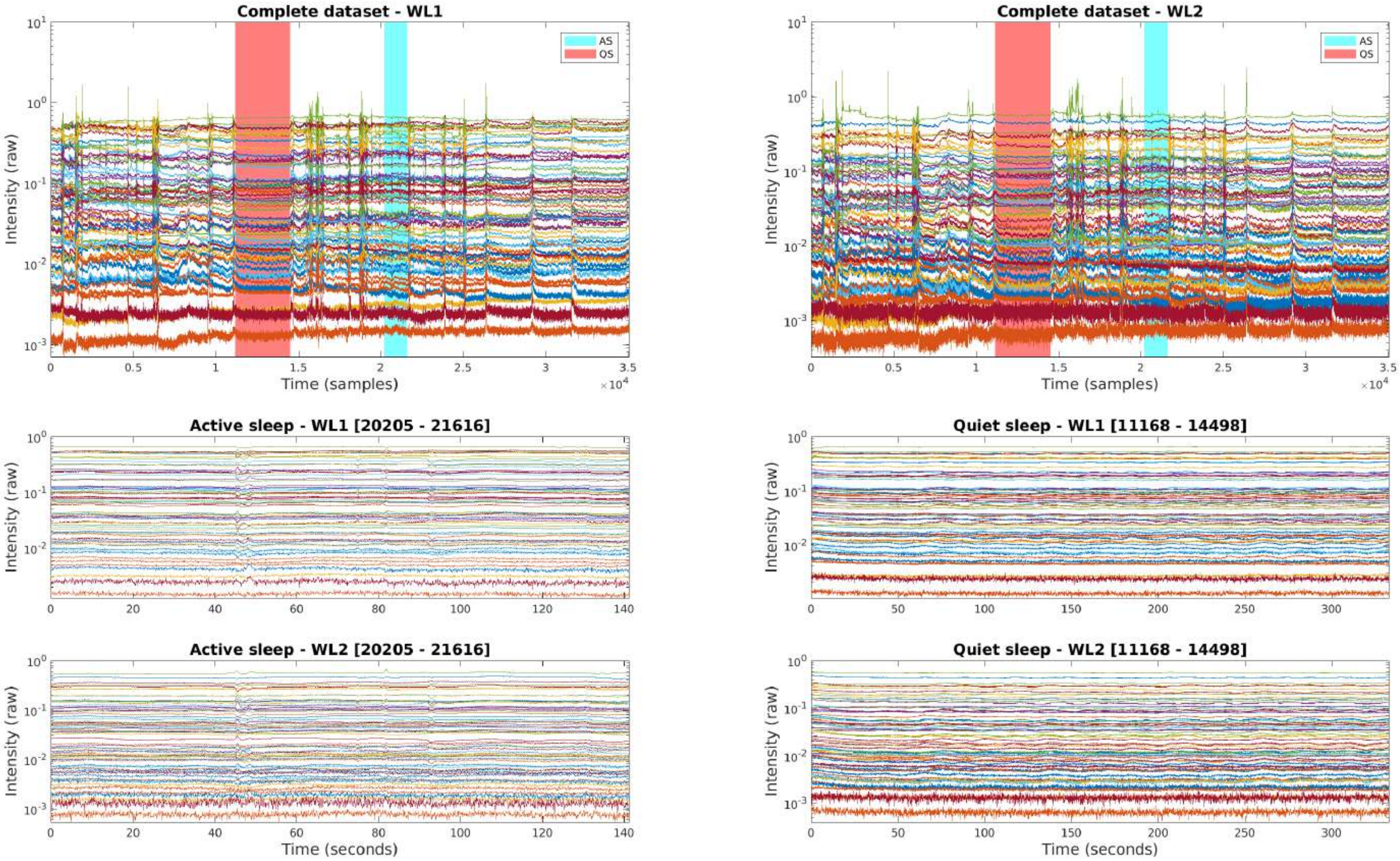

Complete dataset - WL1
Complete dataset - WL2
AS
QS
Intensity (raw)
Time (samples)
Active sleep - WL1 [20205 - 21616]
Quiet sleep - WL1 [11168 - 14498]
Active sleep - WL2 [20205 - 21616]
Quiet sleep - WL2 [11168 - 14498]
Time (seconds)

HT_024

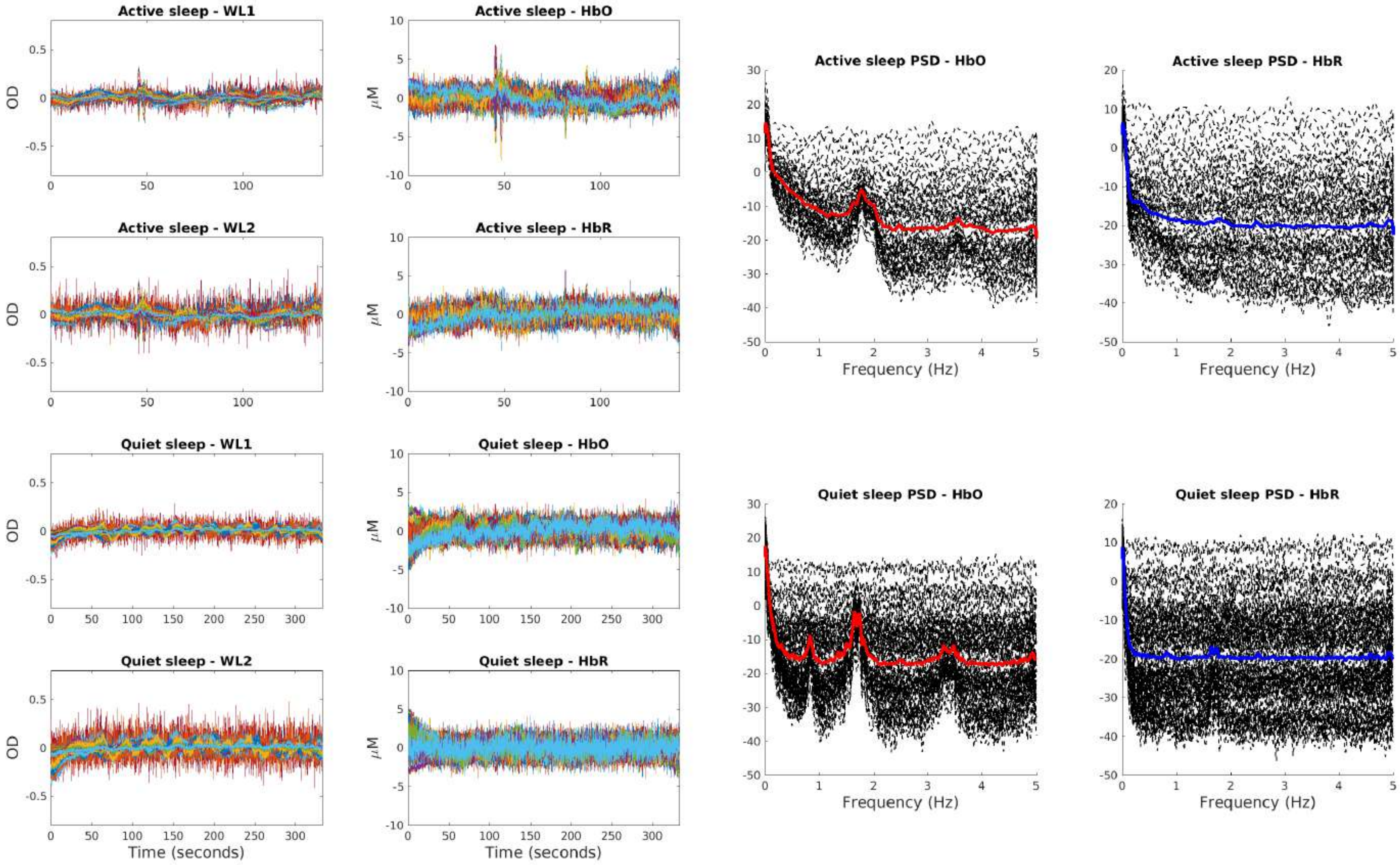

Active sleep - WL1
Active sleep - HbO
Active sleep PSD - HbO
Active sleep PSD - HbR
Active sleep - WL2
Active sleep - HbR
Quiet sleep - WL1
Quiet sleep - HbO
Quiet sleep PSD - HbO
Quiet sleep PSD - HbR
Quiet sleep - WL2
Quiet sleep - HbR
OD
μM
Frequency (Hz)
Time (seconds)

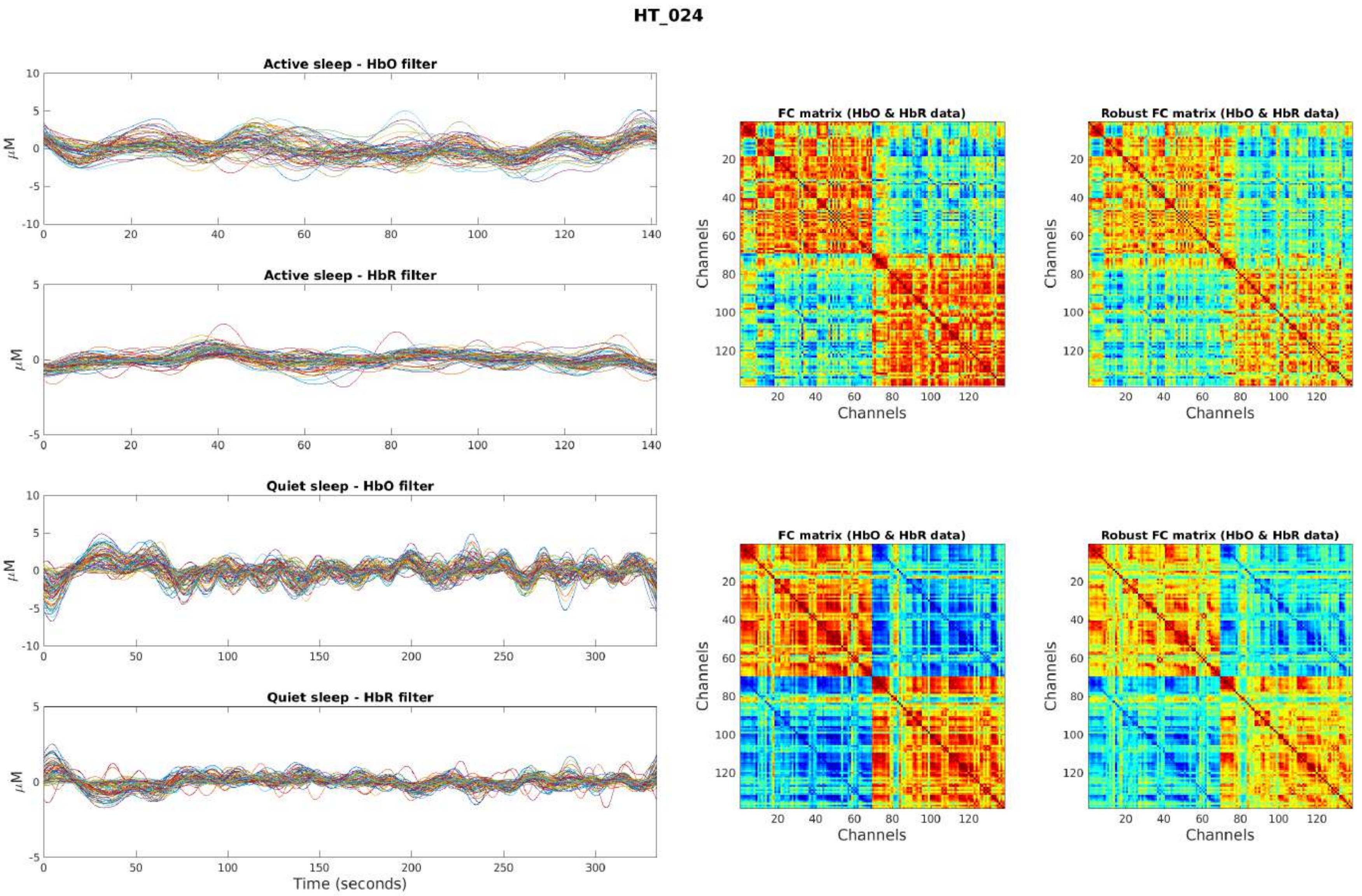
HT_024
Active sleep - HbO filter
Active sleep - HbR filter
Quiet sleep - HbO filter
Quiet sleep - HbR filter
μM
Time (seconds)
FC matrix (HbO & HbR data)
Robust FC matrix (HbO & HbR data)
Channels

HT_024

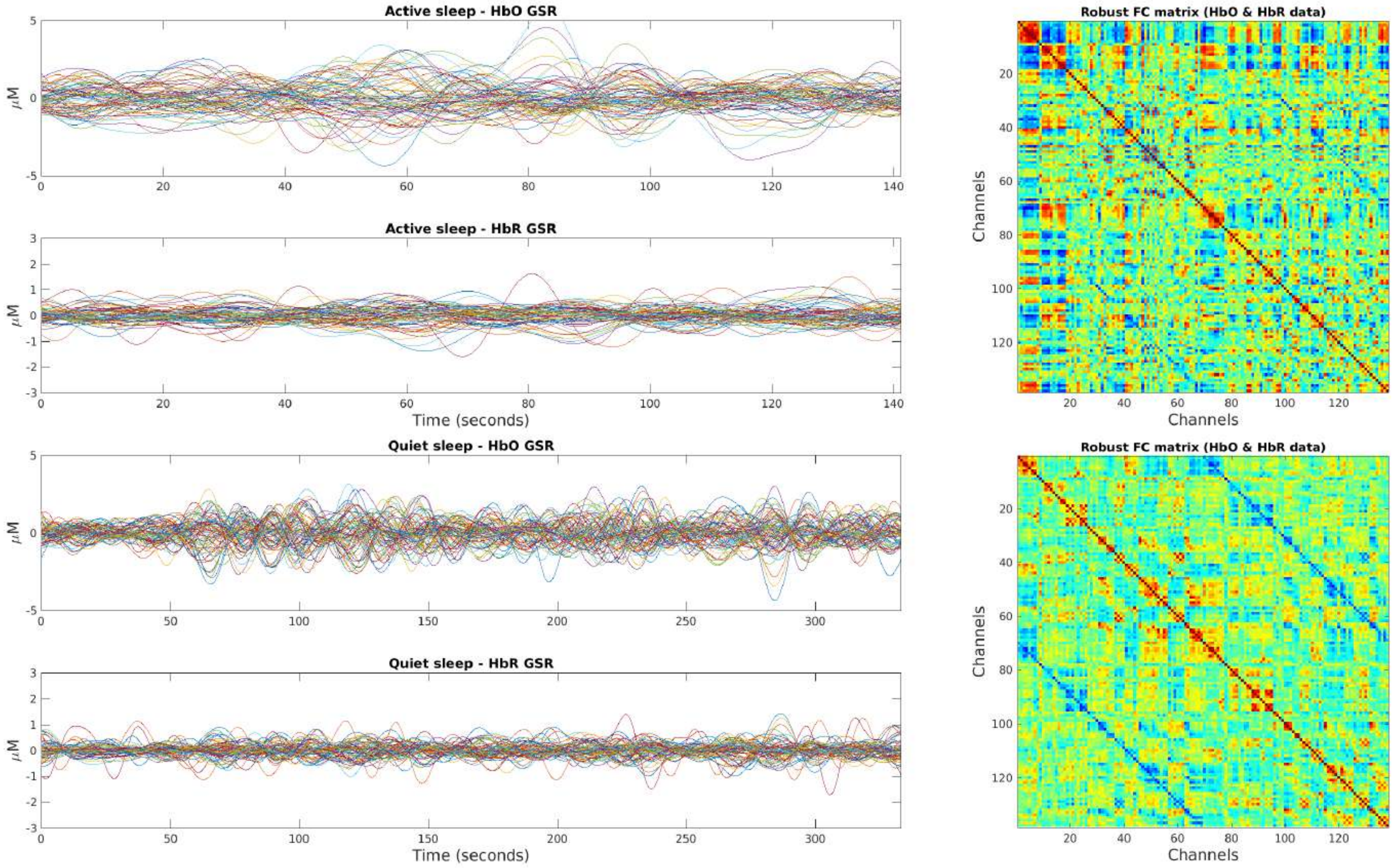

Active sleep - HbO GSR
Active sleep - HbR GSR
Quiet sleep - HbO GSR
Quiet sleep - HbR GSR
μM
Time (seconds)
Robust FC matrix (HbO & HbR data)
Robust FC matrix (HbO & HbR data)
Channels
Channels

HT_025

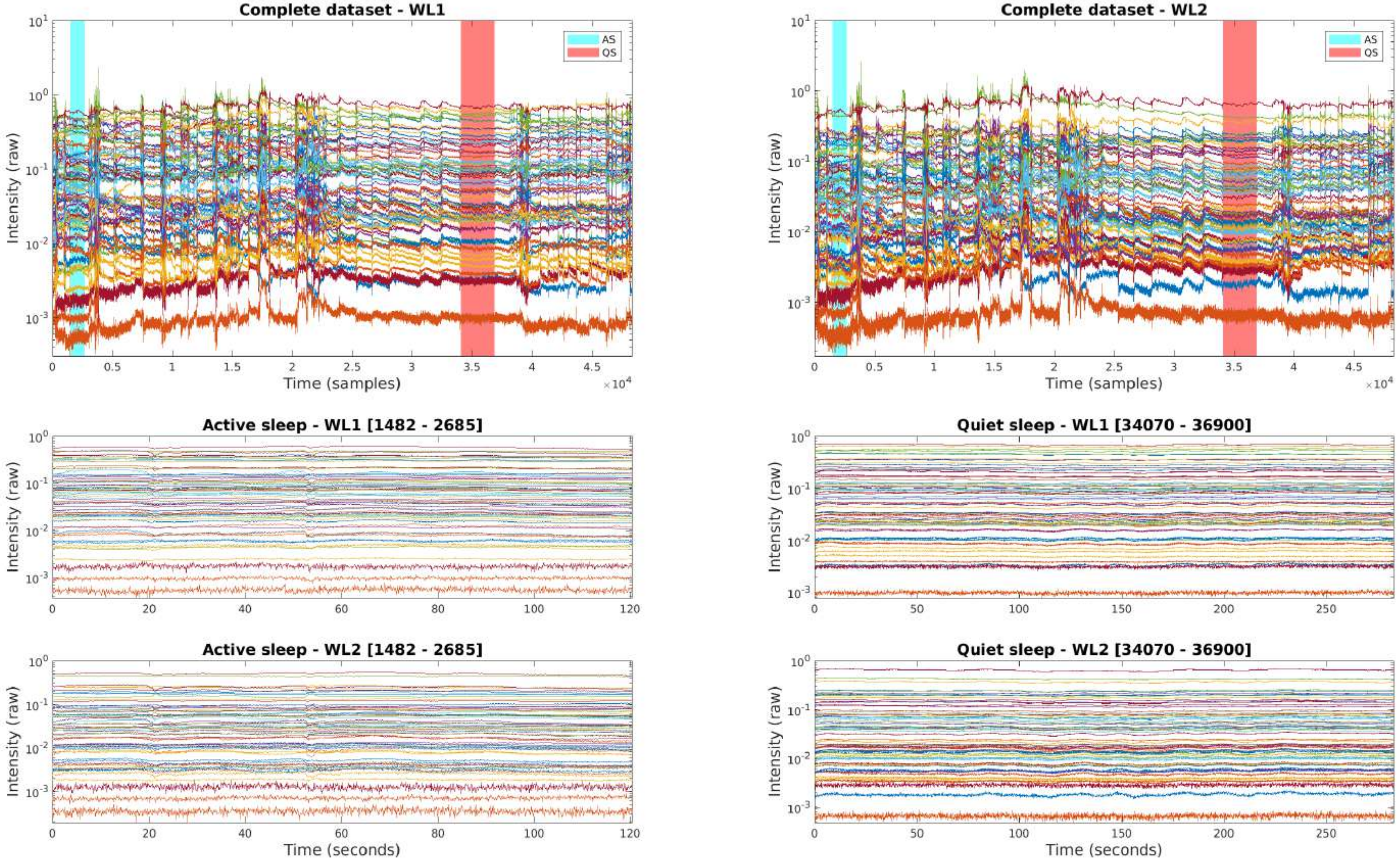

Complete dataset - WL1
Complete dataset - WL2
AS
QS
Intensity (raw)
Time (samples)
×10^4
Active sleep - WL1 [1482 - 2685]
Quiet sleep - WL1 [34070 - 36900]
Active sleep - WL2 [1482 - 2685]
Quiet sleep - WL2 [34070 - 36900]
Time (seconds)

HT_025

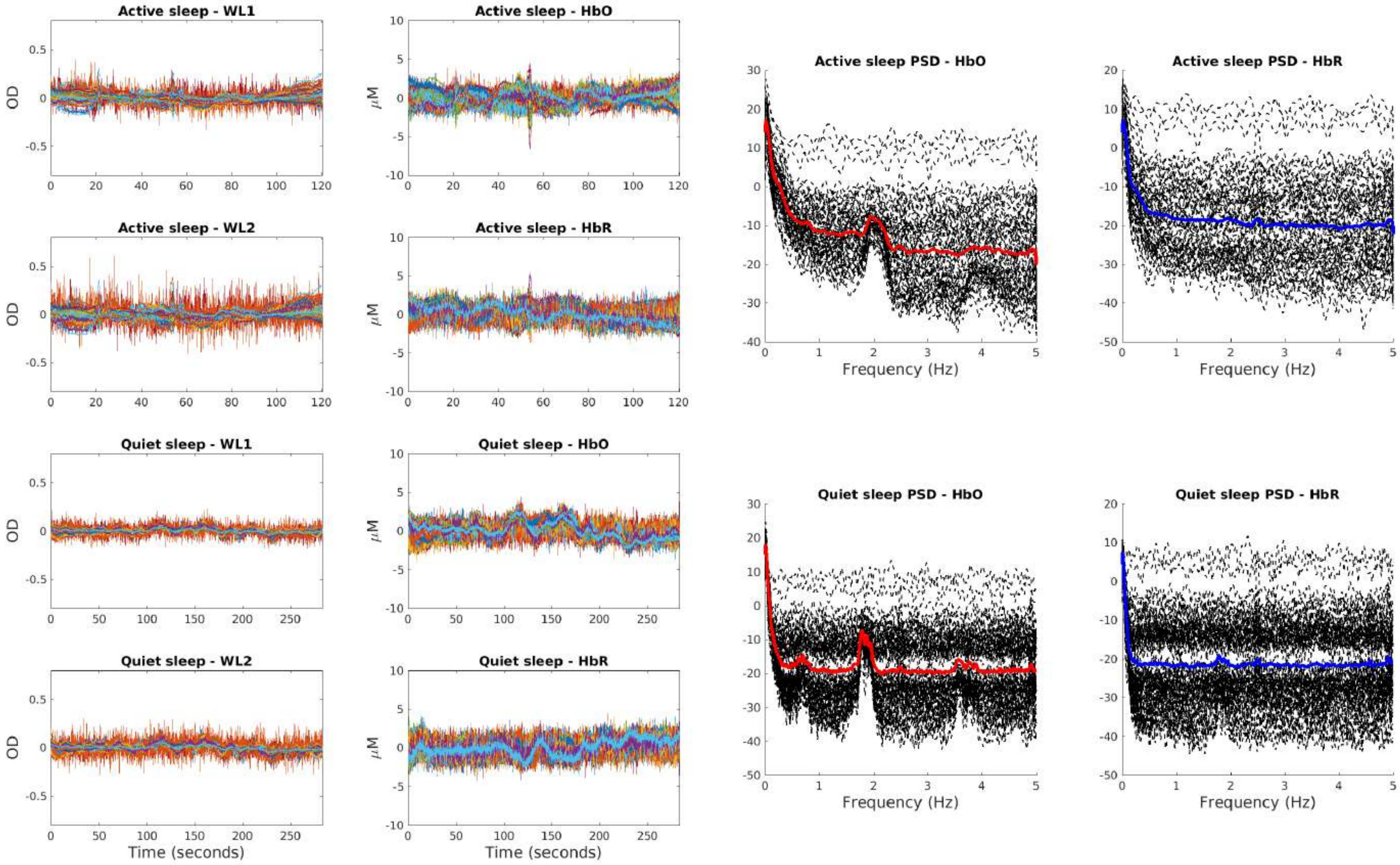

Active sleep - WL1
Active sleep - WL2
Quiet sleep - WL1
Quiet sleep - WL2
Active sleep - HbO
Active sleep - HbR
Quiet sleep - HbO
Quiet sleep - HbR
Active sleep PSD - HbO
Active sleep PSD - HbR
Quiet sleep PSD - HbO
Quiet sleep PSD - HbR
OD
μM
Time (seconds)
Frequency (Hz)

HT_025

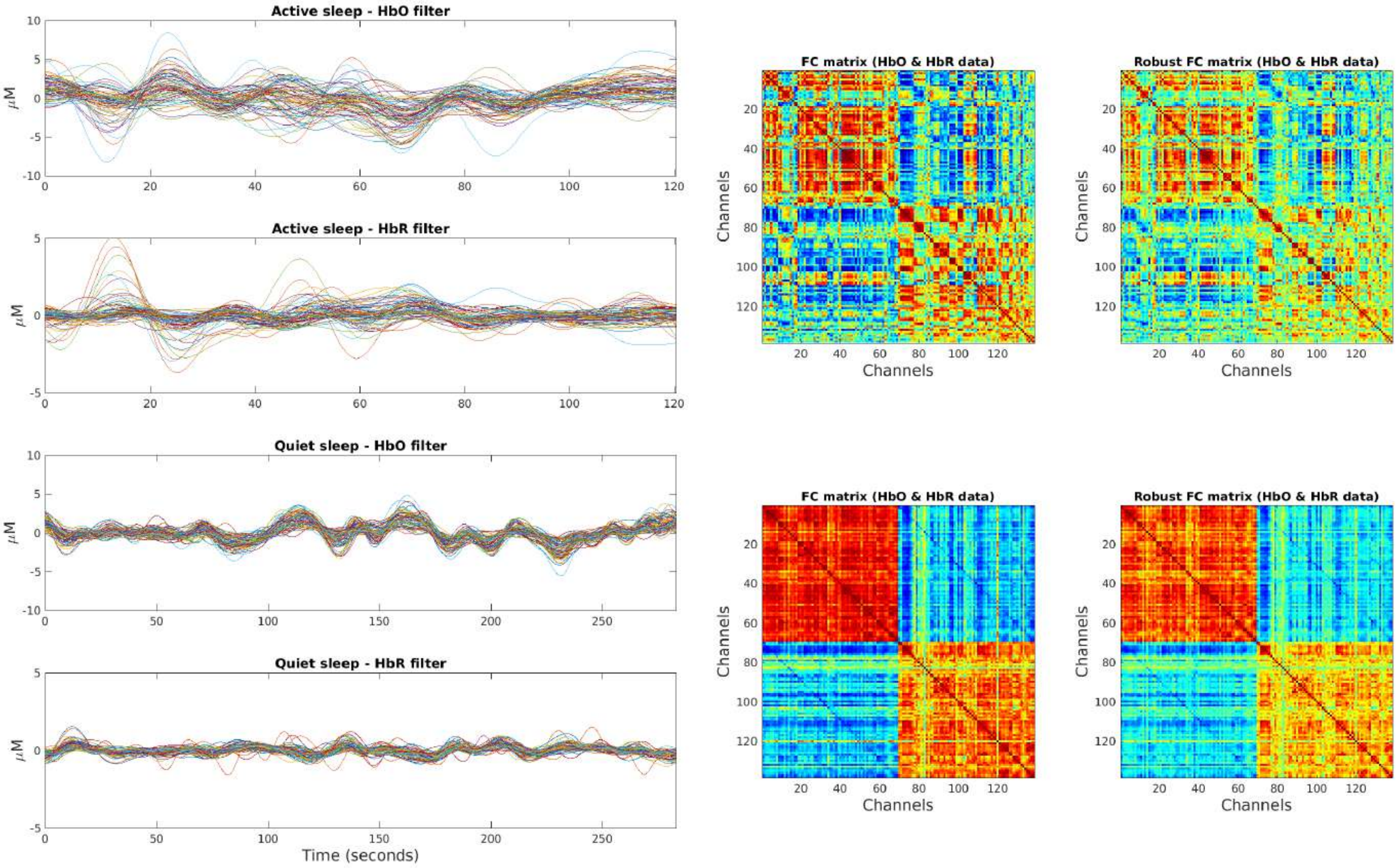

Active sleep - HbO filter
Active sleep - HbR filter
Quiet sleep - HbO filter
Quiet sleep - HbR filter
μM
Time (seconds)
FC matrix (HbO & HbR data)
Robust FC matrix (HbO & HbR data)
Channels

HT_025

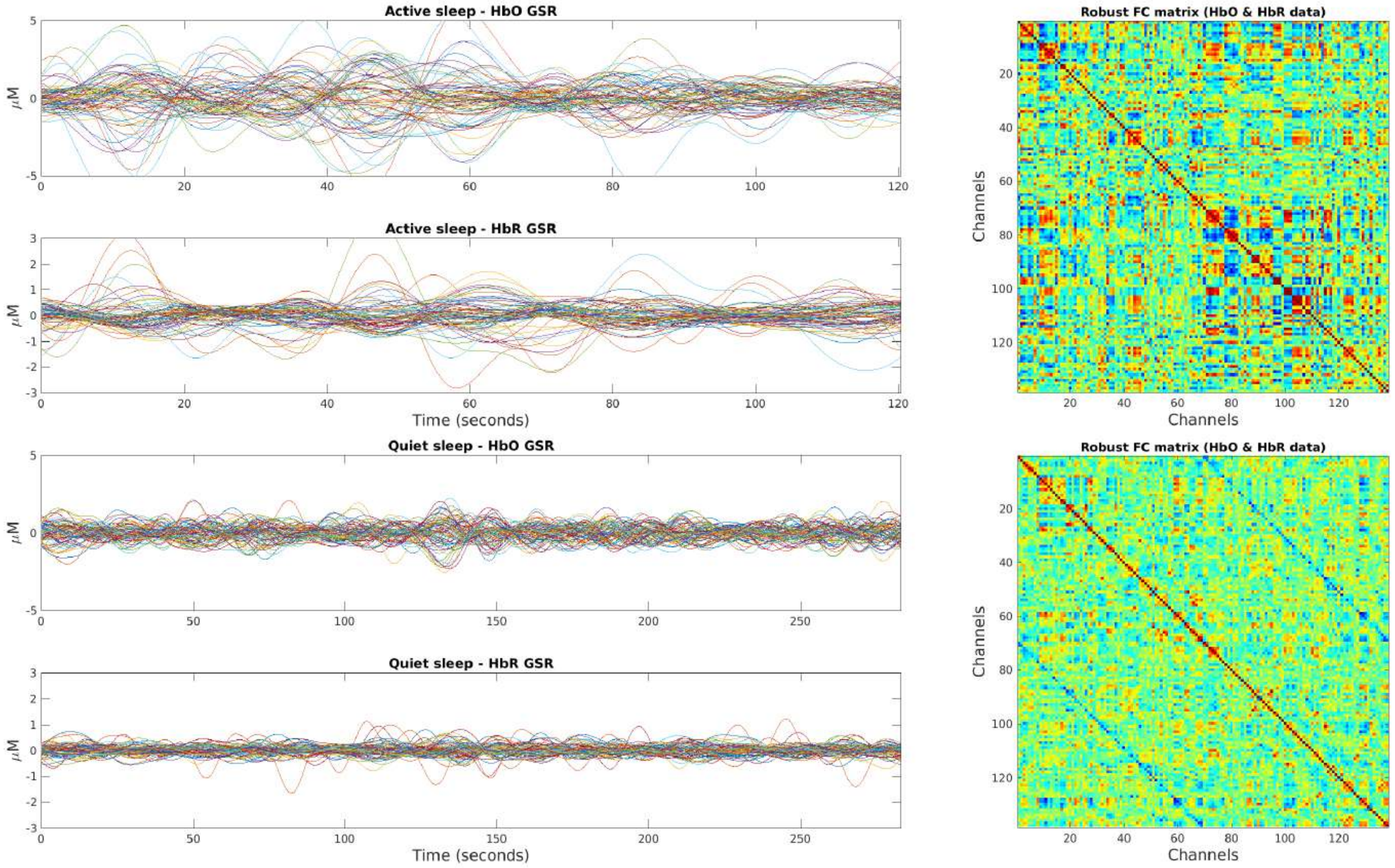

Active sleep - HbO GSR
Active sleep - HbR GSR
Quiet sleep - HbO GSR
Quiet sleep - HbR GSR
μM
Time (seconds)
Robust FC matrix (HbO & HbR data)
Channels

HT_026

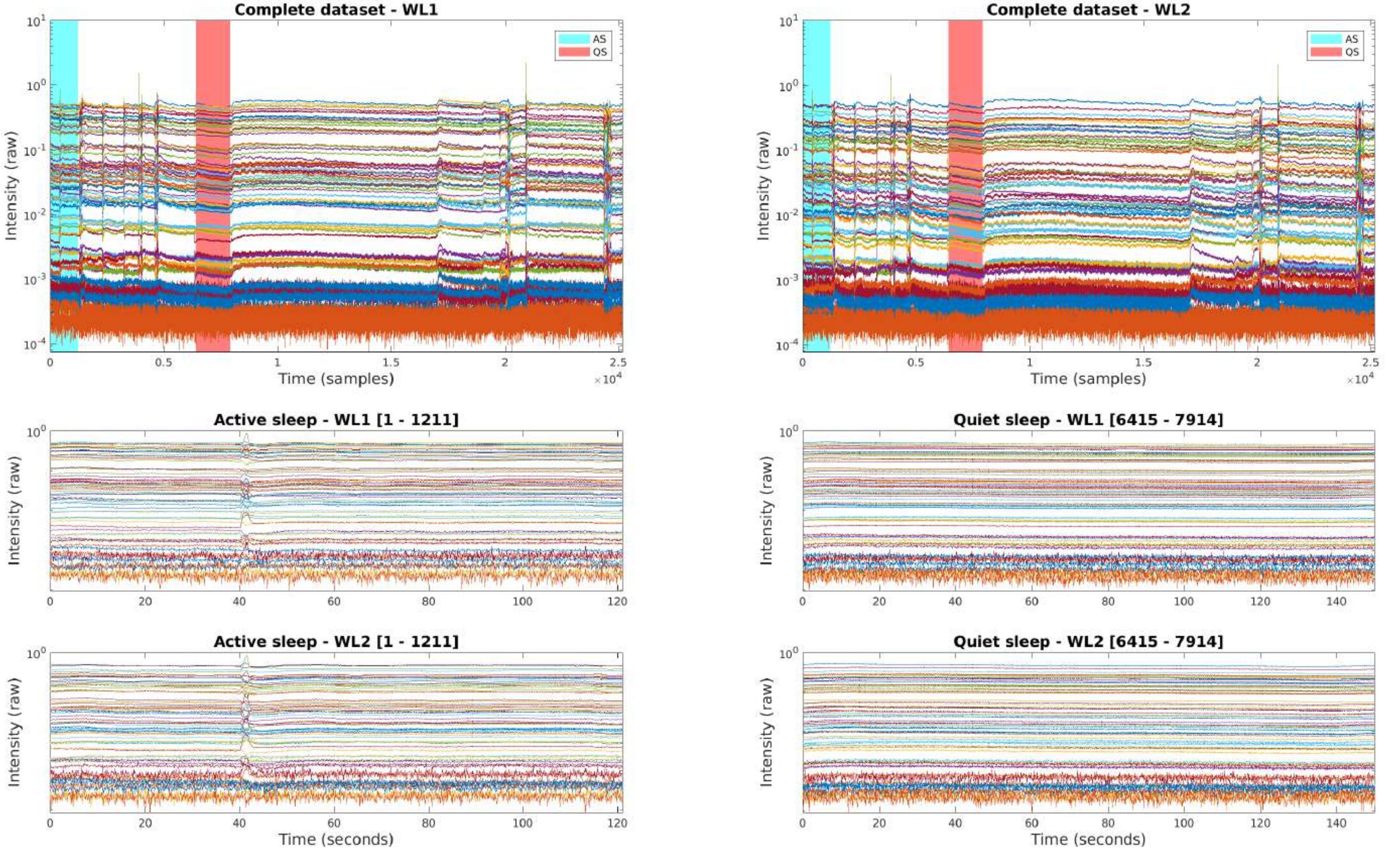
Complete dataset - WL1
Intensity (raw)
Time (samples)
AS
QS
Complete dataset - WL2
Active sleep - WL1 [1 - 1211]
Quiet sleep - WL1 [6415 - 7914]
Active sleep - WL2 [1 - 1211]
Quiet sleep - WL2 [6415 - 7914]
Time (seconds)

HT_026

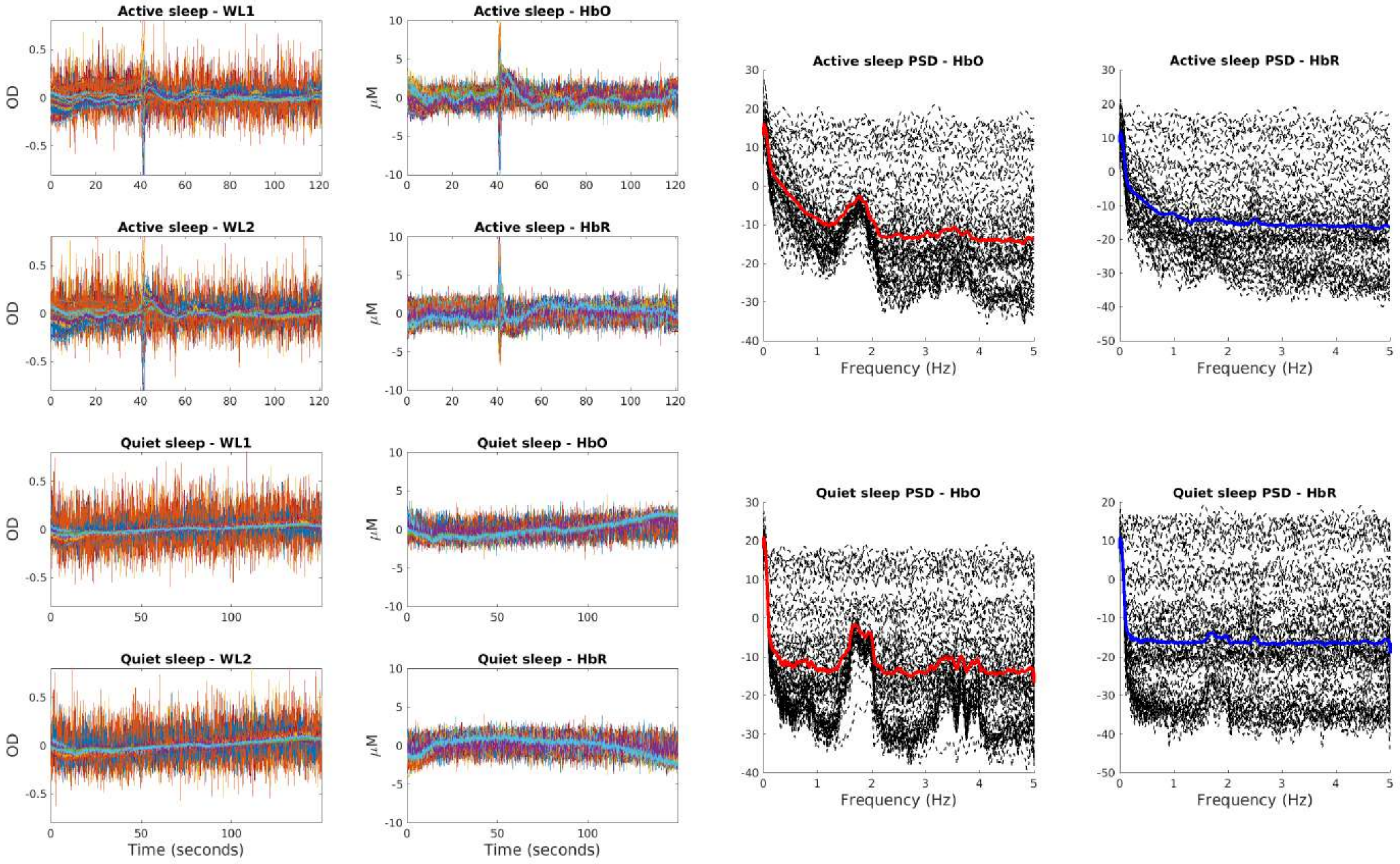

Active sleep - WL1
Active sleep - HbO
Active sleep PSD - HbO
Active sleep PSD - HbR
Active sleep - WL2
Active sleep - HbR
Quiet sleep - WL1
Quiet sleep - HbO
Quiet sleep PSD - HbO
Quiet sleep PSD - HbR
Quiet sleep - WL2
Quiet sleep - HbR
OD
μM
Time (seconds)
Frequency (Hz)

HT_026

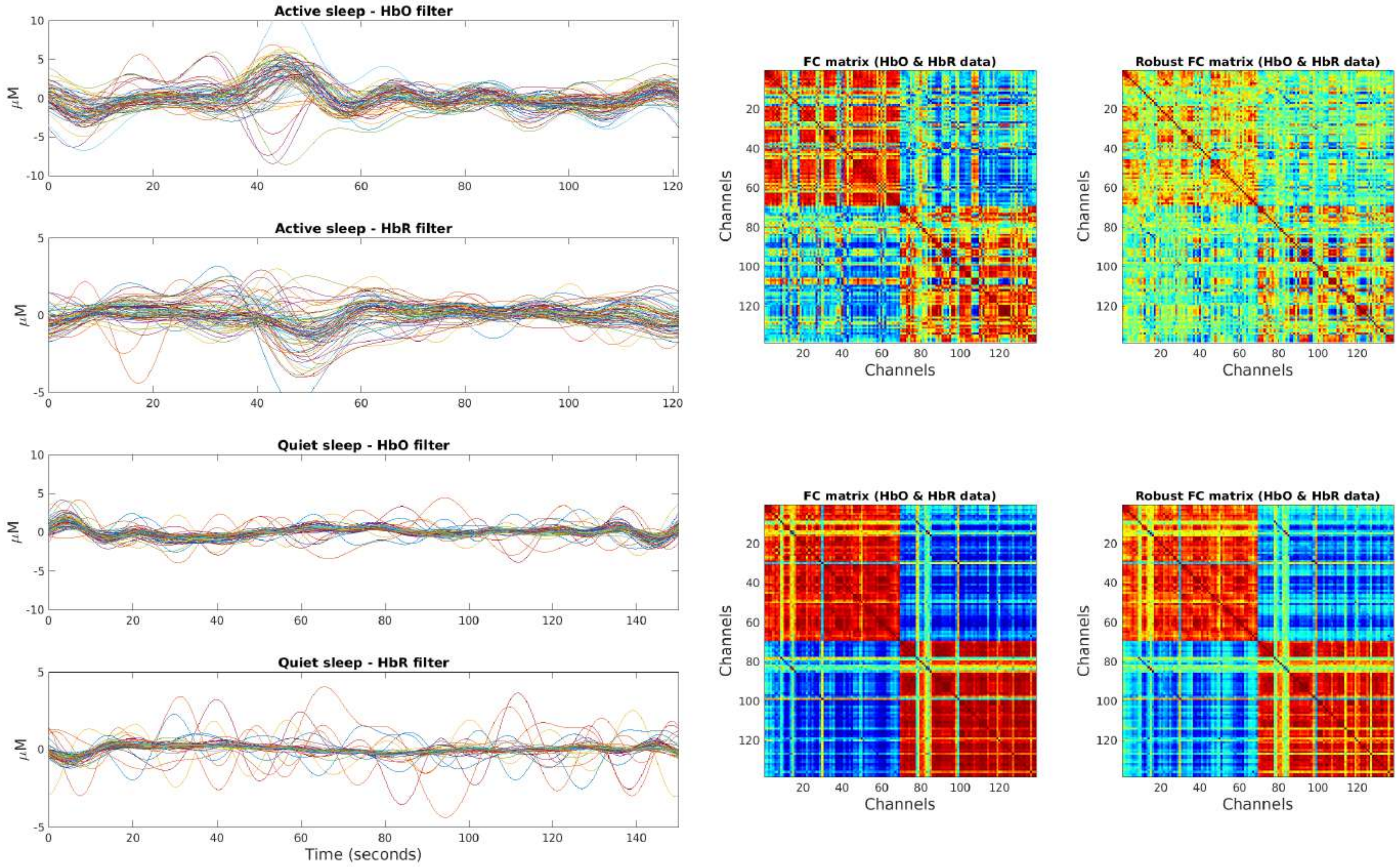

Active sleep - HbO filter
Active sleep - HbR filter
Quiet sleep - HbO filter
Quiet sleep - HbR filter
μM
Time (seconds)
FC matrix (HbO & HbR data)
Robust FC matrix (HbO & HbR data)
Channels

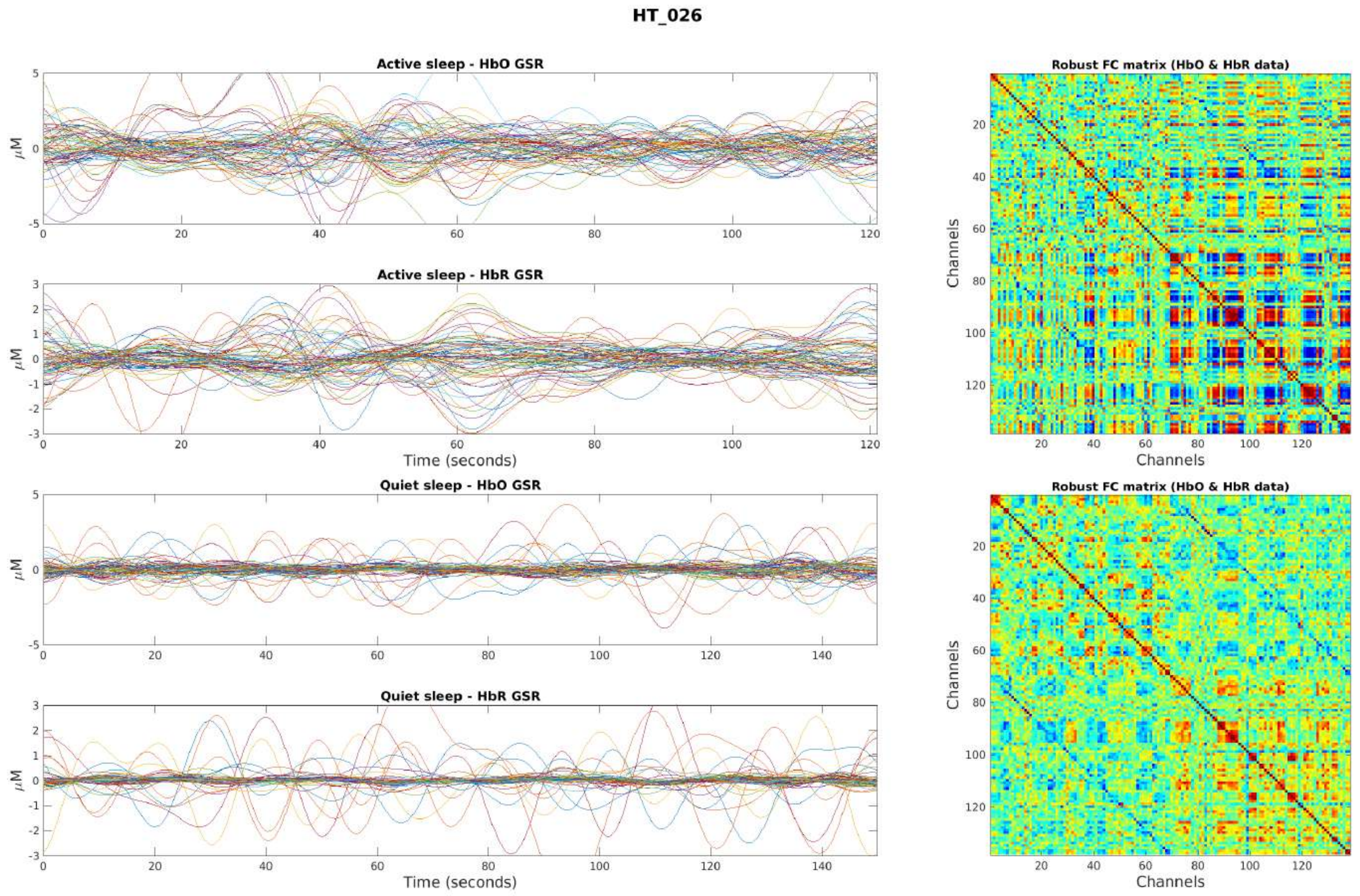
HT_026
Active sleep - HbO GSR
Active sleep - HbR GSR
Quiet sleep - HbO GSR
Quiet sleep - HbR GSR
μM
Time (seconds)
Robust FC matrix (HbO & HbR data)
Channels
Channels

HT_029

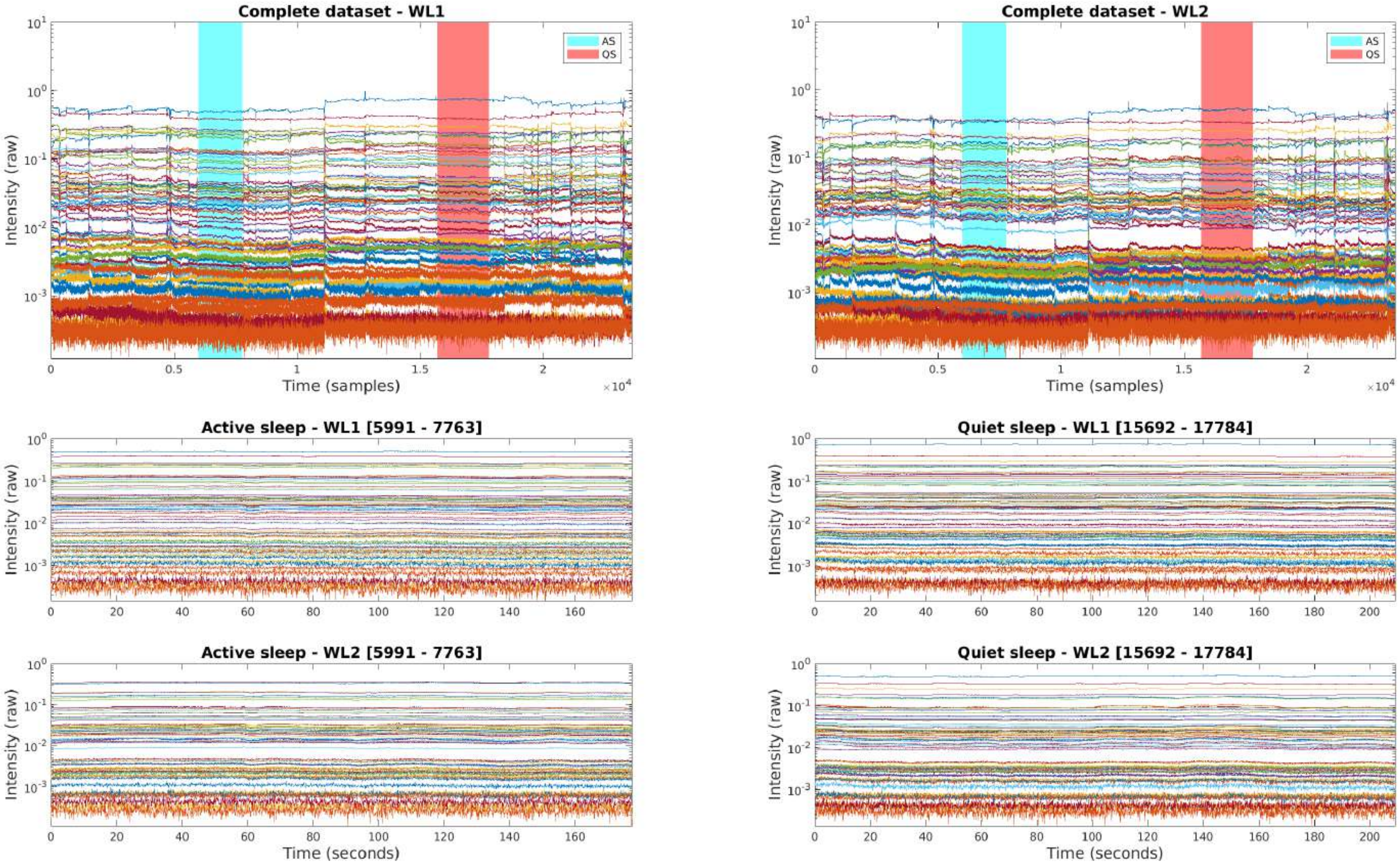
Complete dataset - WL1
Complete dataset - WL2
AS
QS
Intensity (raw)
Time (samples)
×10^4
Active sleep - WL1 [5991 - 7763]
Quiet sleep - WL1 [15692 - 17784]
Active sleep - WL2 [5991 - 7763]
Quiet sleep - WL2 [15692 - 17784]
Time (seconds)

HT_029

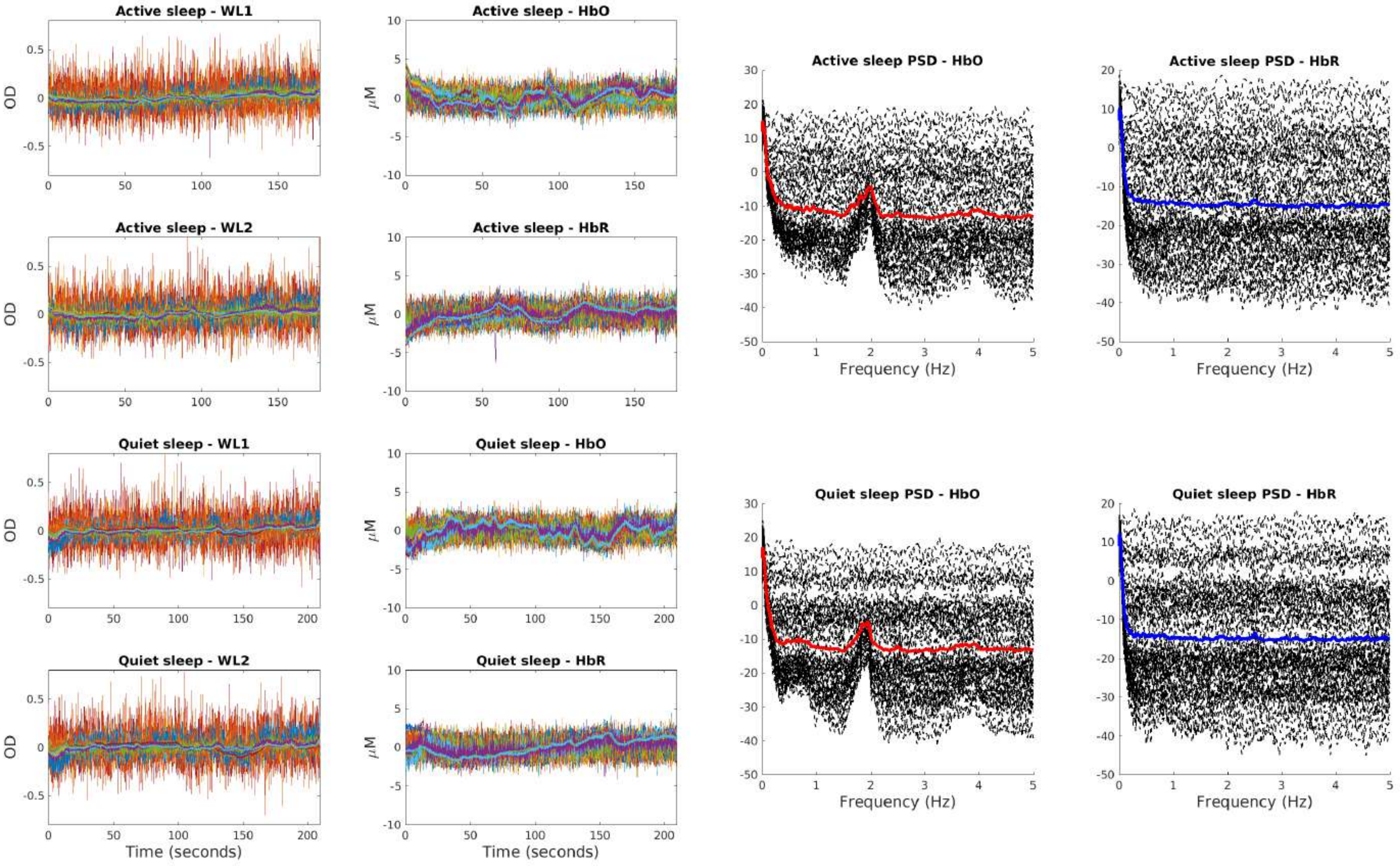

Active sleep - WL1
Active sleep - HbO
Active sleep PSD - HbO
Active sleep PSD - HbR
Active sleep - WL2
Active sleep - HbR
Quiet sleep - WL1
Quiet sleep - HbO
Quiet sleep PSD - HbO
Quiet sleep PSD - HbR
Quiet sleep - WL2
Quiet sleep - HbR
OD
μM
Time (seconds)
Frequency (Hz)

HT_029

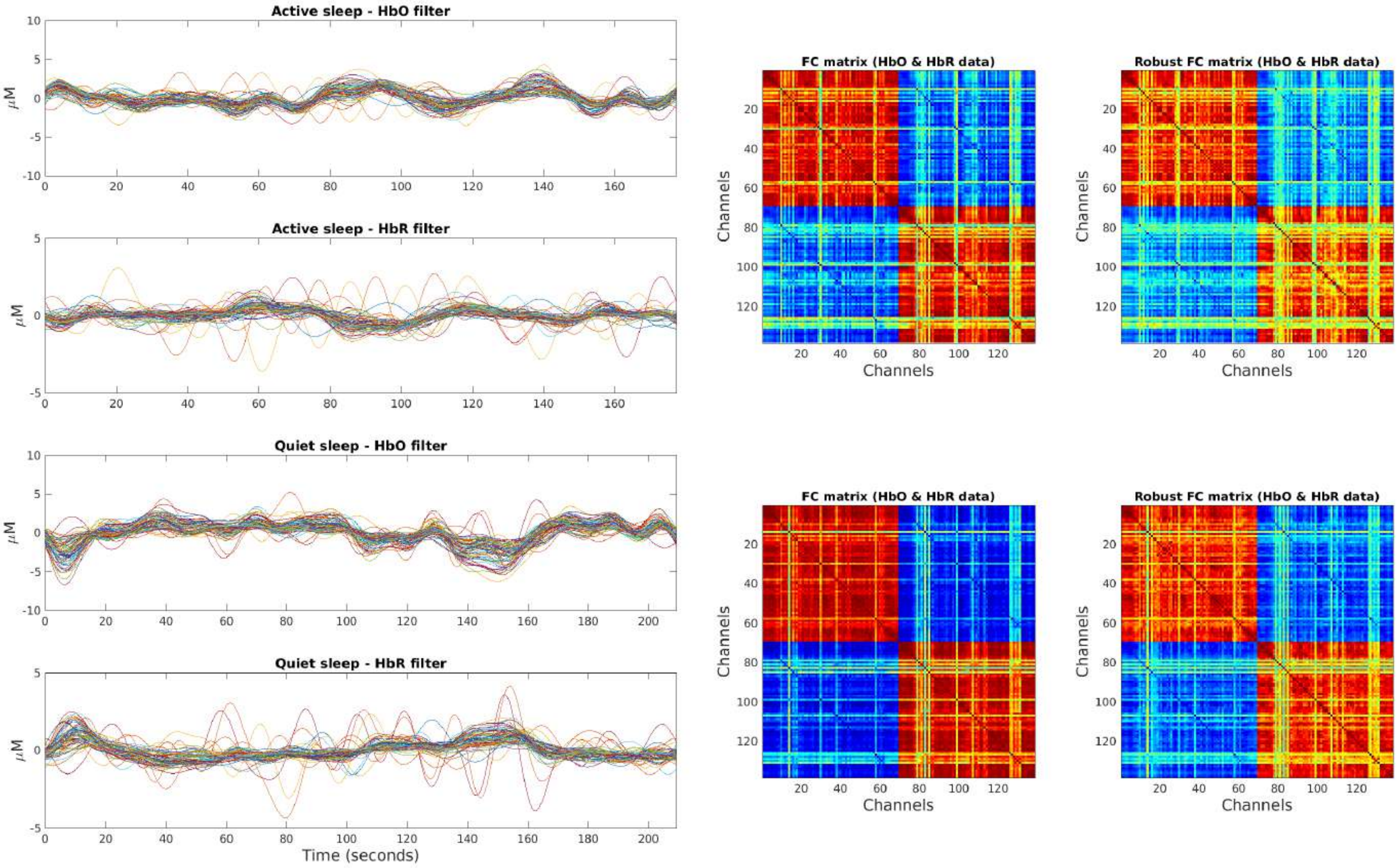

Active sleep - HbO filter
Active sleep - HbR filter
Quiet sleep - HbO filter
Quiet sleep - HbR filter
μM
Time (seconds)
FC matrix (HbO & HbR data)
Robust FC matrix (HbO & HbR data)
FC matrix (HbO & HbR data)
Robust FC matrix (HbO & HbR data)
Channels

HT_029

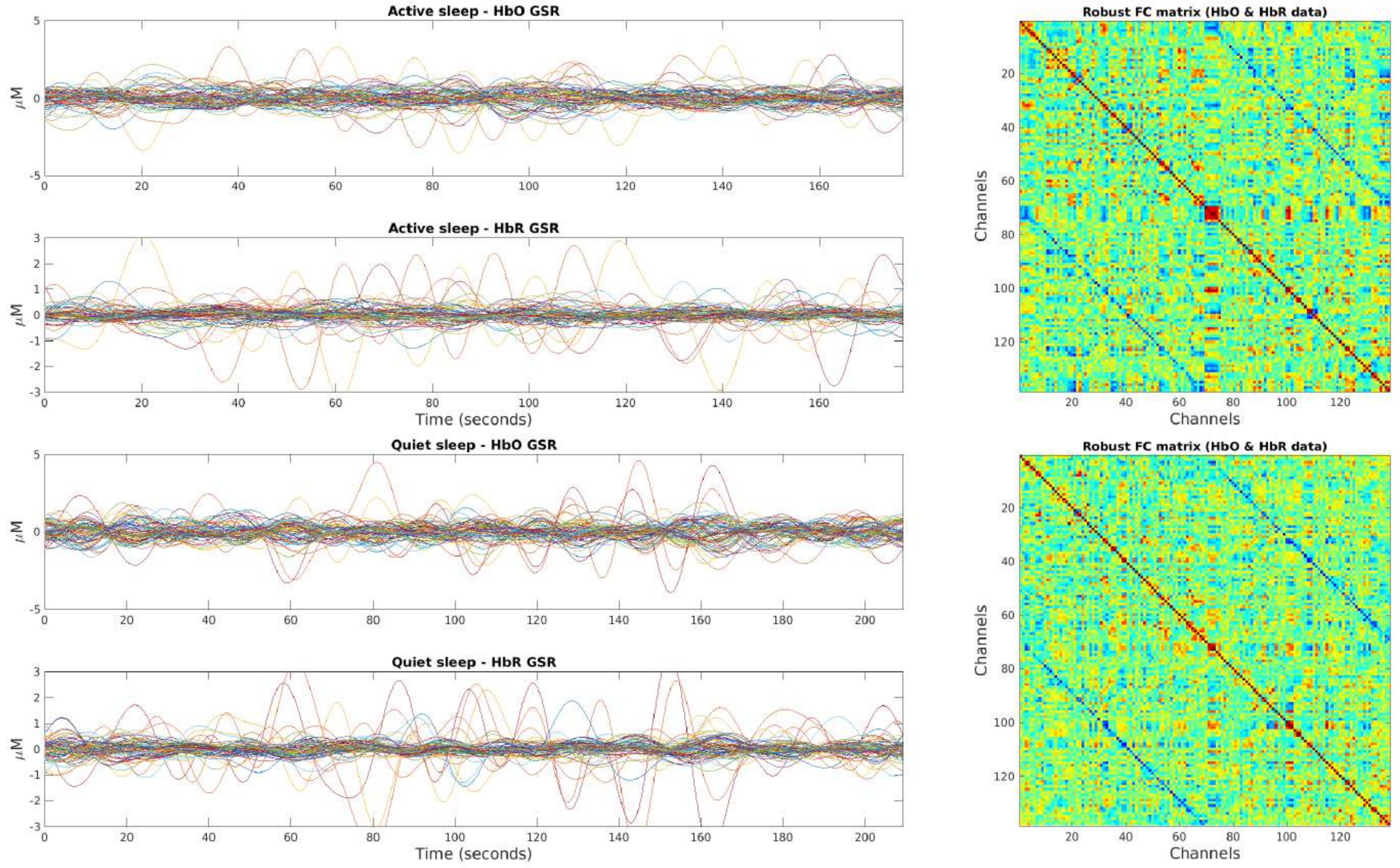

Active sleep - HbO GSR
Active sleep - HbR GSR
Quiet sleep - HbO GSR
Quiet sleep - HbR GSR
μM
Time (seconds)
Robust FC matrix (HbO & HbR data)
Channels

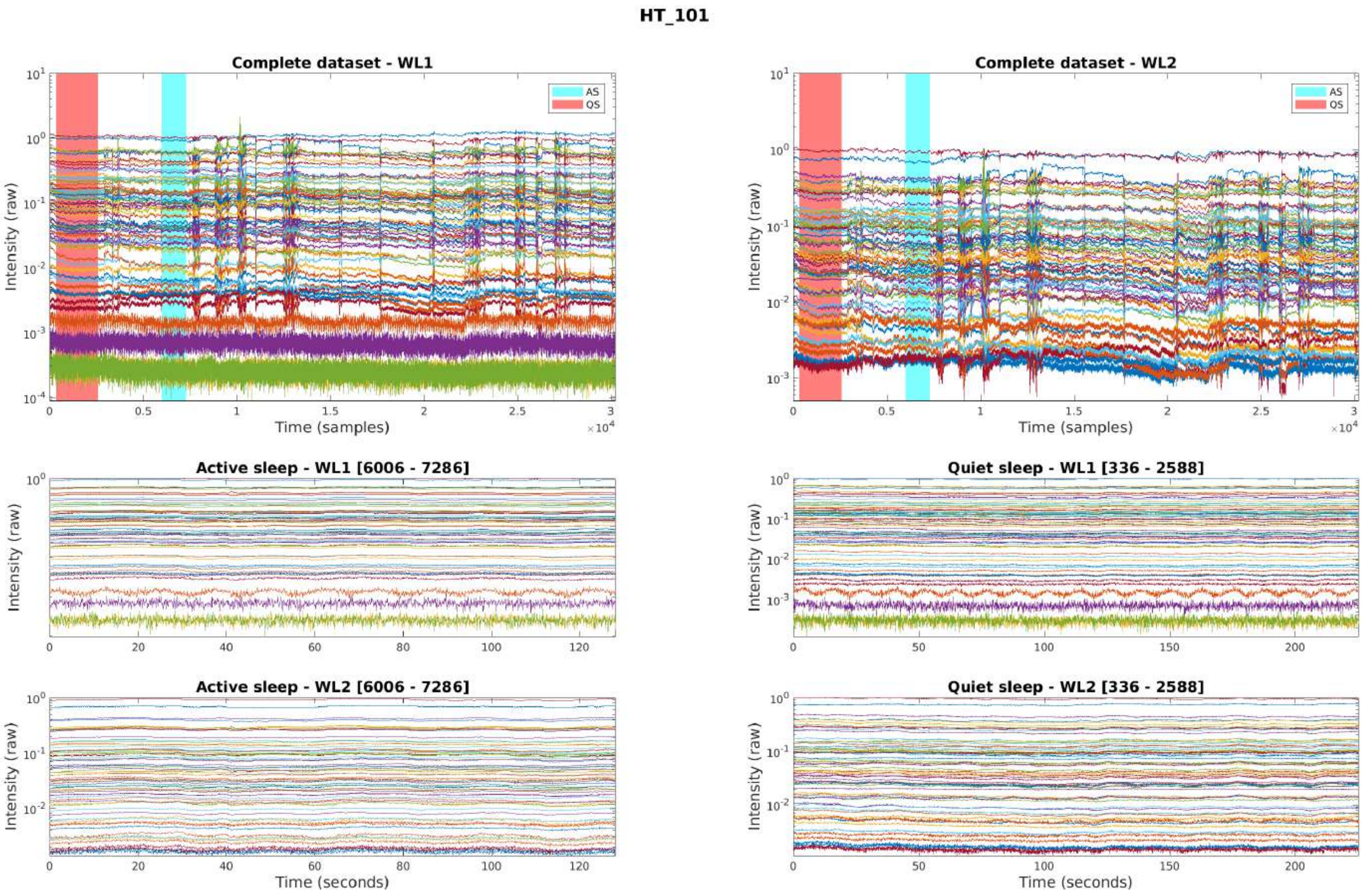
HT_101
Complete dataset - WL1
Complete dataset - WL2
AS
QS
Intensity (raw)
Time (samples)
$\times 10^4$
Active sleep - WL1 [6006 - 7286]
Quiet sleep - WL1 [336 - 2588]
Active sleep - WL2 [6006 - 7286]
Quiet sleep - WL2 [336 - 2588]
Time (seconds)

HT_101

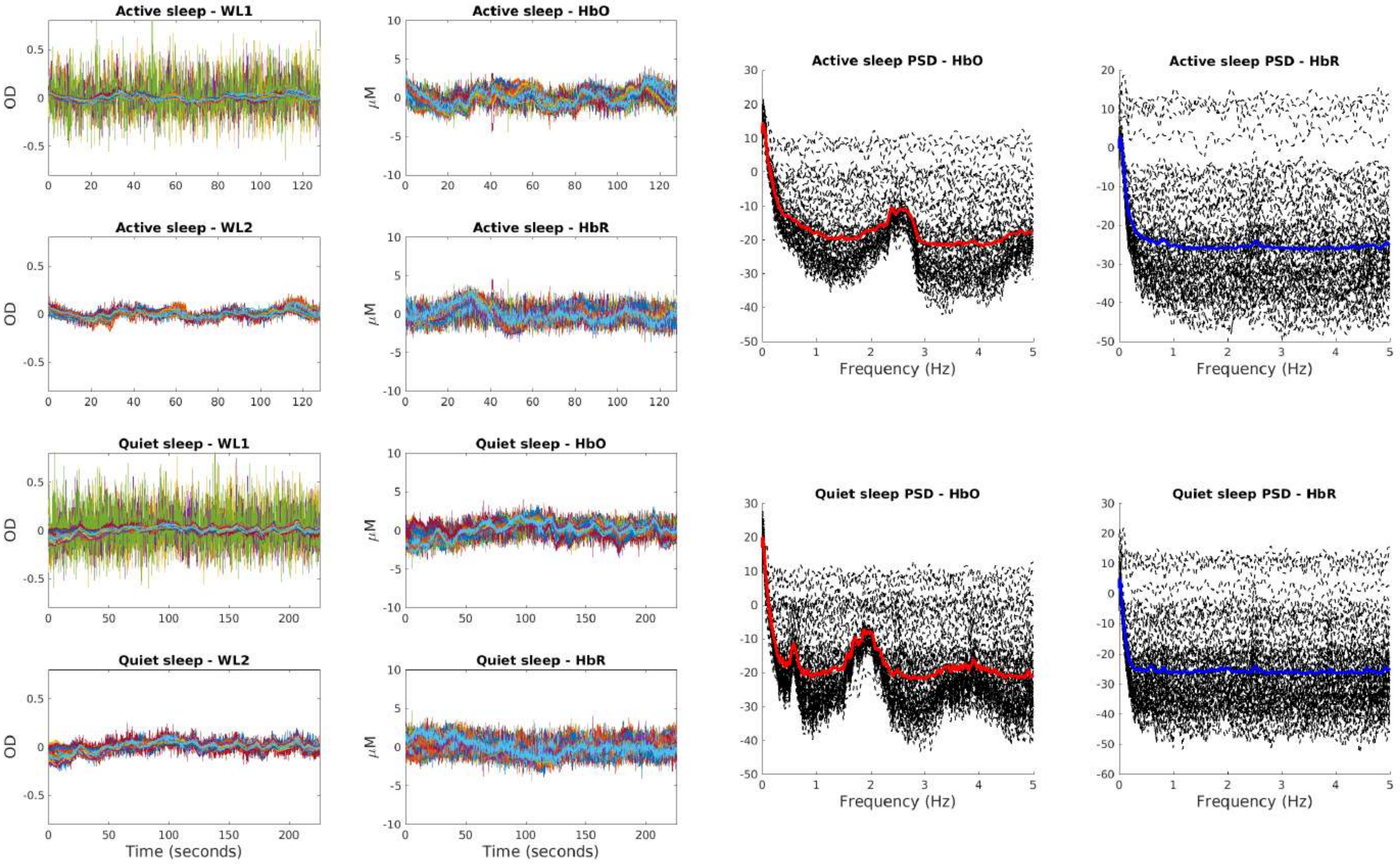
Active sleep - WL1
Active sleep - HbO
Active sleep PSD - HbO
Active sleep PSD - HbR
Active sleep - WL2
Active sleep - HbR
Quiet sleep - WL1
Quiet sleep - HbO
Quiet sleep PSD - HbO
Quiet sleep PSD - HbR
Quiet sleep - WL2
Quiet sleep - HbR
OD
μM
Frequency (Hz)
Time (seconds)

HT_101

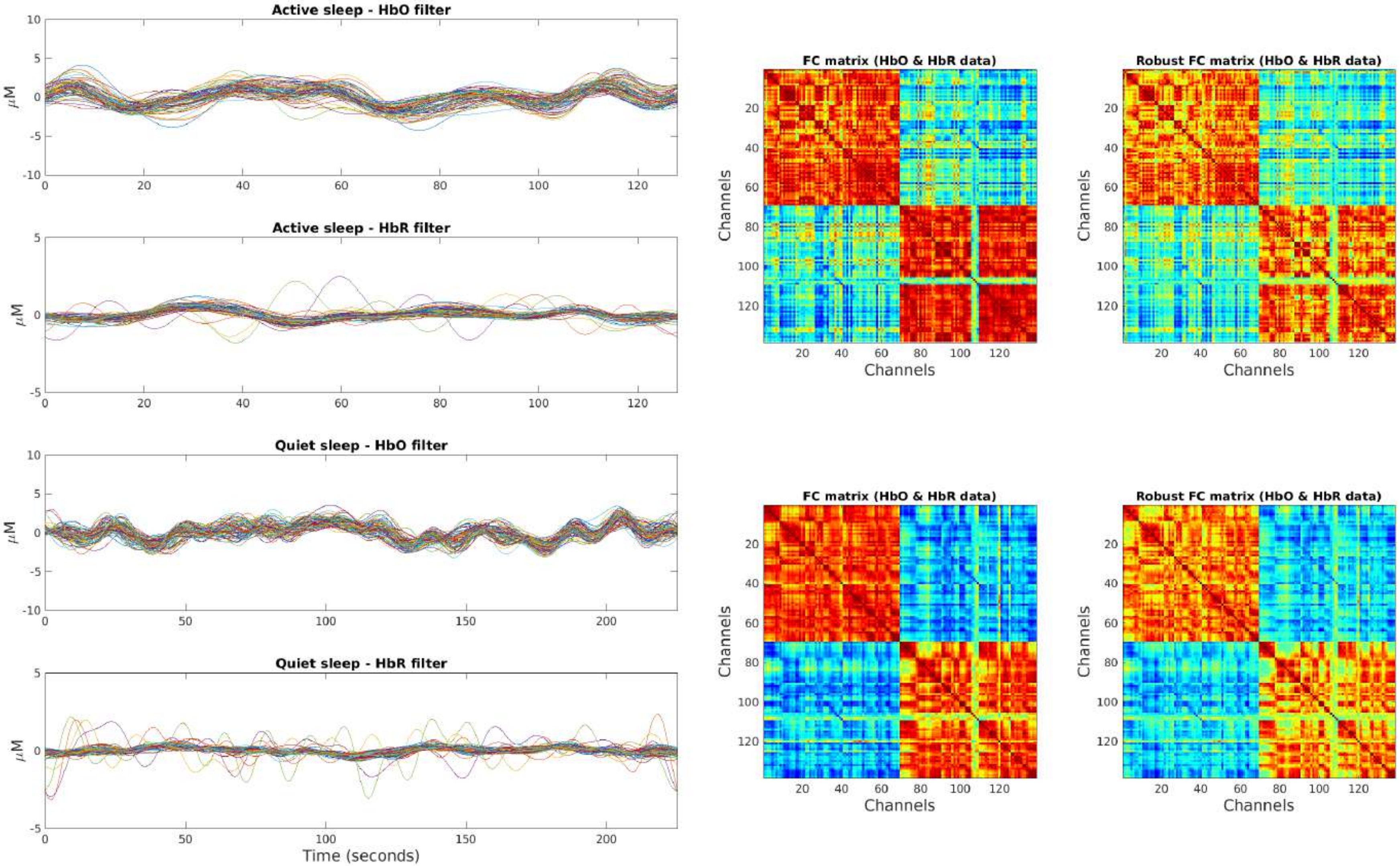

Active sleep - HbO filter
Active sleep - HbR filter
Quiet sleep - HbO filter
Quiet sleep - HbR filter
μM
Time (seconds)
FC matrix (HbO & HbR data)
Robust FC matrix (HbO & HbR data)
Channels

HT_101

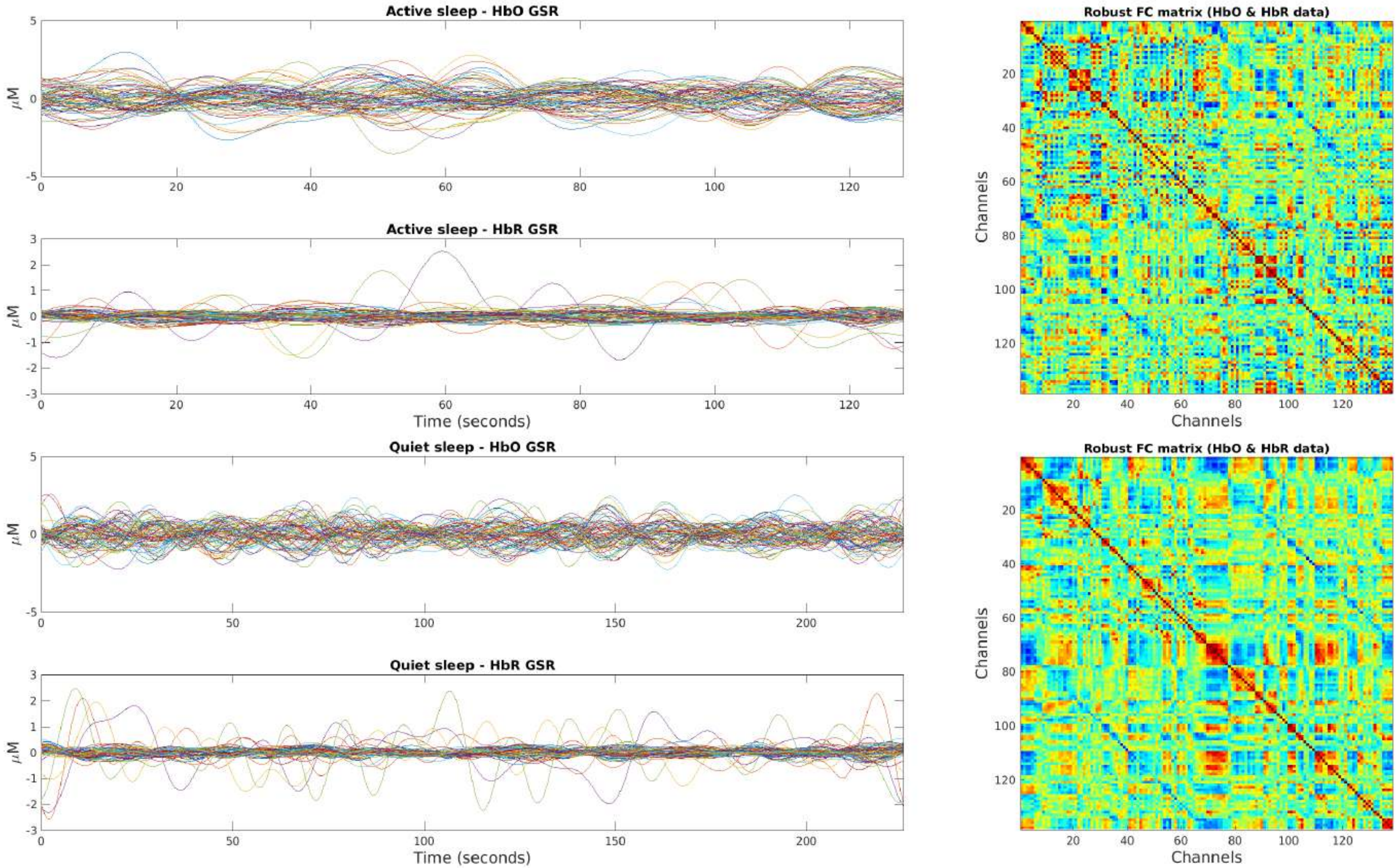

Active sleep - HbO GSR
Active sleep - HbR GSR
Quiet sleep - HbO GSR
Quiet sleep - HbR GSR
μM
Time (seconds)
Robust FC matrix (HbO & HbR data)
Channels
