# Supplementary material for: Sleep State Modulates Resting-State Functional Connectivity in Neonates
Source: Front Neurosci. 2020 Apr 17;14:347. doi: 10.3389/fnins.2020.00347 (PMC7180180; doi:10.3389/fnins.2020.00347)
Supplement: Supplementary file 2 [file Data_Sheet_2.PDF]

HbR

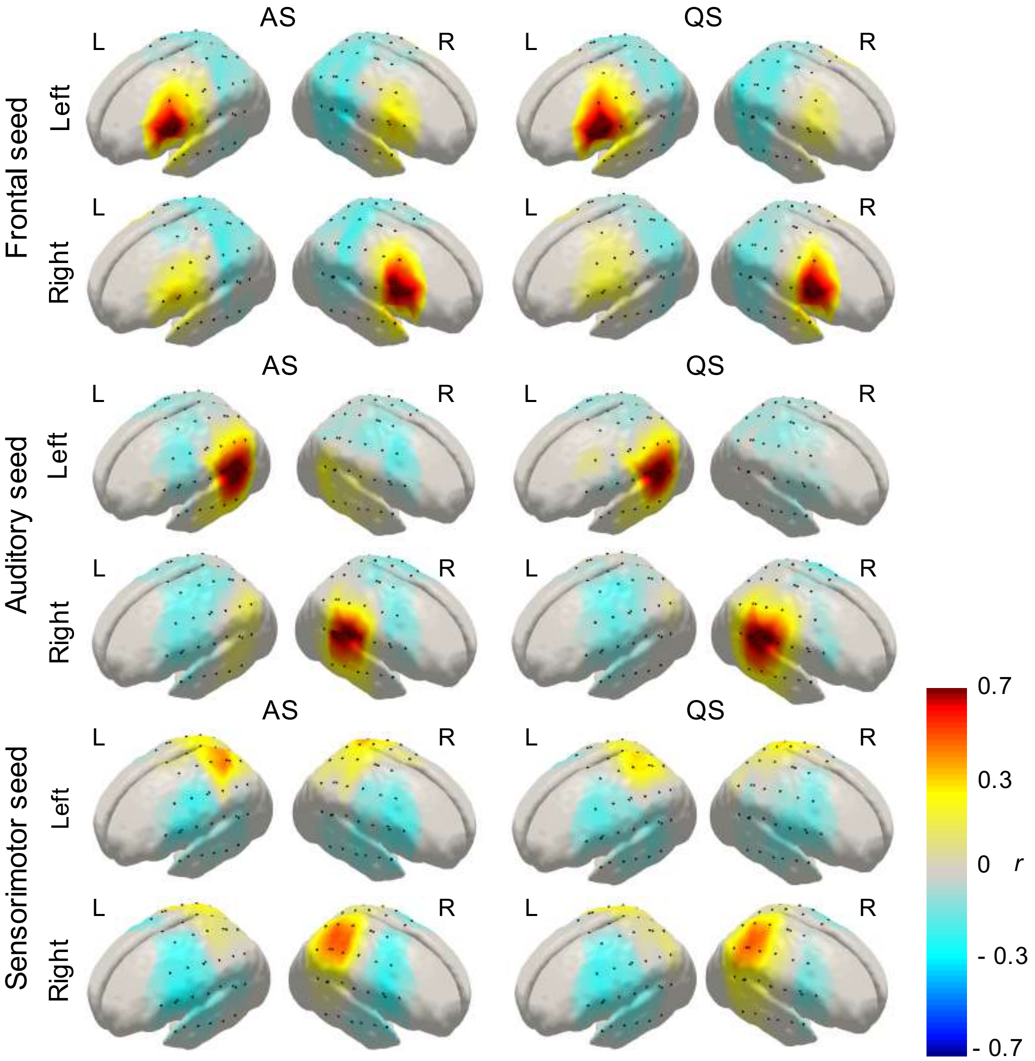

Supplementary figure 1. Average correlation maps (HbR) derived from the seed-based correlation method for the left and right seeds in the frontal, auditory and sensorimotor regions (AS = active sleep, first column; QS = quiet sleep, second column).

connICA HbO – Network 1

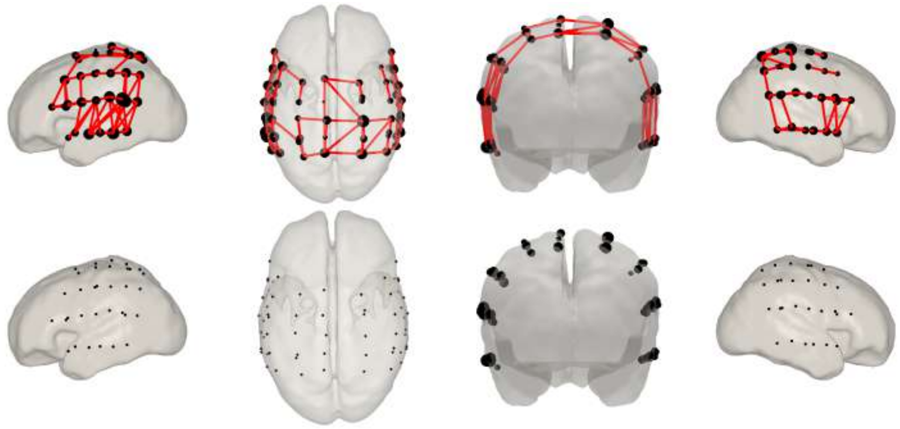

connICA HbO – Network 2

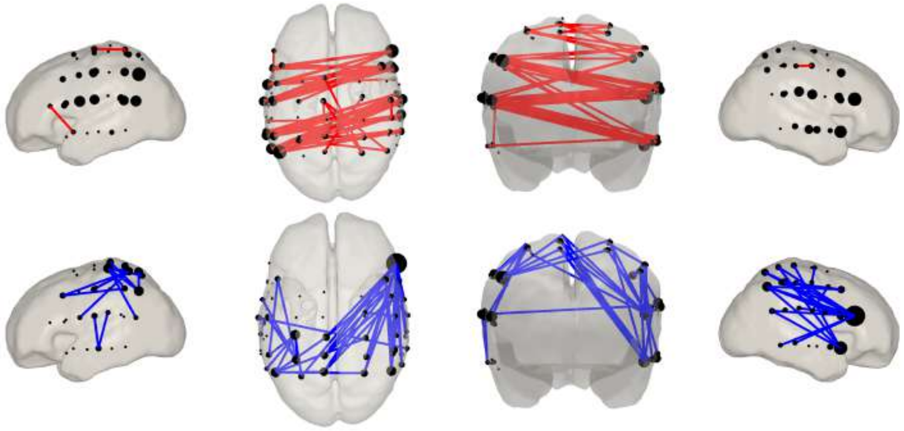

connICA HbO – Network 3

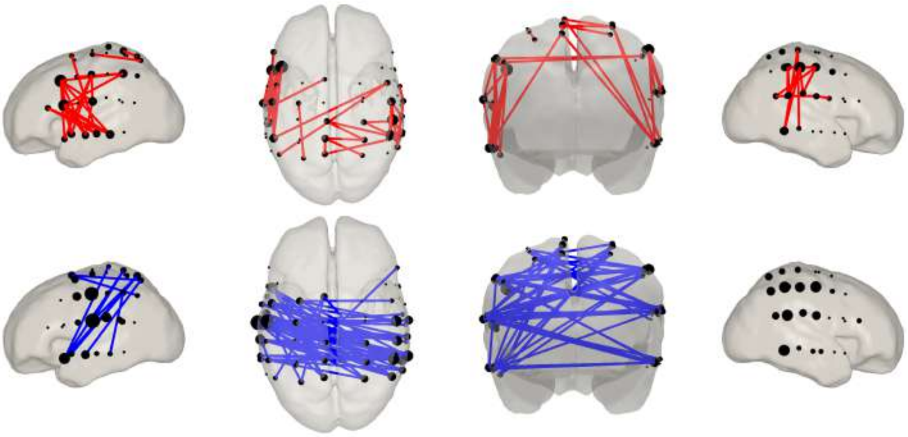

connICA HbO – Network 4

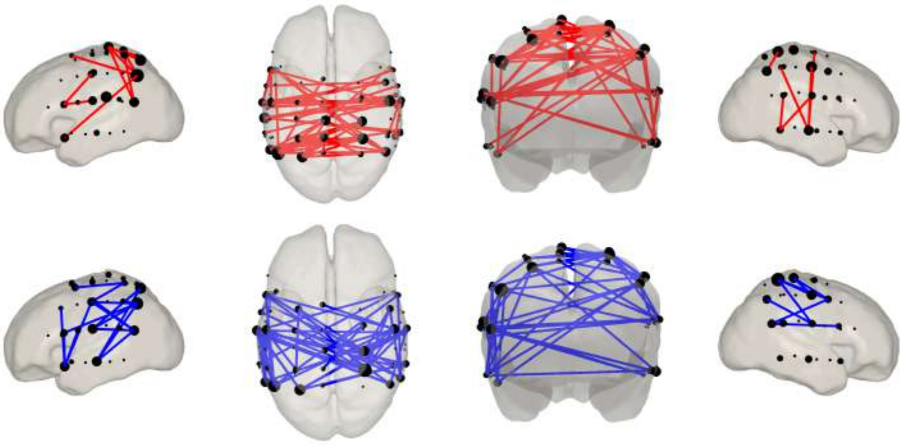

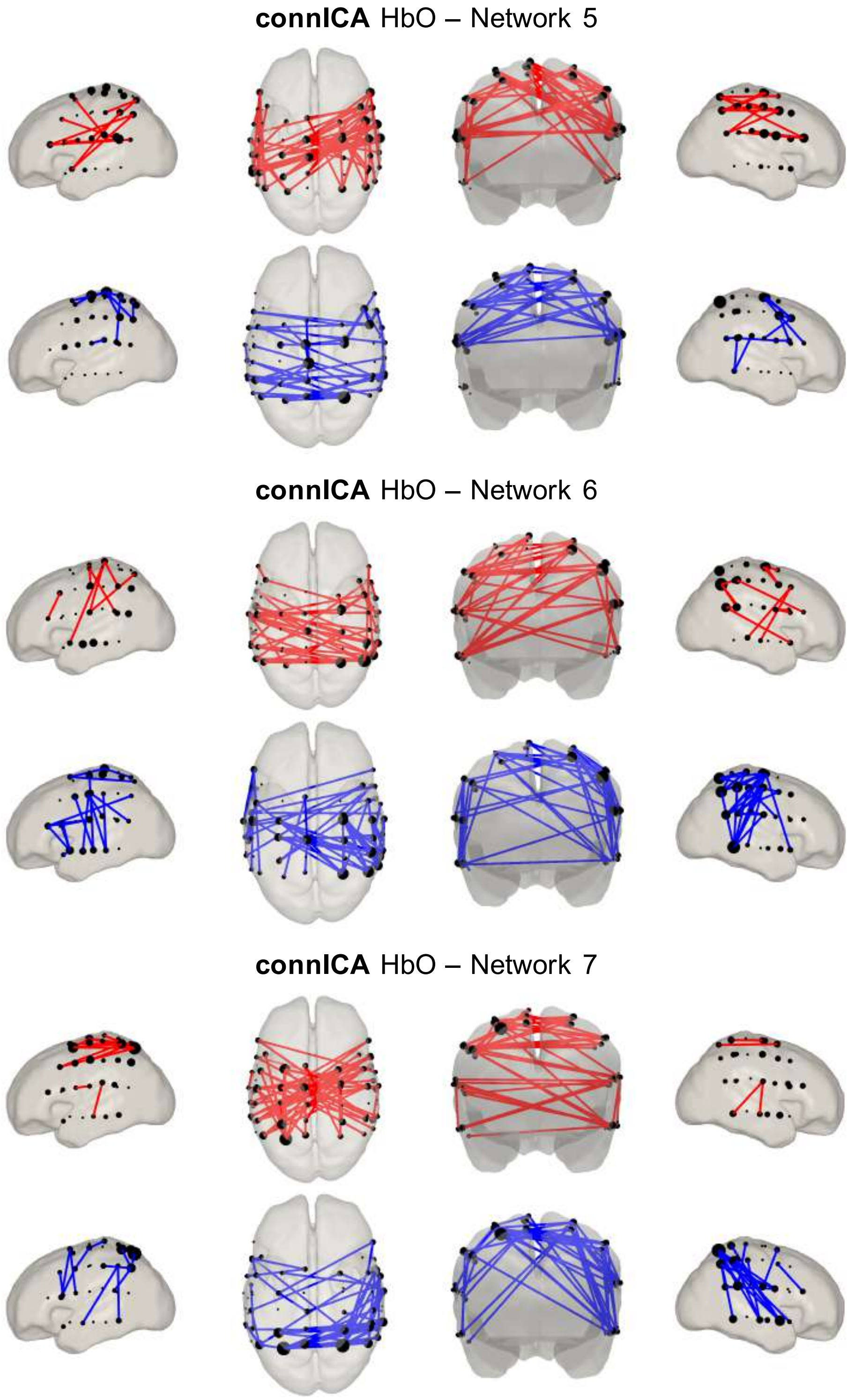

Supplementary figure 2. Results of ConnICA method for HbO. This analysis revealed 7 independent functional components. In this figure the top 5% connections of the identified networks are displayed (positive and negative connections). A high correspondence between the components identified across HbO and HbR is observed. Networks displaying significant differences between sleep states are discussed in the main text (networks 2 and 3).

connICA HbR – Network 1

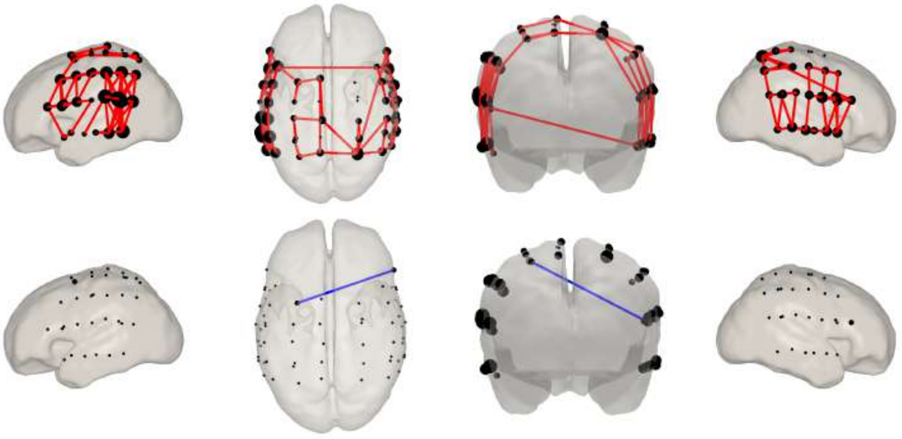

connICA HbR – Network 2

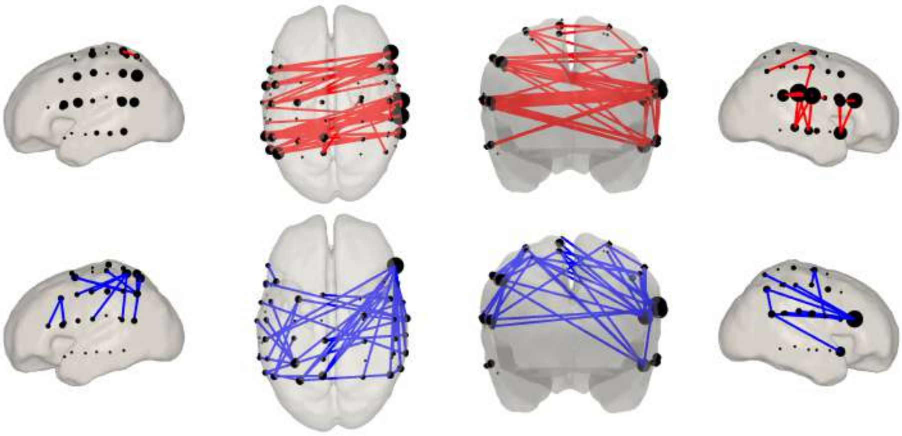

connICA HbR – Network 3

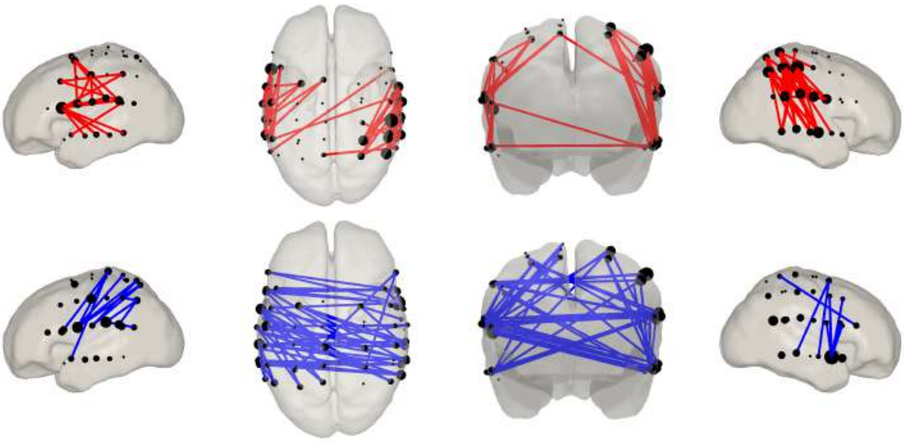

connICA HbR – Network 4

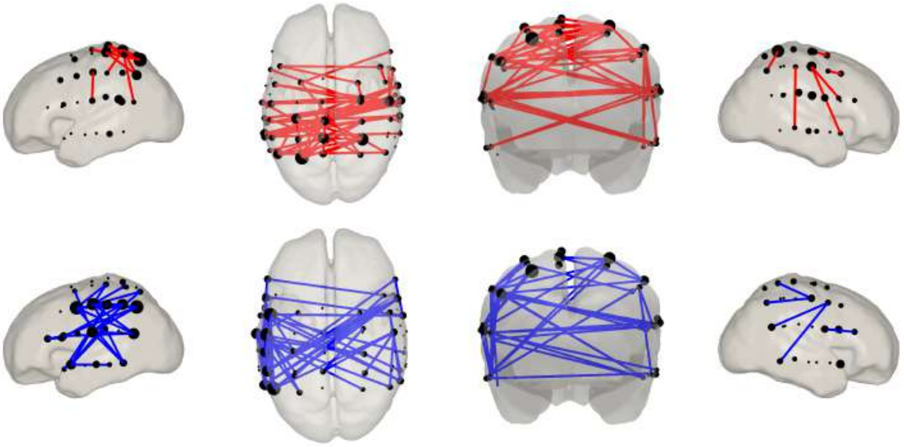

connICA HbR – Network 5

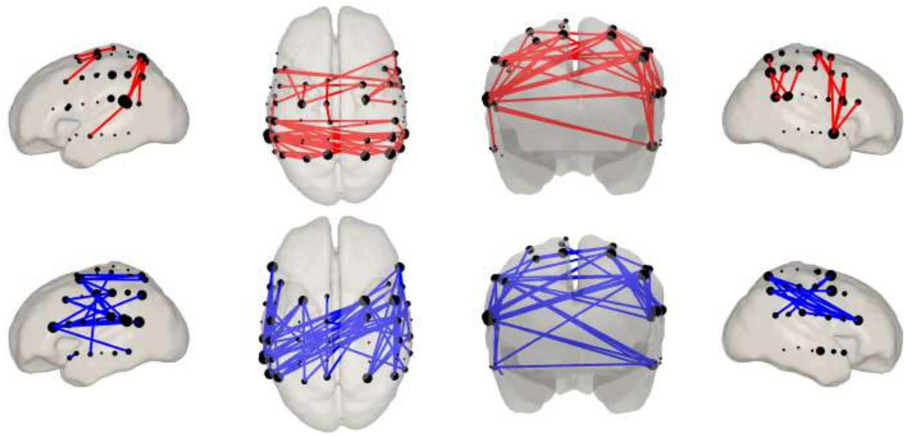

connICA HbR – Network 6

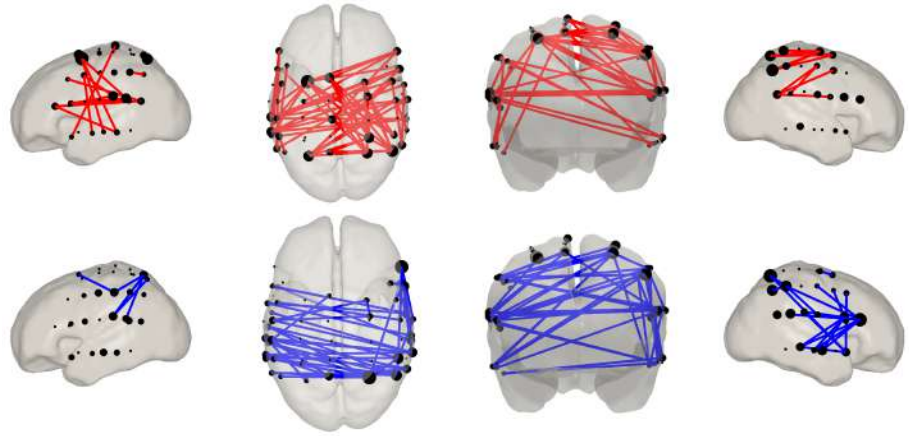

connICA HbR – Network 7

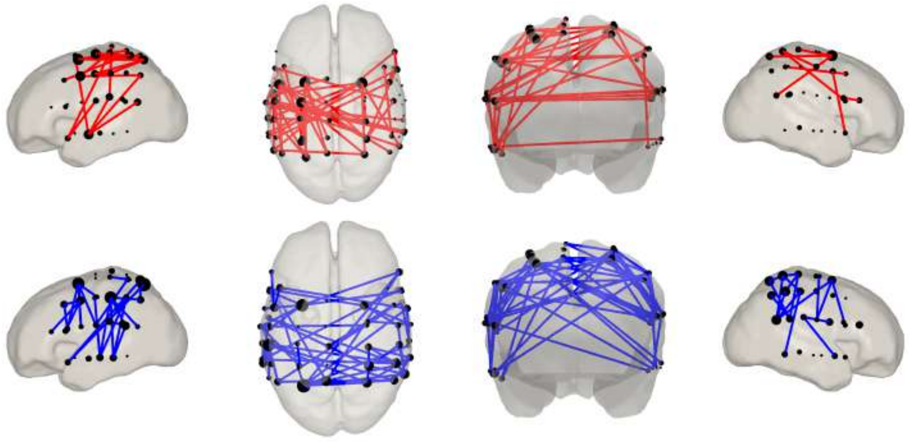

Supplementary figure 3. Results of ConnICA method for HbR. This analysis revealed 7 independent functional components. In this figure the top 5% connections of the identified networks are displayed (positive and negative connections). A high correspondence between the components identified across HbO and HbR is observed. Networks displaying significant differences between sleep states are discussed in the main text (networks 2 and 3).
